# Exploring the role of the disulfidptosis-related gene SLC7A11 in adrenocortical carcinoma: implications for prognosis, immune infiltration, and therapeutic strategies

**DOI:** 10.1186/s12935-023-03091-6

**Published:** 2023-11-02

**Authors:** Tonghu Liu, Yilin Ren, Qixin Wang, Yu Wang, Zhiyuan Li, Weibo Sun, Dandan Fan, Yongkun Luan, Yukui Gao, Zechen Yan

**Affiliations:** 1 Department of Surgery, The First Affiliated Hospital of Zhengzhou University, 450001 Zhengzhou, Henan, China; 2 BGI College & Henan Institute of Medical and Pharmaceutical Sciences, Zhengzhou University, 450001 Zhengzhou, Henan, China; 3 Henan Engineering Research Center of Tumour Molecular Diagnosis and Treatment, 450001 Zhengzhou, Henan, China; 4 Institute of Molecular Cancer Surgery of Zhengzhou University, 450001 Zhengzhou, Henan, China; 5 Department of Surgery, Nanyang Central Hospital, 473005 Nanyang, Henan, China; 6 Department of Radiation Oncology and Oncology, Henan Provincial People’s Hospital & the People’s Hospital of Zhengzhou University, 450003 Zhengzhou, Henan, China; 7 Department of Urology, The First Affiliated Hospital of Wannan Medical College, Yijishan Hospital, 241001 Wuhu, Anhui, China

**Keywords:** SLC7A11, Disulfidptosis, Adrenocortical carcinoma, Immune infiltration, Prognosis, Metabolic vulnerability, Treatment

## Abstract

**Background:**

Disulfidptosis and the disulfidptosis-related gene SLC7A11 have recently attracted significant attention for their role in tumorigenesis and tumour management. However, its association with adrenocortical carcinoma (ACC) is rarely discussed.

**Methods:**

Differential analysis, Cox regression analysis, and survival analysis were used to screen for the hub gene SLC7A11 in the TCGA and GTEx databases and disulfidptosis-related gene sets. Then, we performed an association analysis between SLC7A11 and clinically relevant factors in ACC patients. Univariate and multivariate Cox regression analyses were performed to evaluate the prognostic value of SLC7A11 and clinically relevant factors. Weighted gene coexpression analysis was used to find genes associated with SLC7A11. Gene Ontology (GO) and Kyoto Encyclopedia of Genes and Genomes (KEGG) analyses and the LinkedOmics database were used to analyse the functions of SLC7A11-associated genes. The CIBERSORT and Xcell algorithms were used to analyse the relationship between SLC7A11 and immune cell infiltration in ACC. The TISIDB database was applied to search for the correlation between SLC7A11 expression and immune chemokines. In addition, we performed a correlation analysis for SLC7A11 expression and tumour mutational burden and immune checkpoint-related genes and assessed drug sensitivity based on SLC7A11 expression. Immunohistochemistry and RT‒qPCR were used to validate the upregulation of SLC7A11 in the ACC.

**Results:**

SLC7A11 is highly expressed in multiple urological tumours, including ACC. SLC7A11 expression is strongly associated with clinically relevant factors (M-stage and MYL6 expression) in ACC. SLC7A11 and the constructed nomogram can accurately predict ACC patient outcomes. The functions of SLC7A11 and its closely related genes are tightly associated with the occurrence of disulfidptosis in ACC. SLC7A11 expression was tightly associated with various immune cell infiltration disorders in the ACC tumour microenvironment (TME). It was positively correlated with the expression of immune chemokines (CXCL8, CXCL3, and CCL20) and negatively correlated with the expression of immune chemokines (CXCL17 and CCL14). SLC7A11 expression was positively associated with the expression of immune checkpoint genes (NRP1, TNFSF4, TNFRSF9, and CD276) and tumour mutation burden. The expression level of SLC7A11 in ACC patients is closely associated withcthe drug sensitivity.

**Conclusion:**

In ACC, high expression of SLC7A11 is associated with migration, invasion, drug sensitivity, immune infiltration disorders, and poor prognosis, and its induction of disulfidptosis is a promising target for the treatment of ACC.

**Supplementary Information:**

The online version contains supplementary material available at 10.1186/s12935-023-03091-6.

## Introduction

Adrenocortical carcinoma (ACC) is a malignant endocrine tumour that occurs in the adrenal cortex and accounts for approximately 14% of primary adrenal tumours [1]. It generally has specific biological characteristics, such as hormone activity and high aggressiveness [2]. The estimated incidence of ACC is extremely low, at approximately 0.5 to 2 per million people per year [3]. There are two peaks in incidence: children under 4 years of age and middle-aged persons between 40 and 50 years of age [4]. In clinical practice, ACC is often difficult to diagnose at an early stage, and patients often have localized, distant metastases by the time they develop clinical symptoms. Thus, ACC patients lose the opportunity to surgically remove the tumour. Therefore, the prognosis of ACC patients is poor, with a 5-year survival rate of 25% for ACC patients and a 5-year survival rate of less than 13% for advanced ACC patients [5, 6].

In the treatment of ACC patients, it is now generally accepted that localized ACC can be surgically removed and assisted with mitotane. For advanced and metastatic ACC patients, first-line treatment is based on the combination of mitotane or mitotane alone with chemotherapeutic agents such as etoposide, doxorubicin, and cisplatin [7]. Molecular targeted therapy and immunotherapy have been proposed as promising second-line treatments for ACC by some researchers. However, the insulin-like growth factor 1 receptor (IGF1R) inhibitor linsitinib and immune checkpoint inhibitors such as anti-PD-1 nivolumab and pembrolizumab have not been shown to have a significant effect in ACC-related studies [8–10]. Moreover, mitotane also has a long half-life, dose-limiting toxicity, and a narrow therapeutic window. Thus, there is still a lack of effective treatment for ACC patients. In this context, therapies that could induce cell death in a variety of ways, such as disulfidptosis, ferroptosis, pyroptosis, and apoptosis, are promising therapeutic approaches to improve survival.

Disulfidptosis is a previously uncharacterized form of cell death that is different from traditional apoptosis, ferroptosis, and cuproptosis. Disulfidptosis is a cell death pattern triggered by the accumulation of disulfides in cells, which causes disulfide stress and ultimately the collapse of actin cytoskeleton proteins [11]. Solute carrier family 7 member 11 (SLC7A11; also known as xCT) regulates cysteine uptake and intracellular disulfide synthesis [12, 13]. The accumulation of disulfides is highly toxic to cells. The reduced form of nicotinamide adenine dinucleotide phosphate (NADPH) serves as a key factor in countering this toxicity. In the absence of glucose, NADPH production by the pentose phosphate pathway is significantly reduced, resulting in the accumulation of disulfides in cells [11]. Therefore, high expression of SLC7A11 (SLC7A11^high^) and glucose starvation are important conditions leading to disulfidptosis. An increasing body of evidence suggests that advances in cell death patterns will not only improve our basic understanding of cellular homeostasis but also provide new insights into the application of targeting specific death patterns in diverse diseases such as cancer [11]. However, the mechanism and biological significance of disulfidptosis and its associated genes in ACC are rarely reported.

In this study, bioinformatics methods were used to analyse the expression of the disulfidptosis-related gene SLC7A11 in multiple urological tumours, including ACC, as well as the association between the expression of SLC7A11 and clinically relevant factors and prognostic factors in patients with ACC. Furthermore, weighted gene coexpression analysis was performed by the “WGCNA” R package to select the genes associated with SLC7A11. The functions of these genes were also predicted via GO and KEGG enrichment analysis and validated by the LinkedOmics database. We analysed the relationship between SLC7A11 expression and immune cell infiltration through CIBERSORT and the Xcell algorithm. The relationship between SLC7A11 expression and partial immune chemokines in ACC was also analysed using TISIDB data. Finally, the key R packages “corrplot”, “maftools”, and “oncoPredict” were utilized for conducting correlation analysis among SLC7A11 expression levels, immune checkpoint-related genes, tumor mutation burden, and drug sensitivity.

Overall, our results suggest that SLC7A11^high^ in ACC is strongly associated with tumour migration, invasion, immune infiltration disorders, drug sensitivity, and poor prognosis and that its induction of disulfidptosis offers new hope for the treatment of ACC patients. SLC7A11 could be a promising biomarker for the treatment and prognostic assessment of ACC.

## Materials and methods

### Data acquisition

The RNA sequencing data of 128 normal samples and 79 ACC samples were downloaded from the UCSC Xena platform (https://xena.ucsc.edu/) (Table S1). In addition, the clinical information of 77 patients with ACC was obtained from The Cancer Genome Atlas (TCGA) database (https://portal.gdc.cancer.gov/) (Table S2), and two ACC patients with incomplete clinical information were excluded from the clinically relevant study. Two sets of genes associated with disulfidptosis were obtained from previous studies (Table S3).

### Analysis of differentially expressed genes (DEGs)

Differential gene analysis was performed to analyse differences in gene expression between ACC and normal adrenal tissues via the “DESeq2” R package (| log2FoldChange) | > 1, adjusted P value < 0.05) and to identify the intersection of DEGs and genes associated with disulfidptosis (SLC7A11, INF2, CD2AP, PDLIM1, ACTN4, MYH9, MYH10, IQGAP1, FLNA, FLNB, TLN1, MYL6, ACTB, DSTN, and CAPZB).

### Survival and prognostic analysis

Univariate Cox regression analysis was performed to investigate the impact of genes (SLC7A11, MYL6, and ACTB) on the prognosis of ACC patients. ACC patients were classified into high and low-expression groups based on median tangent values. The Kaplan‒Meier curve constructed between the two groups was used to analyse the impact of gene expression on the overall survival of ACC patients. The TIMER database (https://cistrome.shinyapps.io/timer/) was used to verify the results of the survival analysis.

### Comparison of the SLC7A11 expression level in urological-related tumours

Differences in SLC7A11 expression in urinary system-related tumours (adrenocortical carcinoma, bladder urothelial carcinoma, kidney clear cell carcinoma, kidney chromophobe, kidney papillary cell carcinoma, prostate cancer, pheochromocytoma, testicular germ cell tumour) were investigated by the Wilcoxon rank sum test or X^2^ test in the TCGA and GTEx databases (Table S4).

### Correlation analysis between SLC7A11 and other clinical characteristics

The relationships between SLC7A11 expression and clinically relevant factors (age, sex, clinical grade, T-, N-, and M-stage, survival time, survival status, and the expression of ACTB and MYL6) were analysed by the “limma” R packages.The results were visualized using the R packages “ComplexHeatmap” and “ggpubr”. The age and gene expression (MYL6 and ACTB) subgroups of ACC patients were bounded by corresponding median values (> 49 and ≤ 49, 7.62 and ≤ 7.62, > 9.95 and ≤ 9.95).

### Establishment of a signature associated with prognosis

Univariate and multivariate Cox regression analyses were performed to assess the relationships among clinically relevant factors, SLC7A11 expression, and the survival time of ACC patients. A nomogram was constructed using clinically relevant factors (age, sex, clinical grade, T stage and N stage) and the expression level of SLC7A11. The signature associated with prognosis was validated by internal validation, DCA curves, and timeROC curves based on nomoRisk and the expression of SLC7A11. The “survival”, “regplot”, “rms”, “timeRoc” and “ggDCA” packages of R software were utilized in the procedure.

### Weighted gene coexpression network analysis

Using the median of SLC7A11 expression as a reference standard, patients were divided into an SLC7A11 high-expression group and an SLC7A11 low-expression group. Differential expression gene analysis was conducted using the “DESeq2” R package (| log2FoldChange) | > 0, adjusted P value < 0.05). Weighted gene coexpression analysis was performed using the “WGCNA” R package to identify the gene segment most closely associated with SLC7A11 and analyse its association with clinical phenotypes.

### Hub gene network construction and enrichment analysis of SLC7A11-interacted proteins

The STRING database (https://string-db.org/) and Cytoscape (V3.9.0) were utilized to generate the hub gene network of the target template. Furthermore, the association between these hub genes and genes related to disulfidptosis was investigated in ACC using the GEPIA2 database (http://gepia2.cancer-pku.cn/). The “clusterProfiler” and “org.Hs.eg.db” R packages were used for GO and KEGG enrichment analysis to assess the function of these genes.

### LinkedOmics database analysis

The LinkedOmics database (http://www.linkedomics.org/login.php) is a multiomics database that includes 32 cancer types and their clinical information. We analysed and demonstrated all coexpressed genes of SLC7A11 in a volcano plot and the top 50 genes with positive and negative correlations in a heatmap. In addition, we performed GO enrichment analysis using this database to validate previous results.

### Correlation analysis between SLC7A11 expression and immune cell infiltration

Patients were classified into high- and low-expression groups based on the median values of SLC7A11 expression. To assess the relationship between SLC7A11 expression and immune cell infiltration in ACC, the “CIBERSORT” and “Xcell” algorithms were used to calculate the levels of immune cell infiltration. Differences in immune cell infiltration levels between the high and low SLC7A11 expression groups were analysed, and the results are presented in a box diagram. The “reshape2”, “xCell”, “tidyr” “ggpubr”, “ggsci”, and “e1071” packages of R software were utilized in the procedure.

### Correlation analysis between SLC7A11 expression and immune chemokines

TISIDB data (http://cis.hku.hk/TISIDB/) is an integrated repository portal for tumour-immune system interactions. The relationships between SLC7A11 expression and important immune chemokines were analysed in this database.

### Correlation analysis of SLC7A11 expression and immune checkpoints and tumour mutation burden

To investigate the role of SLC7A11 in the treatment of ACC patients, we performed correlation analysis of SLC7A11 and immune checkpoint genes and tumour mutational burden. We first analysed the association between SLC7A11 and 47 immune checkpoint-related genes using the “corrplot” R package (Table S5). We then downloaded mutation data from ACC patients in the TCGA database, calculated tumour mutation burden (TMB) values using the “maftools” R package, and ultimately analysed the associations between SLC7A11 and ACC and TMB by Spearman correlation analysis.

### Drug sensitivity analysis

We employed the gene expression data from 79 ACC samples and the gene expression and drug response values from the training set to predict the sensitivity score of 198 anti-tumor drugs. This prediction was achieved using ridge regression models constructed with the R package “oncoPredict“ [14]. Subsequently, we divided ACC patients into two groups based on the median SLC7A11 expression value: high expression group and low expression group. The differences in sensitivity scores of the anti-tumor drugs between these two groups were investigated using the wilcox. test. Finally, the results were visualized using the R package “ggplot2”. The gene expression and drug response values of the training set were downloaded from Genomics of Drug Sensitivity in Cancer (GDSC, https://www.cancerrxgene.org/).

### Sample origin

Tissue samples were taken from patients undergoing adrenal or postperitoneal occupying resection at the First Affiliated Hospital of Zhengzhou University. All patients signed an informed consent form before using clinical materials. The study was approved by the Ethics Committee of the First Affiliated Hospital of Zhengzhou University (2022-KY-1035-001).

### Histopathology and immunohistochemical staining

For the histopathological assessment, paraffin sections were defatted in xylene and alcohol with gradient concentrations (Leica ASP200S). Paraffin sections were stained with haematoxylin dye for 3 min, followed by treatment with haematoxylin differentiation solution for 45 s. Finally, after two minutes of staining with eosin dye, the sections were sealed with neutral gum (Roche HE600).

For the immunohistochemical assessment, paraffin sections of 3 microns thick were placed in a 65 °C incubator for 2 h, followed by defatting in xylene two times for 15 min each. The sections were rehydrated with 100%, 95%, 85% and 70% alcohol. After 10 min of blocking endogenous peroxidase activity with 3% hydrogen peroxide, the slides were washed 3 times with PBS, and antigen repair was performed in thermal repair buffer. The slides were incubated in a closed buffer for 1 h to bind to nonspecific antibodies. Then, they were treated with SLC7A11 antibodies (Rabbit # DF-12,509, Affinity Biosciences, AUS) overnight and incubated with a second antibody for 1 h. Dyeing was performed first with DAB and then again with sulforaphane (Benchmark Ultra). The sections were then sealed with neutral gum, and positive staining was observed under a microscope.

### Reverse transcription-quantitative (RT-q) PCR

Total RNA was extracted from ACC samples and normal adrenal tissue using a reliable Total RNA isolation reagent (AmoyDx, Xiamen, China), followed by reverse transcription into cDNA using a high-quality Takara RT kit (RR047A, Takara, Tokyo, Japan). The reverse transcription reaction was conducted at 37°C for 15 min, followed by a denaturation step at 85°C for 5 sec, and cooling at 4°C. Subsequently, qPCR was performed using a trustworthy TB Green® Premix Ex Taq™ (RR820A,Takara, Tokyo, Japan) in a final volume of 20 µl, utilizing QuantStudio™ 5 (Applied Biosystems; Thermo Fisher Scientific, Inc.). The qPCR cycling conditions consisted of an initial denaturation at 95°C for 30 sec, followed by 40 cycles of denaturation at 95°C for 5 sec and annealing/elongation at 60°C for 34 sec. Gene expression was quantified using the widely accepted 2^(-ΔΔCq) method, with GAPDH serving as the internal control. The PCR primer sequences (Azenta, lnc.) for the target gene SLC7A11 were as follows: Forward primer: 5’-GGTCCATTACCAGCTTTTGTACG-3’ and Reverse primer: 5’-AATGTAGCGTCCAAATGCCAG-3’.

### Statistical analysis

Statistical analysis was performed using R software (version 4.2.1). The t test, Wilcoxon rank sum test, and one-way ANOVA were used to detect the differences between the groups. The survival analyses were determined by the Kaplan‒Meier curve, log-rank test, and Cox proportional hazard regression model. The correlation analysis was evaluated using Spearman correlation analysis. In all analyses, a *P* value < 0.05 indicated statistical significance; *, **, and *** indicate *P* < 0.05, *P* < 0.01, and *P* < 0.001, respectively.

## Results

### The disulfidptosis-related gene SLC7A11 was misregulated in cancers and related to prognosis in ACC

**Fig. 1:**
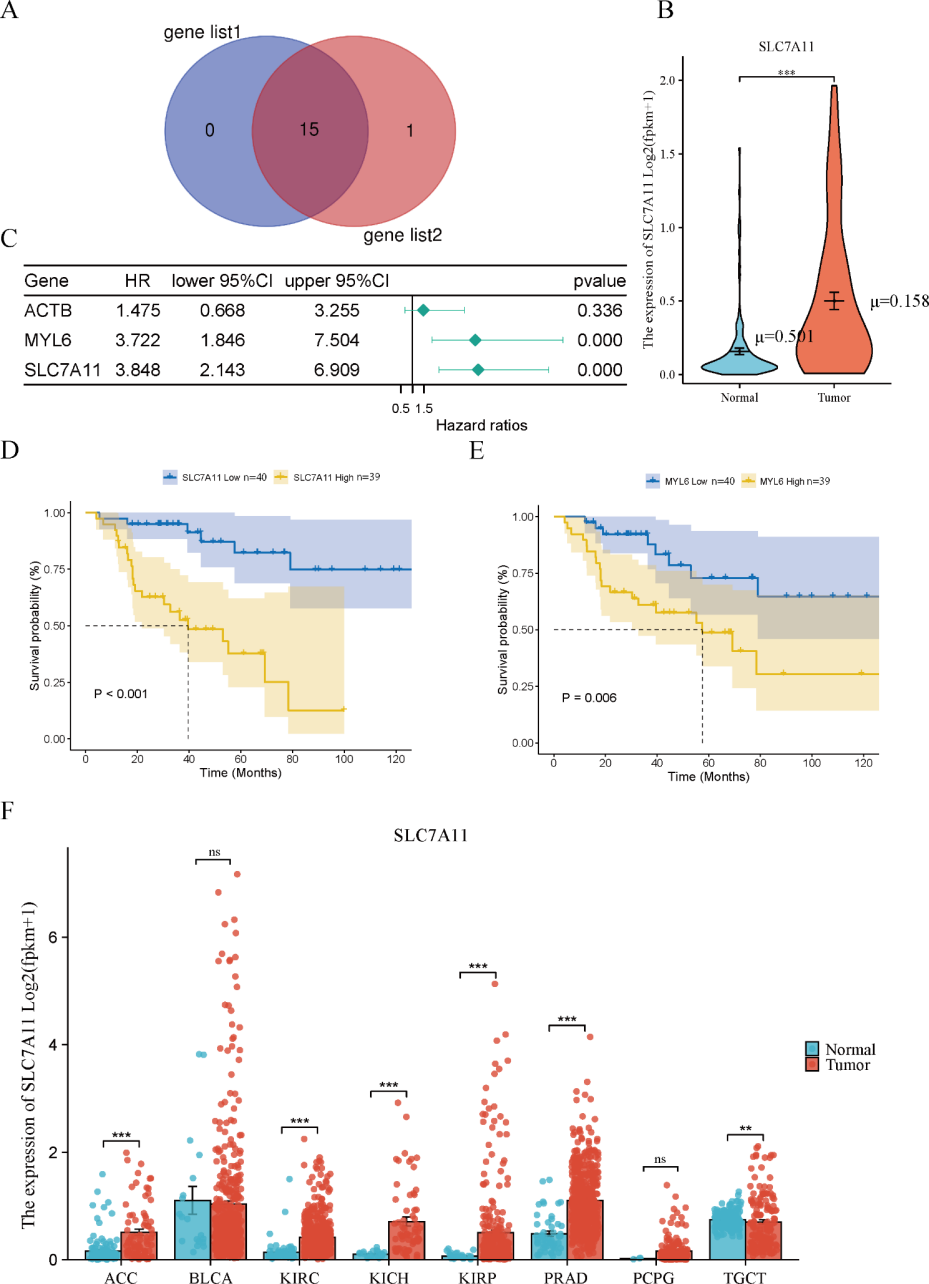
The disulfidptosis-related gene SLC7A11 was deregulated in cancers and related to prognosis in ACC. **A** Two sets of genes associated with disulfidptosis have 15 crossover genes: SLC7A11, INF2, CD2AP, PDLIM1, ACTN4, MYH9, MYH10, IQGAP1, FLNA, FLNB, TLN1, MYL6, ACTB, DSTN, and CAPZB. **B** Three genes involved in disulfidptosis, SLC7A11, ACTB, and MYL6, were differentially expressed between ACC and normal adrenal tissues. SLC7A11 expression was higher in ACC tissue than in normal adrenal tissue. **C** Two disulfidptosis-related genes, SLC7A11 and MYL6, were associated with prognosis in ACC patients. **D, E** Kaplan‒Meier survival curve of overall survival in ACC patients based on the expression of SLC7A11 and MYL6. **F** Expression levels of SLC7A11 in urothelial tumours and corresponding normal tissues were investigated using TCGA and GTEx databases. ACC, adrenocortical cancer; BLCA, bladder urothelial carcinoma; KIRC, kidney clear cell carcinoma; KICH, kidney chromophobe; KIRP, kidney papillary cell carcinoma; PRAD prostate adenocarcinoma; PCPG pheochromocytoma & paraganglioma; TGCT testicular germ cell tumour. *P value < 0.05, **P value < 0.01, ***P value < 0.001

Gene expression quantification data of transcriptome profiling (HTseq-FPKM) of 207 (including 79 tumours and 128 normal) samples were downloaded from the UCSC Xena platform. In addition, the clinical information of 77 patients with ACC was obtained from the TCGA database. Two ACC patients with incomplete clinical information were excluded from the clinically relevant study. Two sets of genes associated with disulfidptosis were derived from previous papers, and the two sets had 15 overlapping genes [15, 16] (Fig. 1A, Table S6). Three of these genes, SLC7A11, ACTB, and MYL6, were differentially expressed between ACC and normal adrenal tissues (| log2FoldChange) | > 1, adjusted P value < 0.05). SLC7A11, ACTB, and MYL6 were more highly expressed in the ACC samples (Fig. 1B, Figure S1A, B). In addition, SLC7A11 and MYL6 were significantly associated with overall survival (OS) in univariate Cox regression analysis (Fig. 1 C, *P* < 0.0001, *P* < 0.001). To further screen the hub genes, we analysed the effect of the SLC7A11 and MYL6 genes on overall survival (OS) using Kaplan‒Meier (K‒M) analysis. Increased expression of the SLC7A11 and MYL6 genes resulted in shorter overall survival (Fig. 1D-E, *P* < 0.001, *P* = 0.006). The TIMER database was used to verify the results of the survival analysis (Figure S1C, D). Based on its smallest *P* value in univariate Cox regression and K‒M analysis, SLC7A11 was chosen for further investigation. We further performed a pancancer analysis of SLC7A11 and found higher expression of SLC7A11 in some urology-related tumours (ACC, KIRC, KICH, KIRP, PRAD, and TGCT) than in corresponding normal tissues (Fig. 1F). Taken together, these results suggest that the disulfidptosis-related gene SLC7A11 is closely associated with ACC and warrants further investigation.

### Correlation between SLC7A11 and clinically relevant factors

**Fig. 2:**
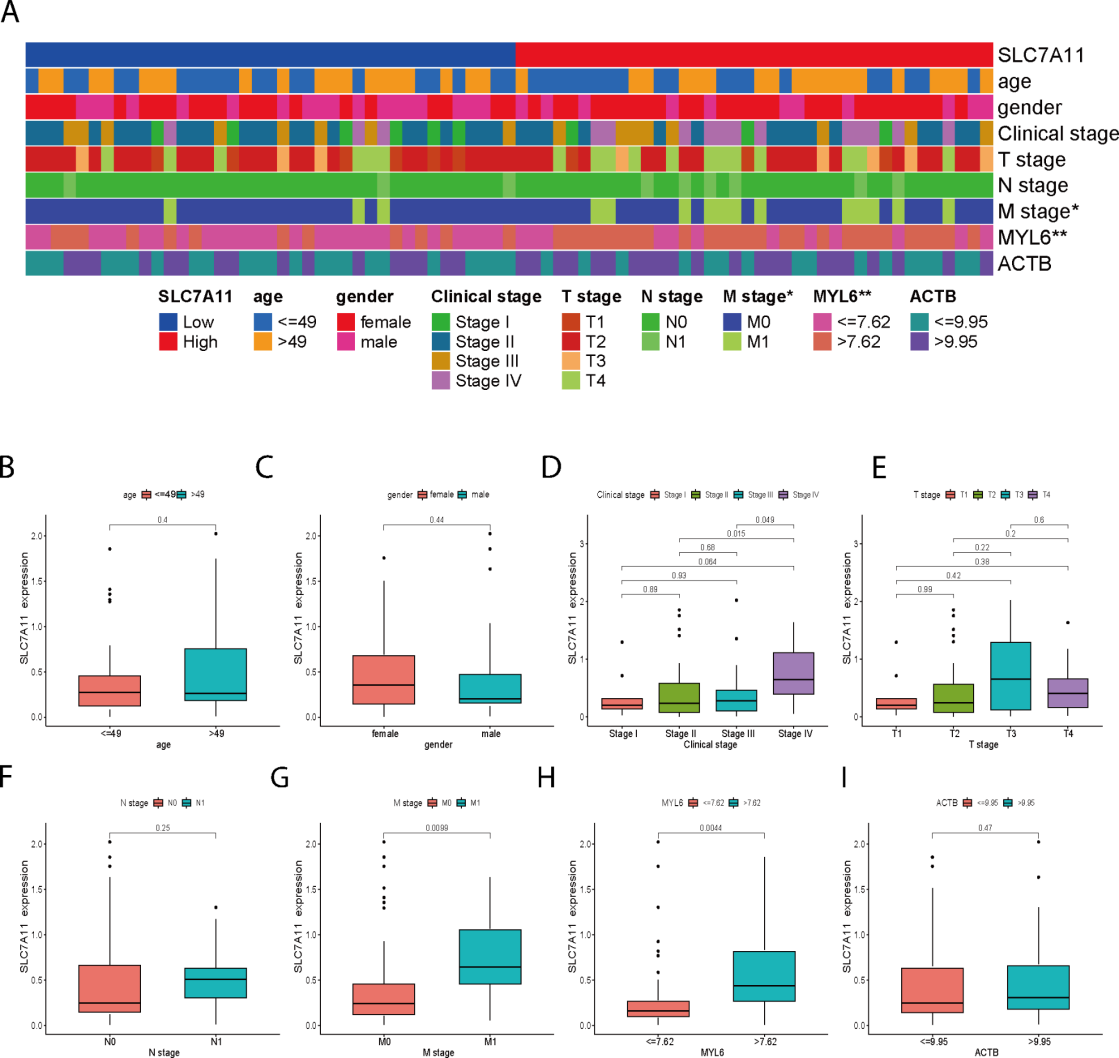
Correlation between SLC7A11 and clinically relevant factors, disulfidptosis-related genes, ACTB, and MYL6. **A** SLC7A11 expression was strongly associated with the M stage and MYL6 expression. **B-I** There was no significant correlation between age, sex, clinical grade, T or N stage, ACTB expression, and SLC7A11 expression. High expression of SLC7A11 was associated with metastasis and MYL6 expression in ACC. *P value < 0.05, **P value < 0.01, ***P value < 0.001

SLC7A11 expression in ACC patients was closely associated with M-stage and the expression of the disulfidptosis-related gene MYL6 (Fig. 2A). However, there was no statistical significance between SLC7A11 and some clinical factors, including age, sex, clinical grade, T-stage, N-stage, and ACTB (Fig. 2B-F, I). Myosin light chain 6 (MYL6), which plays an important role in regulating cell movement and assembling cytoskeleton structures, is bound by myosin heavy chain 14 (MYH14) and smooth muscle myosin [17, 18]. Overall, these results suggest that SLC7A11 is associated with ACC cell migration.

#### Construction and validation of a prognostic nomogram

**Fig. 3:**
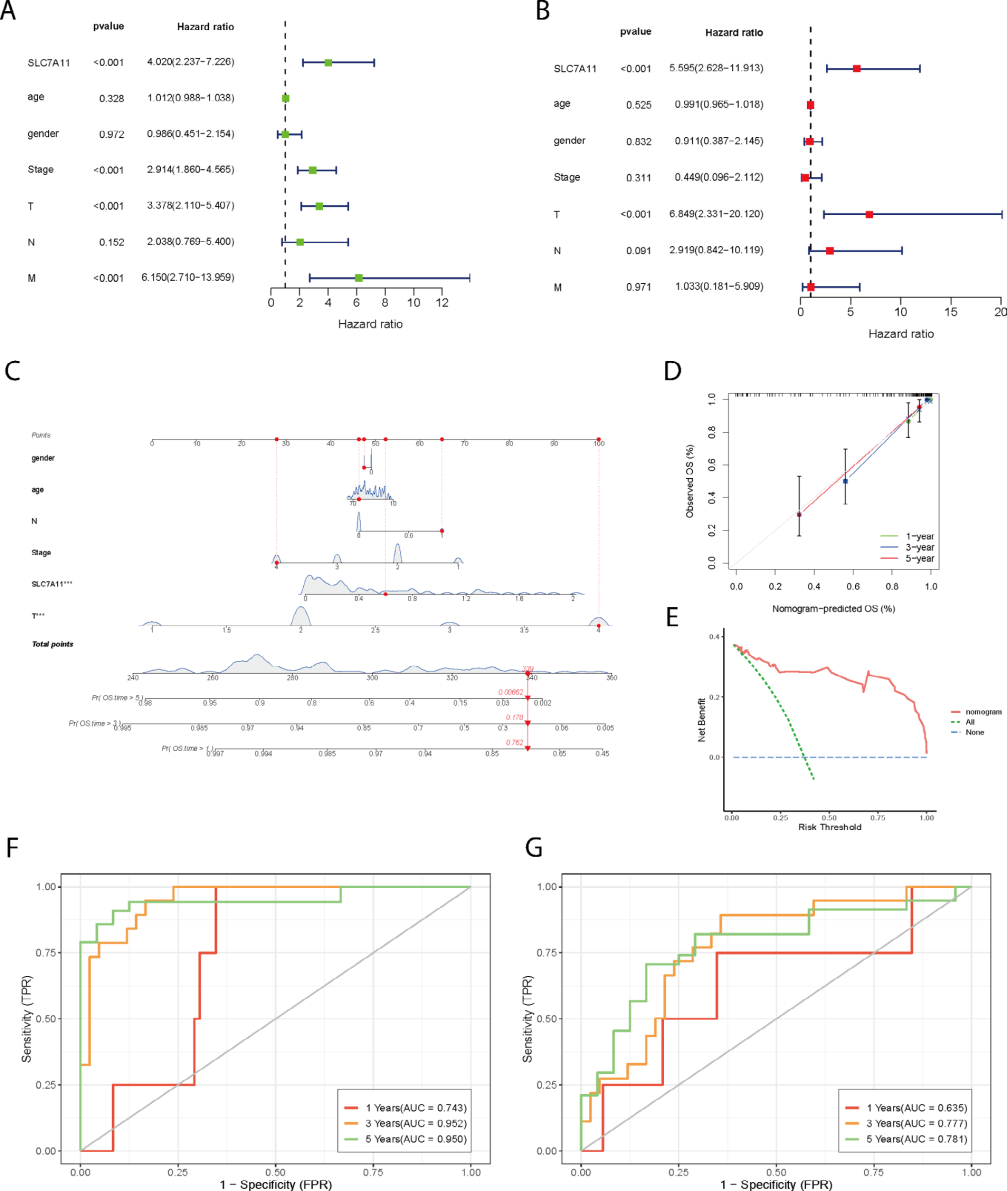
Construction and validation of a prognostic nomogram **A** Univariate Cox regression analysis suggested that SLC7A11 and clinically relevant factors (clinical stage, T stage, and M stage) are prognostic factors. **B** Multivariate Cox regression analysis suggested that SLC7A11 and T stage are independent clinical characteristics for overall survival prediction. **C** Nomogram for overall survival prediction, with sex, age, N-stage, clinical stage, T-stage, and the expression level of SLC7A11 applied as parameters. **D** Calibration curves of the nomogram for 1-, 3-, and 5-year overall survival prediction. **E** DCA evaluated the clinical practicability of the model and calculated the clinical benefit rate of the model. **F** Time-dependent receiver operating characteristic curves of the predictive value of the nomogram risk score for overall survival: 1-year AUC = 0.743, 3-year AUC = 0.952, and 5-year AUC = 0.950. **G** Time-dependent receiver operating characteristic curves of the predictive value of SLC7A11 for overall survival: 1-year AUC = 0.635, 3-year AUC = 0.777, and 5-year AUC = 0.781

Univariate and multivariate Cox regression analyses showed that SLC7A11 and T stage were independent prognostic factors for OS, with HRs of 5.595 (95% CI, 2.628–11.913) and 6.849 (95% CI, 2.331–20.120), respectively (Fig. 3A-B). A nomogram was established for 1-, 3-, and 5-year OS prediction in ACC patients based on the TCGA database (Fig. 3 C). The expression level of SLC7A11, age, sex, T stage, N stage, and clinical stage were eventually applied as parameters. The M-stage was excluded due to statistical uncertainty and imbalanced distribution. The calibration curves of the 1-year, 3-year, and 5-year OS rates fit well with the ideal model (Fig. 3D). The DCA curves provided insight into the range of predicted risks, suggesting that the model provides high clinical value for patients (Fig. 3E). The area under the curve (AUC) for the SLC7A11 prognostic model was 0.743 for the 1-year ROC curve, 0.952 for the 3-year ROC curve, and 0.950 for the 5-year ROC curve of the discovery TCGA cohort (Fig. 3F). The AUCs of the SLC7A11 expression groups for OS at 1, 3, and 5 years were 0.635, 0.777 and 0.781, respectively (Fig. 3G). In summary, these results indicate that SLC7A11 is an independent prognostic factor in ACC patients, and the nomogram we constructed is highly predictive.

### Screening for SLC7A11-related modules and genes in ACC

**Fig. 4:**
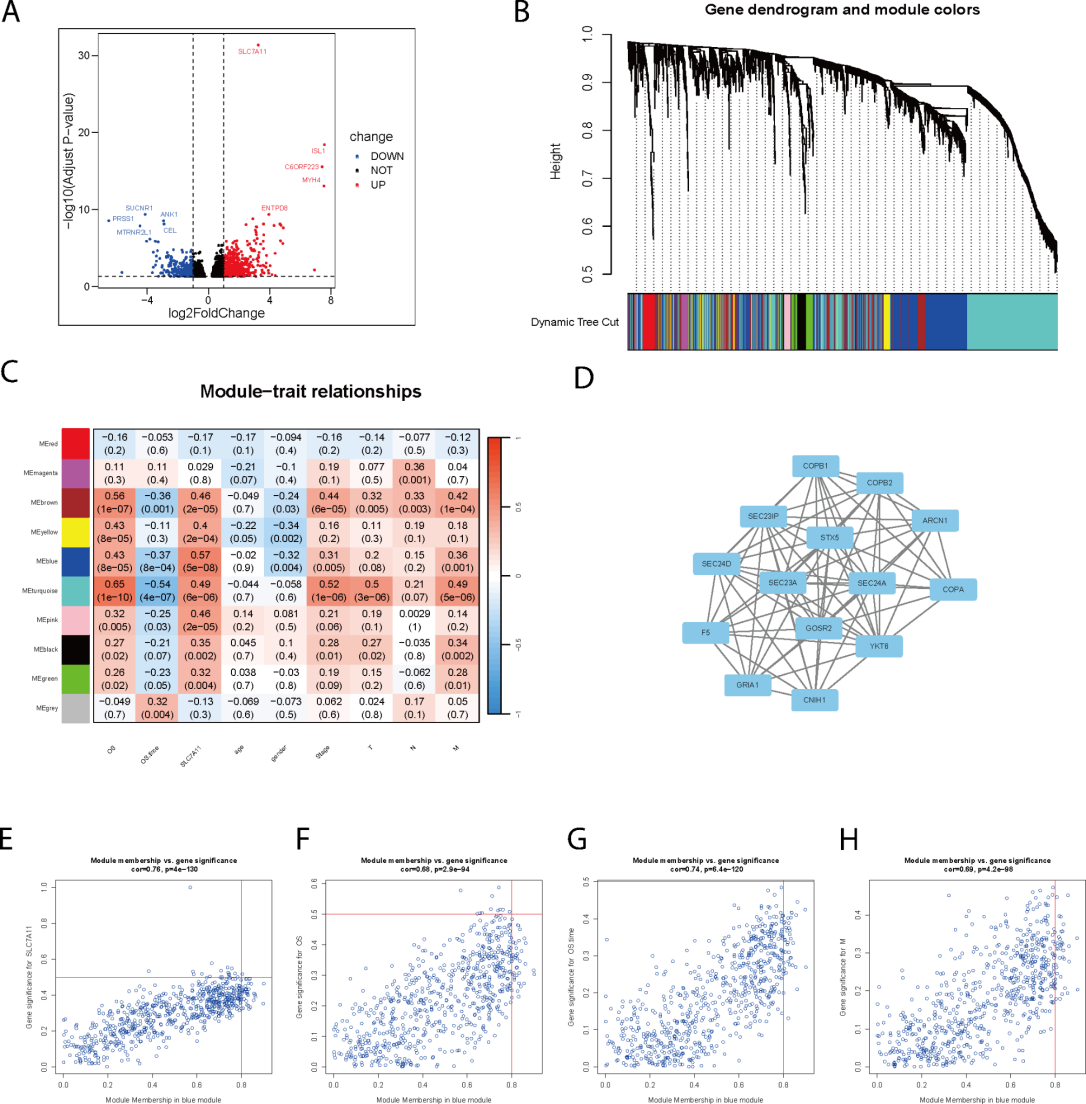
Screening for SLC7A11-related modules and genes in the ACC **A** Volcano map revealing the differences in gene expression between the SLC7A11 upregulated and SLC7A11 downregulated expression groups. **B** Cluster dendrogram of ACC patients. Each coloured row represents a colour-coded module that contains a group of highly connected genes. A total of 10 modules were identified. **C** The correlation heatmap between the template and clinically relevant factors (OS, OS.time, SLC7A11, age, sex, clinical stage, T stage, N stage, and M stage). Each cell contains the corresponding correlation and *p* value. **D** Hub gene network in blue template. **E-H** Scatter plot of the blue module

A total of 2057 differentially expressed genes (DEGs) were identified between the high and low SLC7A11 expression groups (Fig. 4A). To analyse the genes associated with SLC7A11 and further investigate the potential counteractivity of SLC7A11 in ACC and disulfidptosis development, we performed a weighted gene coexpression network analysis. All the DEGs were grouped into 10 modules according to average linkage hierarchical clustering (Fig. 4B,C, Figure S2A,B). There is a highly significant correlation between GS (Gene Significance) and MM (Module Membership) in this module (Fig. 4E-H). We identified hub genes in the blue module (Fig. 4D) and observed a significant correlation between these genes and SLC7A11 (P = 7.8e-9, R = 0.6), as well as genes related to disulfidptosis (P = 0, R = 0.8) in ACC (Fig S2C,D). Taken together, these results further demonstrate that SLC7A11 and its associated genes are strongly associated with disulfidptosis, ACC cell migration and prognosis in ACC patients.

#### The potential mechanism of SLC7A11 in ACC and disulfidptosis development

**Fig. 5:**
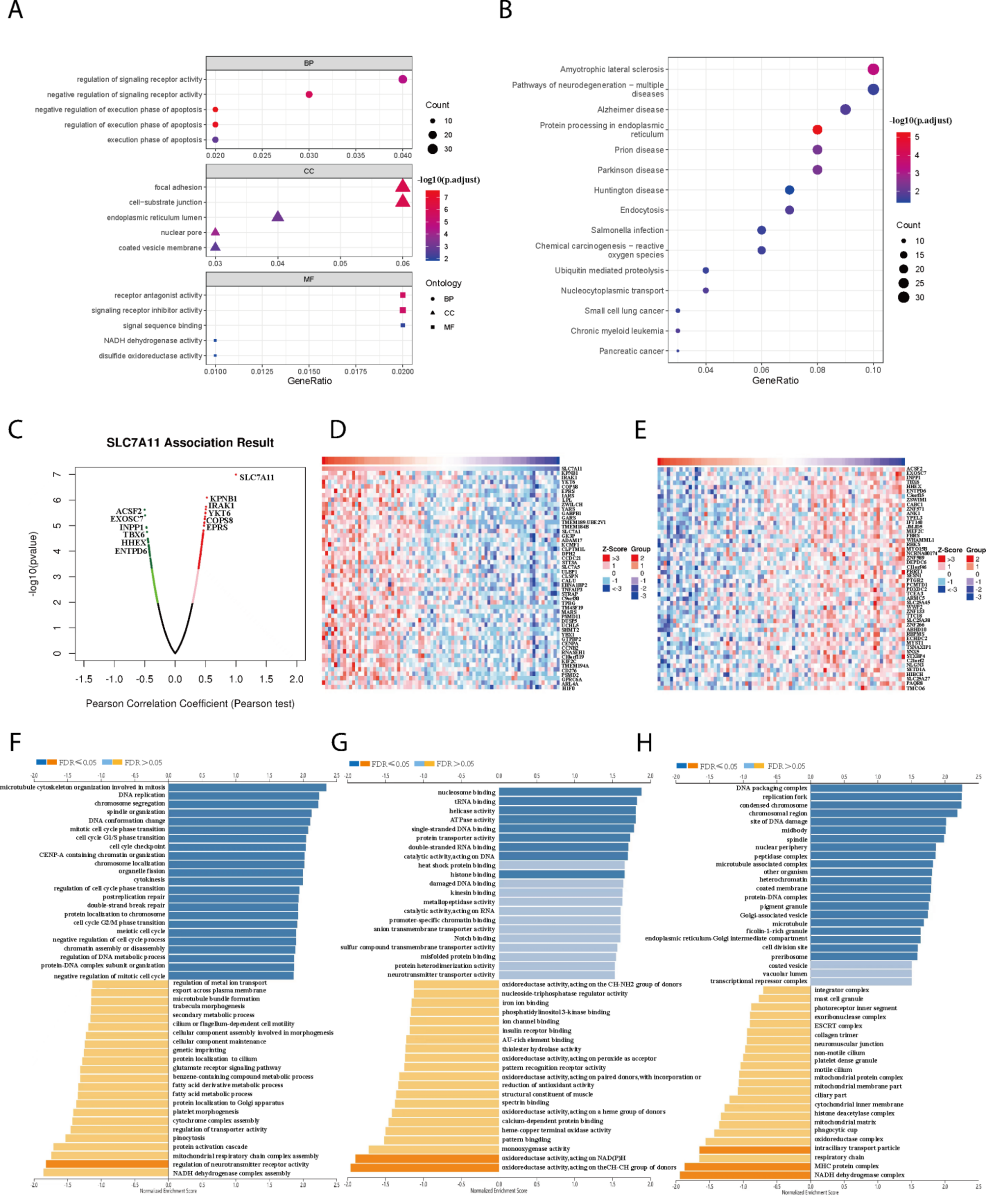
The potential mechanism of SLC7A11 in ACC and disulfidptosis development **A, B** GO and KEGG enrichment analysis of 686 blue module genes. **C** The volcano plot shows all genes related to SLC7A11,and we have highlighted 12 prominent genes among them. **D, E** Top 50 SLC7A11-related genes with positive/negative correlations. **F, G, H** GO enrichment analysis (BP, CC, MF) of these genes in the LinkedOmics database

According to the GO enrichment analysis of the 686 genes in the blue module, SLC7A11 showed associations with key biological processes such as the execution phase of apoptosis, regulation of apoptosis (BP category). In the cellular component (CC) category, SLC7A11 was linked to focal adhesion, endoplasmic reticulum lumen. Furthermore, in the molecular function (MF) category, it was associated with disulfide oxidoreductase activity and NADH dehydrogenase activity (Fig. 5A). KEGG enrichment analysis revealed its involvement in chemical carcinogenesis-reactive oxygen, and protein processing in the endoplasmic reticulum (Fig. 5B). Furthermore, the results of the Gene Set Enrichment Analysis (GSEA) revealed a significant association between these genes and “Oxidative phosphorylation” and “Chemical carcinogenesis - reactive oxygen species” pathways in ACC (Table S7). Another module we constructed to gain insight into the SLC7A11 coexpression genes in ACC was the LinkFinder module via the LinkedOmics database (Fig. 5 C). Heatmaps were used to show the top 50 SLC7A11-related genes with positive/negative correlations (Fig. 5D-E). The GO enrichment analysis performed in the LinkedOmics database suggested that SLC7A11 may be associated with NADH dehydrogenase complex assembly, and oxidoreductase activity, acting on NAD(P)H (Fig. 5F-G). These findings collectively support the strong association of SLC7A11 and its associated genes with disulfide bond formation, disulfide metabolism, and the occurrence of disulfidptosis. SLC7A11, which is highly expressed in ACC, sets the stage for disulfidptosis.

#### Correlation between SLC7A11 and immune infiltration

**Fig. 6:**
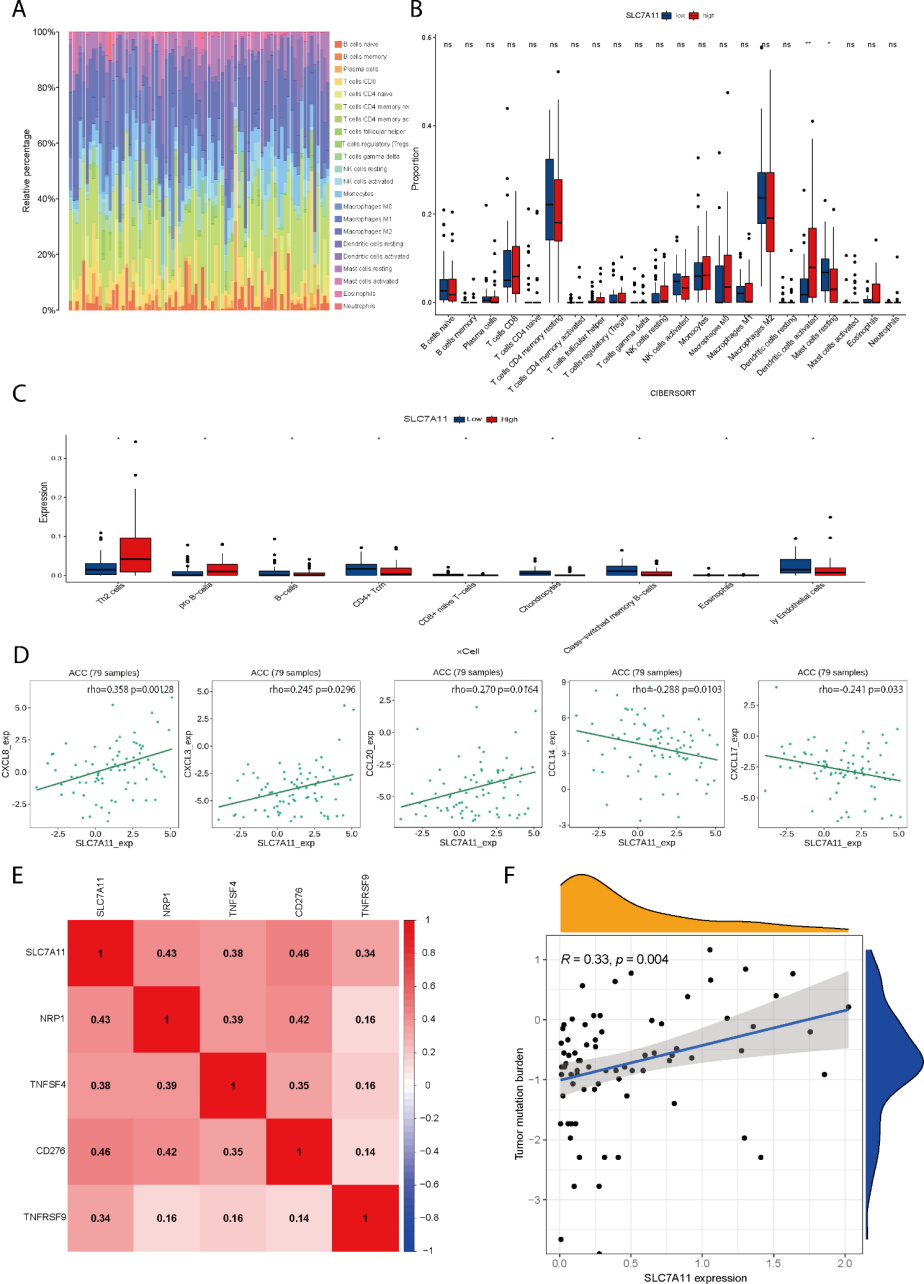
Correlation between SLC7A11 and immune infiltration **A** Overall infiltration of 22 immune cells in ACC. **B** The CIBERSORT algorithm revealed that the infiltration levels of activated dendritic cells were increased in the high SLC7A11 expression group, while the infiltration levels of resting mast cells were decreased in the same group. **C** The Xcell algorithm revealed that the infiltration levels of Th2 cells and pro B cells were increased in the high SLC7A11 expression group, while the infiltration levels of B cells, cd4 + Tcms, CD8 + naive T cells, chondrocytes, class-switched memory B cells, eosinophils and ly endothelial cells were decreased in the same group. **D** SLC7A11 expression was positively correlated with the immune chemokines CXCL8, CXCL3, and CCL20 and negatively correlated with the immune chemokines CCL14 and CXCL17. **E** SLC7A11 expression was positively correlated with immune checkpoint genes (NRP1, TNFSF4, CD276, TNFRSF9). **F** SLC7A11 expression was positively correlated with tumour mutation burden. *P value < 0.05, **P value < 0.01, ***P value < 0.001

The overall infiltration of 22 immune cells in ACC was determined based on “CIBERSORT”, with resting memory CD4 T cells and macrophages accounting for a larger proportion (Fig. 6A). Differential analysis of immune cell infiltration levels in the high and low SLC7A11 expression groups was performed based on the “CIBERSORT” algorithm. The infiltration levels of resting dendritic cells were positively correlated with SLC7A11, while the infiltration levels of mast cells were negatively correlated with SLC7A11 (Fig. 6B, Figure S3A). Differences in immune cell infiltration based on the “X cell” algorithm revealed that the infiltration levels of Th2 cells and pro B cells were positively correlated with SLC7A11, while the infiltration levels of B cells, cd4 + Tcm cells, CD8 + naive T cells, chondrocytes, class-switched memory B cells, eosinophils and ly endothelial cells were negatively correlated with SLC7A11 (Fig. 6 C, Fig S3D). We also investigated the correlations between the expression of SLC7A11 and TME scores and immune cell infiltration (Figure S3B). Then, we further investigated the association between SLC7A11 and immune chemokines via the TISIDB database. The results showed that SLC7A11 had a positive relationship with the chemokines CXCL8 (rho = 0.358, *P* = 0.00128), CXCL3 (rho = 0.245, *P* = 0.0296), and CCL20 (rho = 0.27, *P* = 0.0164) and an inverse relationship with the chemokines CCL14 (rho=-0.288, *P* = 0.0103) and CXCL17 (rho=-0.241, *P* = 0.033) (Fig. 6D). Thus, SLC7A11 expression is strongly associated with the tumour immune microenvironment (Figure S3C). We then investigated the associations between SLC7A11 and 47 immune checkpoint-related genes. SLC7A11 was significantly associated with 4 immune checkpoint genes (*P* < 0.05). SLC7A11 was positively associated with CD276 (cor = 0.463, *P* < 0.00001), NRP1 (cor = 0.434, *P* < 0.0001), TNFSF4 (cor = 0.38, *P* < 0.001), and TNFRSF9 (COR = 0.34, *P* < 0.05) (Fig. 6E). We also performed a correlation analysis for SLC7A11 and tumour mutation burden (TMB), which showed a positive correlation between SLC7A11 and TMB (cor = 0.33, *P* = 0.004) (Fig. 6F). Overall, these results suggest that the dysregulation of the tumour immune microenvironment in ACC was strongly associated with SLC7A11^high^. Furthermore, ACC patients with SLC7A11 overexpression may have a better response to immunotherapy.

#### Correlation between SLC7A11 and drug sensitivity in ACC

**Fig. 7:**
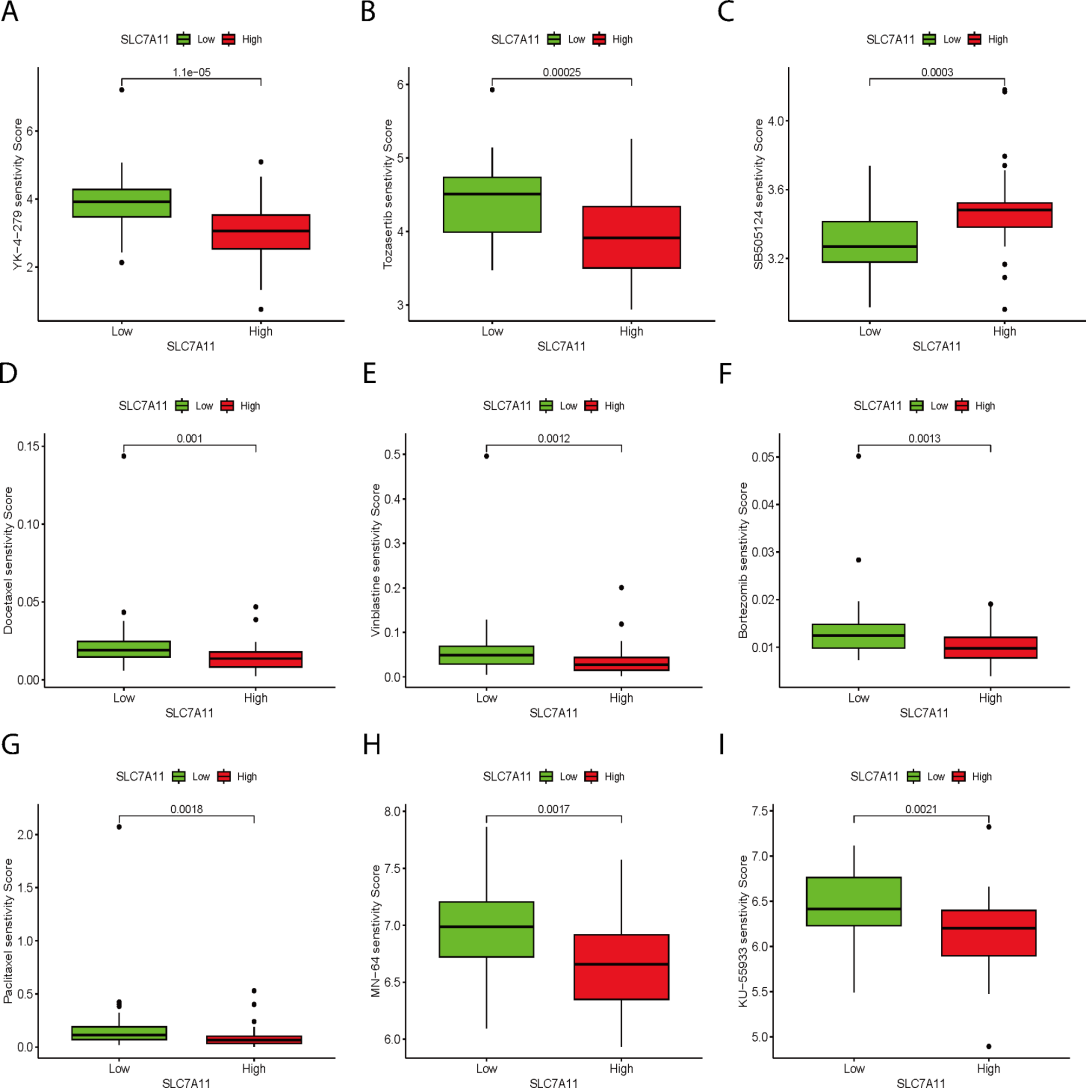
Correlation between SLC7A11 and drug sensitivity in ACC **A-I** SLC7A11 expression was negatively correlated with sensitivity to multiple antitumour drugs in ACC patients. **A** YK-4-279, **B** tozasertib, **C** SB505124, **D** docetaxel, **E** vinblastine, **F** bortezomib, **G** paclitaxel, **H** MN-64, and **I** KU-55,933

Differential analysis of the drug senstivity score of 198 common chemotherapy drugs between the high and low SLC7A11 expression groups was conducted. We identified 55 statistically significant chemotherapy drugs, most of which had significantly lower drug senstivity score in the high SLC7A11 expression group, such as YK-4-279, tozasertib, docetaxel, vinblastine, bortezomib, paclitaxel, MN-64, and KU-55,933. Only a few drugs exhibited higher senstivity score in the group with high SLC7A11 expression, including SB505124 (Fig. 7A-I; Figure S4A-I). In conclusion, these results suggest a close association between the expression level of SLC7A11 and the sensitivity of ACC patients to anti-tumor drugs, implying a potentially significant role of SLC7A11 in the treatment of ACC.

### The expression validation of SLC7A11 in ACC

**Fig. 8:**
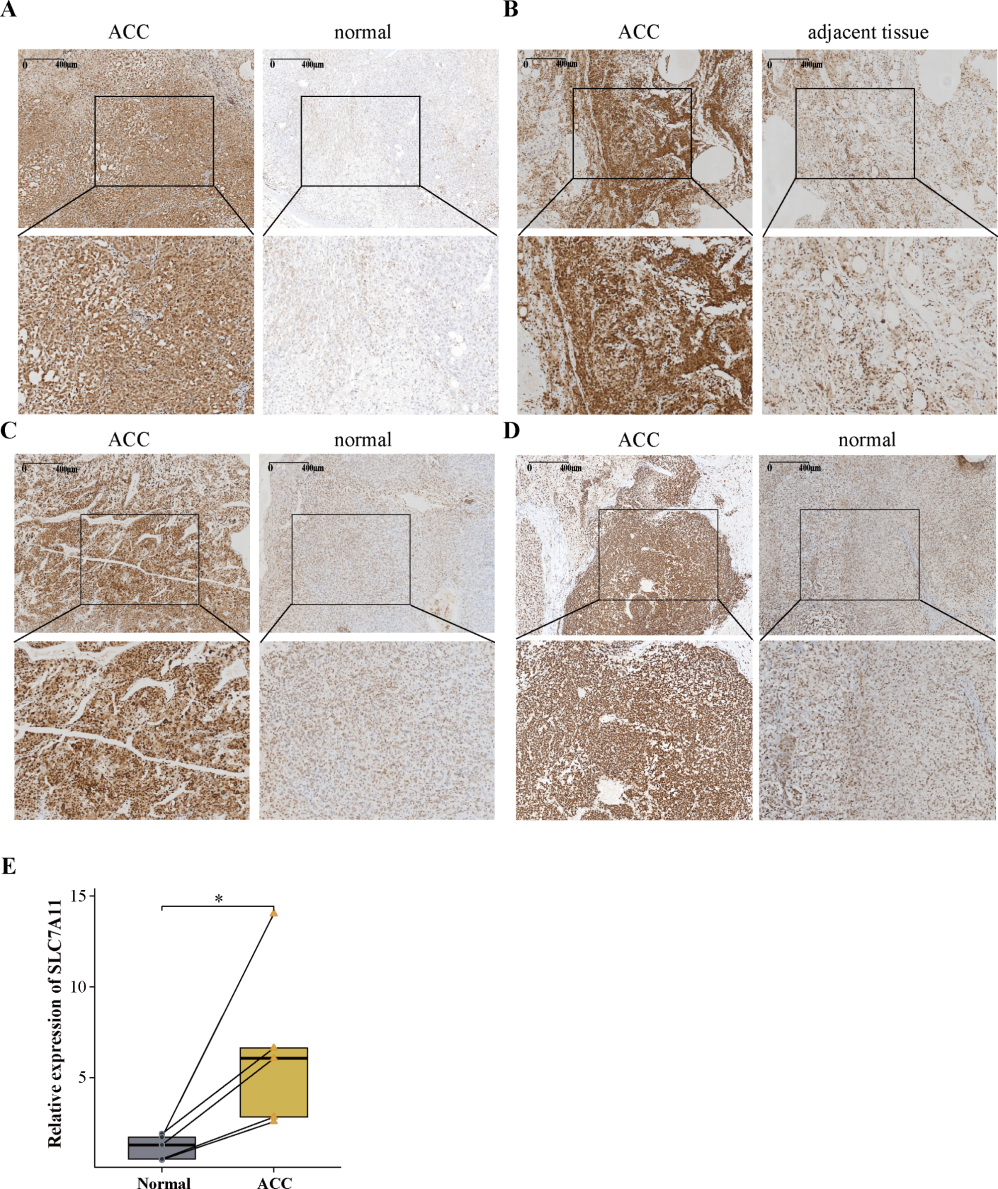
Histopathology and immunohistochemical staining **A, B, C, D** Histopathology and immunohistochemical staining of SLC7A11 expression in ACC tissue, ACC-adjacent tissue and normal adrenal tissue. **E**, RT-qPCR detection of SLC7A11 expression levels in ACC and normal adrenal tissue

Histopathology and immunohistochemical staining revealed that SLC7A11 expression was significantly increased in ACC tissue compared to ACC-adjacent tissue and normal adrenal tissue (Fig. 8A-D, Figure S5A-D). The results of RT-qPCR clearly demonstrate a significantly higher expression of SLC7A11 in ACC compared to normal adrenal gland samples (p < 0.01) (Fig. 8E, Table S8). Taken together, these results validate our analysis of SLC7A11 expression.

## Discussion

ACC is a malignant tumour with a poor prognosis and is usually associated with hormone secretion, such as that for glucocorticoids and androgens [19, 20]. Although significant advances have been made in immunotherapy and targeted therapies for ACC, effective therapies for ACC are still lacking in clinical practice [2]. Recent studies have revealed that killing cancer cells by inducing programmed cell death processes, such as disulfidptosis, ferroptosis, and apoptosis, can be a promising treatment method.

We obtained gene sets related to disulfidptosis from previous related studies and screened them for SLC7A11 [15, 16], the gene most closely associated with ACC. SLC7A11 is a cysteine transporter that exports intracellular glutamate and imports extracellular cystine at a 1:1 ratio [21]. Cysteine uptake in cells is mainly dependent on System Xc-. This transport system consists of two subunits, the light-chain transport subunit SLC7A11 and the heavy-chain regulatory subunit SLC3A2; SLC7A11 is a 12-pass transmembrane protein that primarily mediates cysteine/glutamate reverse transport activity [22]. SLC3A2 is primarily responsible for maintaining the protein stability and membrane localization of SLC7A11 as a chaperone. In other words, SLC7A11 is essential for regulating cysteine transport. Moreover, SLC7A11 is strongly associated with the development of multiple malignancies and often predicts poor prognosis. We found that SLC7A11 is differentially expressed in most urinary system-related tumours and is often highly expressed in tumour tissues. In ACC, SLC7A11^high^ is an independent prognostic factor, and the nomogram based on SLC7A11 and other clinically relevant factors can accurately predict prognostic relevance in ACC patients.

SLC7A11^high^ promotes cysteine transport in cancer cells and increases intracellular cysteine and extracellular glutamate accumulation. Cystine is converted to cysteine, a rate-limiting precursor for glutathione (GSH), in the cytosol through an NADPH-consuming reduction reaction [21]. GSH is a very important reducing agent in cells and is essential for maintaining the reducing environment in cells. Cancer cells have more reactive oxygen species (ROS) than normal cells. ROS are normally used to stimulate tumorigenesis and progression but can induce cell death when they exceed safe limits [12, 23, 24]. Increased intracellular GSH alleviates this problem well, allowing cancer cells to live in a comfortable environment. In other words, SLC7A11^high^ in cancer cells contributes to the development of cancer cells.

In addition, previous studies indicated that GSH may be associated with tumour drug resistance and that elevated GSH may increase drug resistance in cancer cells. Okuno et al. [25] revealed that SLC7A11-mediated upregulation of GSH promotes cisplatin resistance in ovarian cancer cells. Lo et al. [26] found that GSH upregulation was associated with increased resistance to gemcitabine in pancreatic cancer cells. Our results on drug sensitivity analysis demonstrate a strong correlation between the expression of the SLC7A11 gene and the sensitivity of ACC patients to anti-tumor drugs. ACC patients with high expression of SLC7A11 exhibit increased sensitivity to certain anti-tumor drugs (YK-4-279, tozasertib, docetaxel, vinblastine and so on), suggesting an important role for SLC7A11 in the treatment of ACC patients.

In addition, previous studies have shown that SLC7A11-mediated extracellular glutamate accumulation not only serves as a raw material for cancer cell growth but also plays an essential role in tumour migration and invasion [27, 28]. Susan et al. [29] found that system Xc–released glutamate acts on Ca^2+^-permeable α-amino-3-hydroxy-5-methylisoxazole-4-propionic acid receptors (AMPA-R) in glioma cells, inducing intracellular Ca^2+^ oscillations that affect tumour cell migration and invasion ability. The researchers found that glutamate transferred by system Xc- to the extracellular space drives epithelial morphology destruction and promotes lumen filling and basement membrane disruption, the key characteristics of the invasive phenotype of cancer cells [30]. Similarly, when we performed a clinically relevant analysis of SLC7A11, we found that SLC7A11 was associated with M-stage and MYL6. The M1 stage was more prevalent in ACC patients with SLC7A11^high^, and MYL6 played an important role in cancer cell migration. This suggests that SLC7A11 may contribute to distant tumour migration. Furthermore, when we performed weighted gene coexpression analysis using the “WGCNA” R package, we found that the blue template genes most closely associated with SLC7A11 were strongly associated with M-stage. Further enrichment analysis of these genes revealed a strong association with cellular component focal adhesions. Focal adhesions are made up of more than 150 different proteins that bind the cytoskeleton to the extracellular matrix and are involved in the migration, proliferation, and differentiation of cancer cells [31–33]. Dynamic turnover of focal adhesions is key to cell migration [34]. Thus, in ACC, SLC7A11^high^ is strongly associated with the invasion and migration of cancer cells.

In addition, glutamate regulates the immune microenvironment of cancer cells by binding to glutamate receptors on cancer cells [35]. Long et al. [36] found that glutamate promotes the proliferation, activation, and immune suppression of Tregs in gliomas. In our study, we also found that SLC7A11 expression is strongly associated with the dysregulation of immune cell infiltration in ACC patients. In ACC patients with SLC7A11^high^, the number of Th2 cells and pro B cells was increased, but the number of B cells, cd4 + Tcms, CD8 + naive T cells, chondrocytes, class-switched memory B cells, eosinophils and ly endothelial cells was decreased.

Furthermore, SLC7A11 was significantly associated with 4 immune checkpoint genes, CD276, NRP1, TNFSF4, and TNFRSF9. Through the TISIDB database, we also learned that SLC7A11 expression in ACC is positively correlated with the immune chemokines CXCL8, CXCL3, and CCL20 and negatively correlated with the immune chemokines CCL14 and CXCL17. CXCL8, also known as interleukin-8 (IL8), and its associated pathway CXCL8-CXCR1/2 play an important role in tumour proliferation, invasion, and migration [37, 38]. In Ras-driven cancers, CXCL8 attracts tumour-associated neutrophils (TANs) to the tumour immune microenvironment, and TANs secrete arginase 1 to favour immune suppression [39, 40]. The CXCL8-CXCR1/2 pathways also play a confirmed role in resistance to chemotherapy in breast, prostate, and colorectal cancers [37]. Previous studies have also shown that the immune chemokines CXCL3 and CCL20 also play an important role in tumour growth, invasion, and migration [41, 42]. Thus, the immune chemokines CXCL8, CXCL3, and CCL20, which are positively associated with SLC7A11, have SLC7A11-like functions in the development of tumours and provide evidence of association at the tumour immune microenvironment level for poor prognosis in ACC patients with SLC7A11^high^.

In summary, SLC7A11 can influence tumour progression, drug sensitivity, immune infiltration, and the ability to migrate and invade distant sites by modulating cysteine/glutamate transportation in the ACC. It has the potential to be an essential molecular marker for assessing prognosis in ACC patients. Given the range of roles that SLC7A11 plays in tumour development, the development of oncology therapies targeting SLC7A11 is clinically significant.

Current applications of SLC7A11 in the treatment of tumours can be broadly classified into two categories: direct targeting of SLC7A11 transporter activity, which inhibits tumour progression by inducing intracellular ROS accumulation and ferroptosis, and targeting metabolic vulnerabilities exposed by SLC7A11-overexpressing cancer cells, such as glucose or glutamine dependency [21]. SLC7A11^high^ in cancer cells was associated with increased consumption of NADPH and glutamate. Statistically, approximately 30–50% of glutamate is exported by SLC7A11 in exchange for cysteine [43]. The deficiency of NADPH and glutamate is compensated by glucose and glutamine metabolism. Thus, SLC7A11^high^ cancer cells are highly dependent on glucose and glutamine, which is thought to be the metabolic vulnerability of exposed cancer cells [44].

Recent studies have found that when glucose is scarce, SLC7A11^high^ cancer cells significantly reduce NADPH, which is produced by the glucone-gluconate pentachlorophenate pathway. This leads to a substantial buildup of disulfides in the cells, which in turn triggers disulfide stress and the collapse of actin cytoskeleton proteins that ultimately leads to rapid cell death. This form of death differs from ferroptosis and apoptosis and is called disulfidptosis. Cysteine starvation is known to lead to ferroptosis in cancer cells [45]. Previous studies have found that cancer cells with SLC7A11^high^ have higher levels of cysteine accumulation [46]. When cysteine starvation occurs, cancer cells do not experience significant ferroptosis but prevent cell death caused by glucose deficiency [47]. In other words, in cancer cells with SLC7A11^high^, glucose starvation may carry more cytotoxicity than cysteine deficiency does [21]. This also suggests that targeting SLC7A11^high^ tumours exposes metabolic vulnerability and induces disulfidptosis in cancer cells as a potential therapeutic approach. This seems to be a treatment that works on a wide range of tumours. However, in previous experiments, researchers found that glucose transporter inhibitors were not effective in inducing disulfidptosis in some cancer cells [21]. This may be because some cancer cells do not have the conditions for disulfidptosis or because of their strong resistance to glucose starvation, the mechanisms of which need to be further investigated.

Disulfidptosis and treatments targeting metabolic vulnerability have not been reported in ACC studies, and our analysis of a public database revealed that ACC has the underlying conditions for disulfidptosis. Thus, treatments targeting metabolic vulnerability are promising for ACC treatment.

We performed differential analysis using the TCGA and GTEx databases and found that SLC7A11 was significantly more highly expressed in ACC than in normal adrenal tissue [48]. Increased expression of SLC7A11 mediates increased intracellular cysteine and NADPH depletion, laying the groundwork for disulfidptosis in ACC.

Studies have shown that glucose is usually metabolized in two major ways: glycolysis and the pentose phosphate pathway [49]. The pentose phosphate pathway is a ubiquitous glucose metabolism pathway in plants, animals, and microorganisms. The pentose phosphate pathway varies from tissue to tissue and is less common in skeletal muscle tissue and more common in tissues with high lipid content, such as the adrenal gland, breast, and adipose tissue [50, 51]. In addition, the pentose phosphate pathway is an important source of NADPH, which is used to synthesize steroids produced in the adrenal cortex [50, 52]. Thus, the adrenal gland is highly dependent on NADPH, suggesting that blocking glucose uptake may be effective in inducing disulfidptosis in ACC cells.

The enrichment analysis of SLC7A11 and its closely related genes indicated that these genes are closely associated with endoplasmic reticulum lumen, focal adhesion, and disulfide oxidoreductase activity in ACC. The endoplasmic reticulum is a reticular organelle that regulates the folding and posttranslational maturation of most membrane proteins and secretory proteins [53]. Proteins synthesized in the endoplasmic reticulum, often require a stable three-dimensional structure through the production of disulfide bonds, which are the basis of protein biological functions [54]. With the formation of disulfide bonds, H_2_O_2_, a byproduct, is produced in the endoplasmic reticulum, and ROS levels in the endoplasmic reticulum are subsequently upregulated [54]. Under normal conditions, the reductive environment in the cytoplasm and endoplasmic reticulum prevents the production of disulfide bonds by cytoplasmic proteins [11], suggesting that ROS produced during protein folding and maturation in the endoplasmic reticulum contribute to disulfide bonds and disulfide production. In conclusion, the endoplasmic reticulum is an important site for the formation of disulfide bonds in cells and is closely associated with the occurrence of disulfidptosis.

The production of disulfide bonds mainly depends on the protein disulfide isomerase (PDI) family and other oxidoreductases [54]. In our enrichment analysis of SLC7A11 and its closely related genes, we found that these genes appear to regulate the activity of disulfide oxidoreductase, which regulates disulfide bonds and disulfide synthesis in ACC cells [55]. We also noted that these genes are closely associated with oxidoreductase activity, acting on NADP (H) and NADP dehydrogenase activity when validated using the LinkedOmics database. Thus, SLC7A11 is highly expressed in ACC cells, which not only influences the formation of disulfide bonds in cells but also regulates glucose and NADPH metabolism, leading to the potential for disulfidptosis in ACC cells.

If attempting to target the metabolic vulnerability displayed by ACC cells with high expression of SLC7A11, such as inhibiting glucose transporters which would impede glucose uptake and subsequently lead to a significant reduction in NADPH production through the phosphogluconate pathway, it is very likely to induce the accumulation of intracellular disulfide bonds and disulfides, thereby triggering disulfidptosis. Therefore, when employing this approach for ACC treatment, the induction of disulfidptosis in ACC cells can potentially improve patient prognosis. In addition, previous studies have shown that glucose transporter inhibitors can inhibit the development of multiple tumours. Additionally, they can overcome tumour cell resistance to chemotherapeutics, radiotherapy, and immunotherapies and enhance the anticancer efficacy of antitumour agents [56–58]. In summary, targeting metabolic vulnerability in cancer is fraught with limitless possibilities in the era of precision oncology, and targeting metabolic vulnerability to induce disulfidptosis in ACC cells holds promise for the treatment of ACC.

Although our study demonstrates the significance of SLC7A11 for the prognostic prediction of ACC and the occurrence of disulfidptosis, it still has limitations. First, gene expression and clinical information were derived from public databases, and our findings need to be validated through other clinical data we collected. Second, the specific mechanism of SLC7A11 and disulfidptosis in ACC needs to be further investigated.

In summary, this study demonstrates that the highly expressed disulfidptosis-related gene SLC7A11 influences glucose and NADPH metabolism and regulates disulfide bonds and disulfide formation in ACC. These results suggest that ACC cells with SLC7A11^high^ have the potential for disulfidptosis and that targeting their metabolic vulnerability to induce disulfidptosis has the potential to improve overall patient survival.

## Conclusion

In summary, our study found that high expression of disulfidptosis-related SLC7A11 was associated with migration, invasion, drug sensitivity, immune infiltration disorders, and poor prognosis in ACC, and targeting its metabolic vulnerability to induce disulfidptosis has the potential to improve overall patient survival.

### Electronic supplementary material

Below is the link to the electronic supplementary material.

**Additional file 1: Figure S1 Expression of disulfidptosis-related genes and their effect on the prognosis of ACC patients A, B** Expression of the genes MYL6 and ACTB in ACC and normal adrenal tissues. **C, D** Kaplan‒Meier survival analysis of SLC7A11 and MYL6 in the TIMER database.
**Additional file 2: Figure S2 Sample dendrogram and trait heatmap A** ACC sample dendrogram and trait heatmap.B Calculation of the scale-free fit index of various soft-thresholding powers (β) and analysis of the mean connectivity of various soft-thresholding powers (β). C,D The relationship between hub genes in the blue module and SLC7A11, as well as genes related to disulfidptosis.
**Additional file 3 : Figure S3 Relationships between SLC7A11 expression and immune cell infiltration in ACC A, B** Relationships between SLC7A11 expression and immune cell infiltration. **C** Relationships between SLC7A11 expression and chemokines. D The Xcell algorithm revealed that the infiltration levels of some immune cells in ACC patients.
**Additional file 4: Figure S4** **Relationship between SLC7A11 expression and drug sensitivity in ACC A-I** SLC7A11 expression correlates with the sensitivity of anticancer drugs in ACC patients. Additional file 5: figure S5 haematoxylin-eosin staining of tissue samples A-D HE staining in four ACC samples. Additional file 6: **Table S1** The TCGA identifier numbers of ACC samples; **Table S2** The clinical information for the 77 ACC patients; **Table S3** Two sets of genes associated with disulfidptosis; **Table S4** The expression profiles of SLC7A11 in urogenital system-related tumors; **Table S5** The 47 immune checkpoint-related genes; **Table S6** The 15 overlapping genes of the two sets of genes associated with disulfidptosis; **Table S7** The results of the Gene Set Enrichment Analysis (GSEA);**Table S8** The q-pcr raw data.

## Data Availability

The publicly available datasets used in this study can be found in the Materials and Methods.

## List of abbreviations

ACC: Adrenocortical carcinoma
GO: Gene Ontology
KEGG: Kyoto Encyclopedia of Genes and Genomes
NADPH: Nicotinamide adenine dinucleotide phosphate
SLC7A11: Solute carrier family 7 member 11
TCGA: The Cancer Genome Atlas
GTEx: Genotype–Tissue Expression Project
GSH: Glutathione
ROS: Reactive oxygen species
ER: Endoplasmic reticulum
