# Supplementary material for: Exploring the role of the disulfidptosis-related gene SLC7A11 in adrenocortical carcinoma: implications for prognosis, immune infiltration, and therapeutic strategies
Source: Cancer Cell Int. 2023 Nov 2;23:259. doi: 10.1186/s12935-023-03091-6 (PMC10623781; doi:10.1186/s12935-023-03091-6)
Supplement: Supplementary file 1 — Additional file 1: Figure S1 Expression of disulfidptosis-related genes and their effect on the prognosis of ACC patients A, B Expression of the genes MYL6 and ACTB in ACC and normal adrenal tissues. C, D Kaplan‒Meier survival analysis of SLC7A11 and MYL6 in the TIMER database. Additional file 2: Figure S2 Sample dendrogram and trait heatmap A ACC sample dendrogram and trait heatmap.B Calculation of the scale-free fit index of various soft-thresholding powers (β) and analysis of the mean connectivity of various soft-thresholding powers (β). C,D The relationship between hub genes in the blue module and SLC7A11, as well as genes related to disulfidptosis. Additional file 3 : Figure S3 Relationships between SLC7A11 expression and immune cell infiltration in ACC A, B Relationships between SLC7A11 expression and immune cell infiltration. C Relationships between SLC7A11 expression and chemokines. D The Xcell algorithm revealed that the infiltration levels of some immune cells in ACC patients. Additional file 4: Figure S4 Relationship between SLC7A11 expression and drug sensitivity in ACC A-I SLC7A11 expression correlates with the sensitivity of anticancer drugs in ACC patients. Additional file 5: figure S5 haematoxylin-eosin staining of tissue samples A-D HE staining in four ACC samples. Additional file 6: Table S1 The TCGA identifier numbers of ACC samples; Table S2 The clinical information for the 77 ACC patients; Table S3 Two sets of genes associated with disulfidptosis; Table S4 The expression profiles of SLC7A11 in urogenital system-related tumors; Table S5 The 47 immune checkpoint-related genes; Table S6 The 15 overlapping genes of the two sets of genes associated with disulfidptosis; Table S7 The results of the Gene Set Enrichment Analysis (GSEA);Table S8 The q-pcr raw data. [file 12935_2023_3091_MOESM1_ESM.docx]

**Additional files**


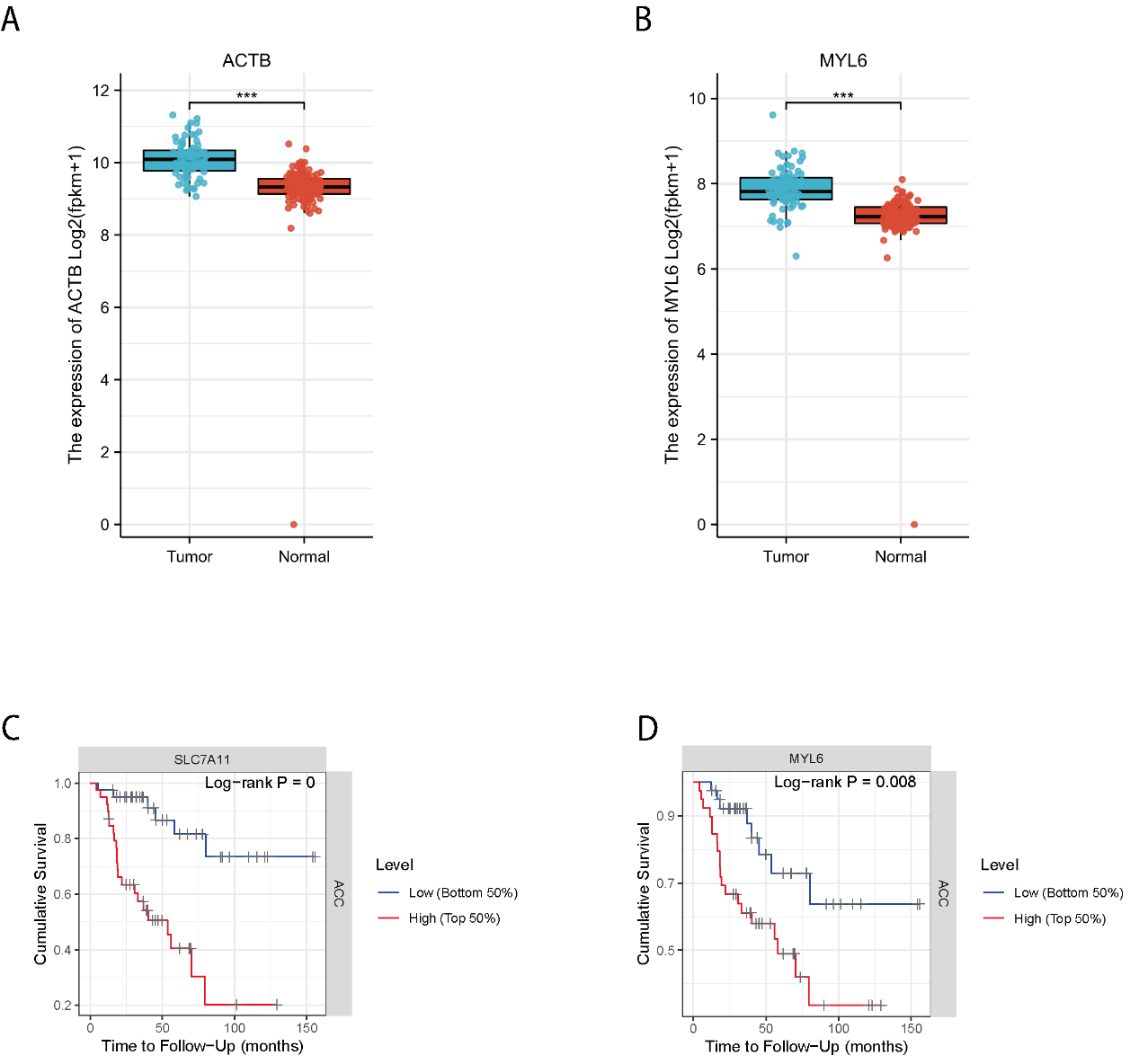


**Additional file 1**: **Figure S1 Expression of disulfidptosis-related genes and their effect on the prognosis of ACC patients A, B** Expression of the genes MYL6 and ACTB in ACC and normal adrenal tissues. **C, D** Kaplan‒Meier survival analysis of SLC7A11 and MYL6 in the TIMER database.


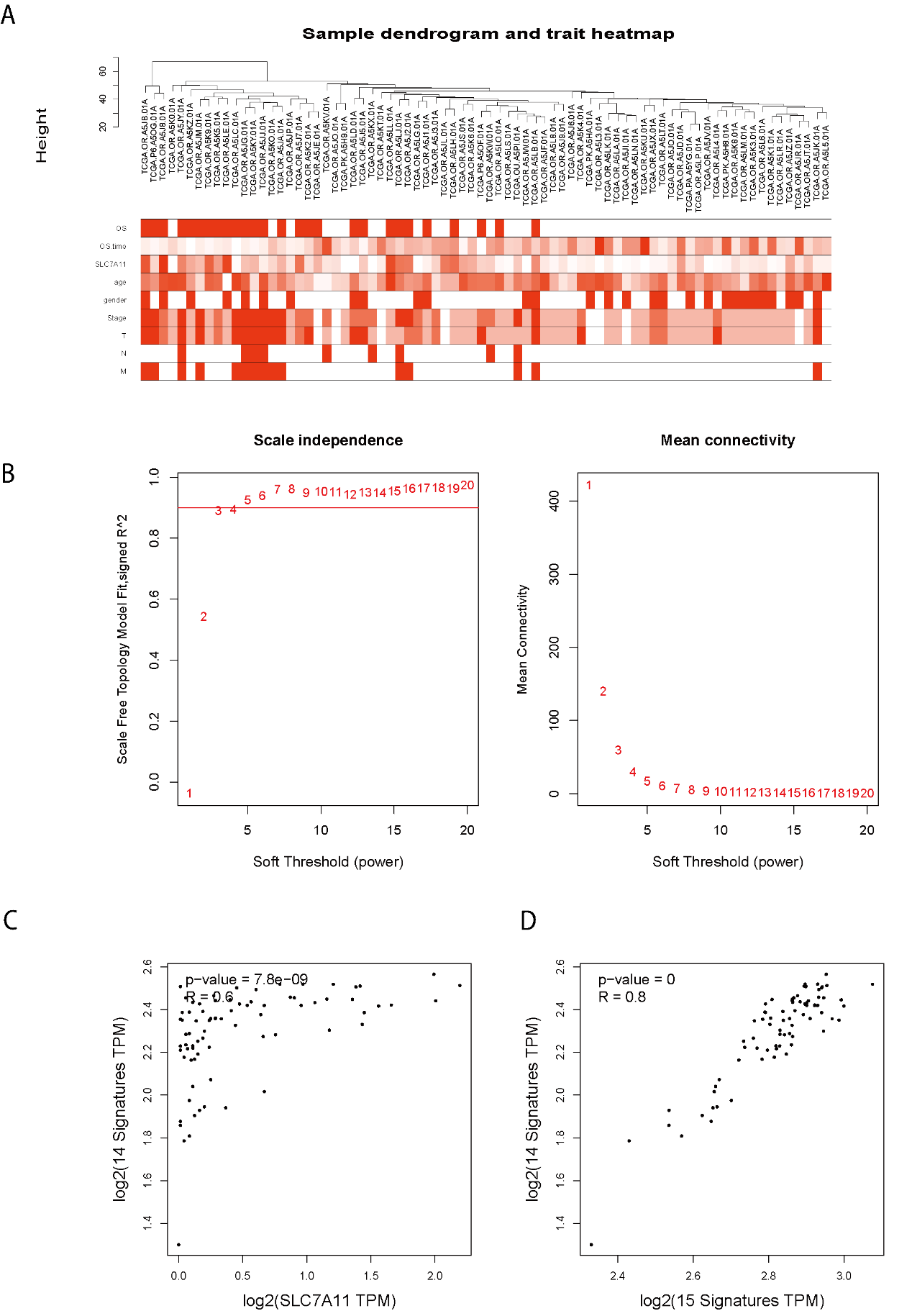


**Additional file 2**: **Figure S2 Sample dendrogram and trait heatmap A** ACC sample dendrogram and trait heatmap.B Calculation of the scale-free fit index of various soft-thresholding powers (β) and analysis of the mean connectivity of various soft-thresholding powers (β). C,D The relationship between hub genes in the blue module and SLC7A11, as well as genes related to disulfidptosis.


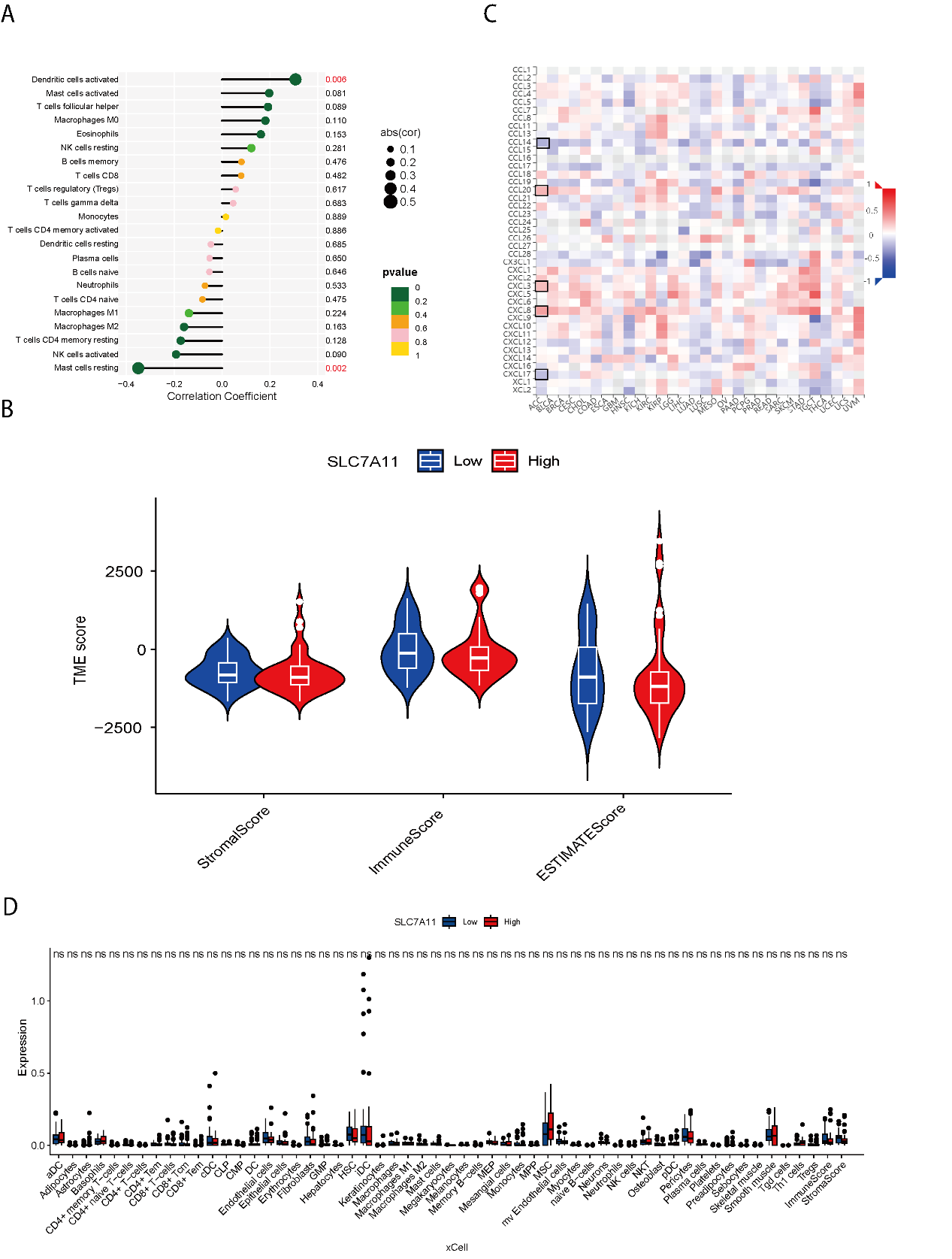


**Additional file 3**: **Figure S3 Relationships between SLC7A11 expression and immune cell infiltration in ACC A, B** Relationships between SLC7A11 expression and immune cell infiltration. **C** Relationships between SLC7A11 expression and chemokines. D The Xcell algorithm revealed that the infiltration levels of some immune cells in ACC patients.


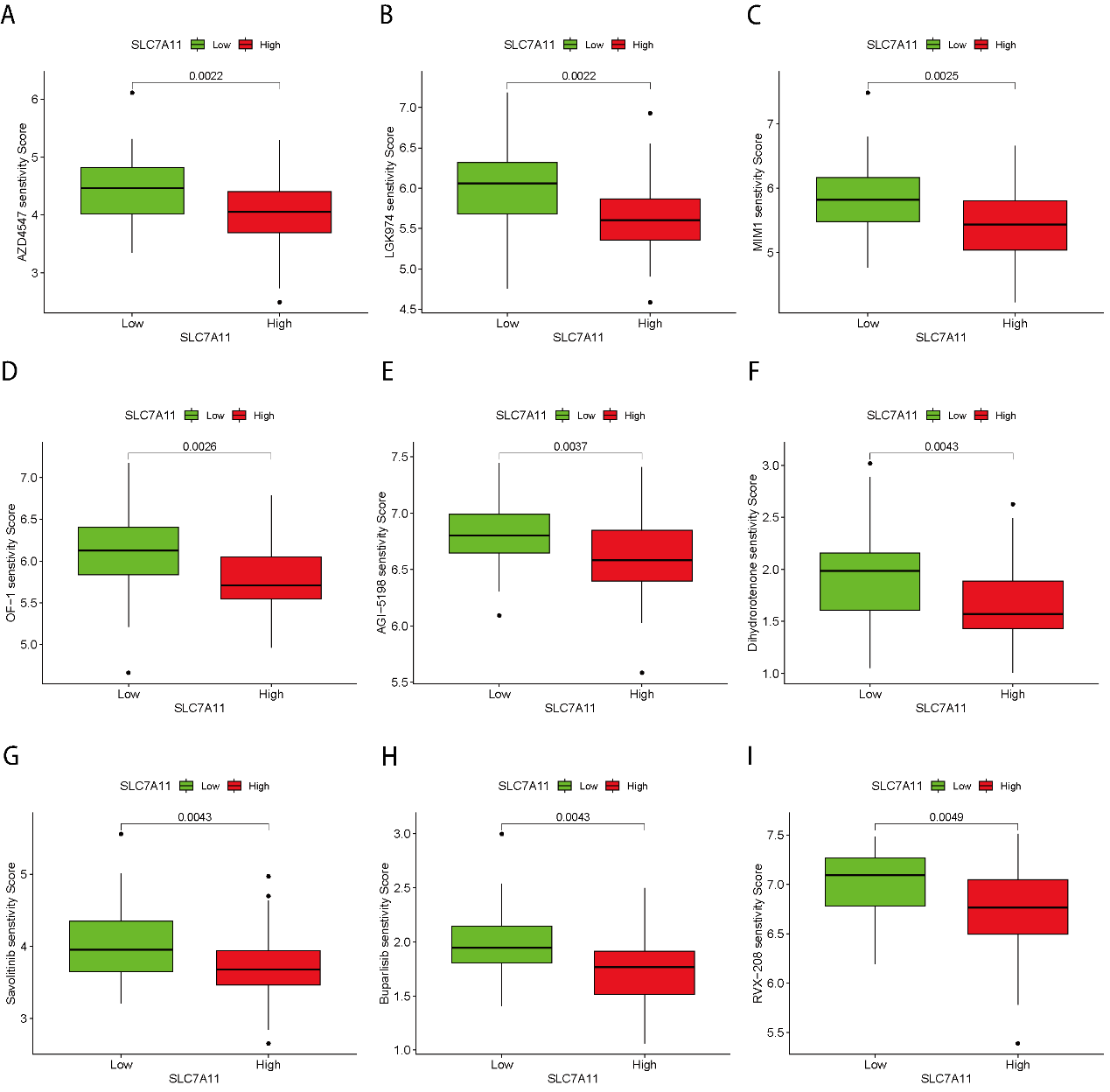


**Additional file 4**: **Figure S4 Relationship between SLC7A11 expression and drug sensitivity in ACC A-I** SLC7A11 expression correlates with the sensitivity of anticancer drugs in ACC patients.


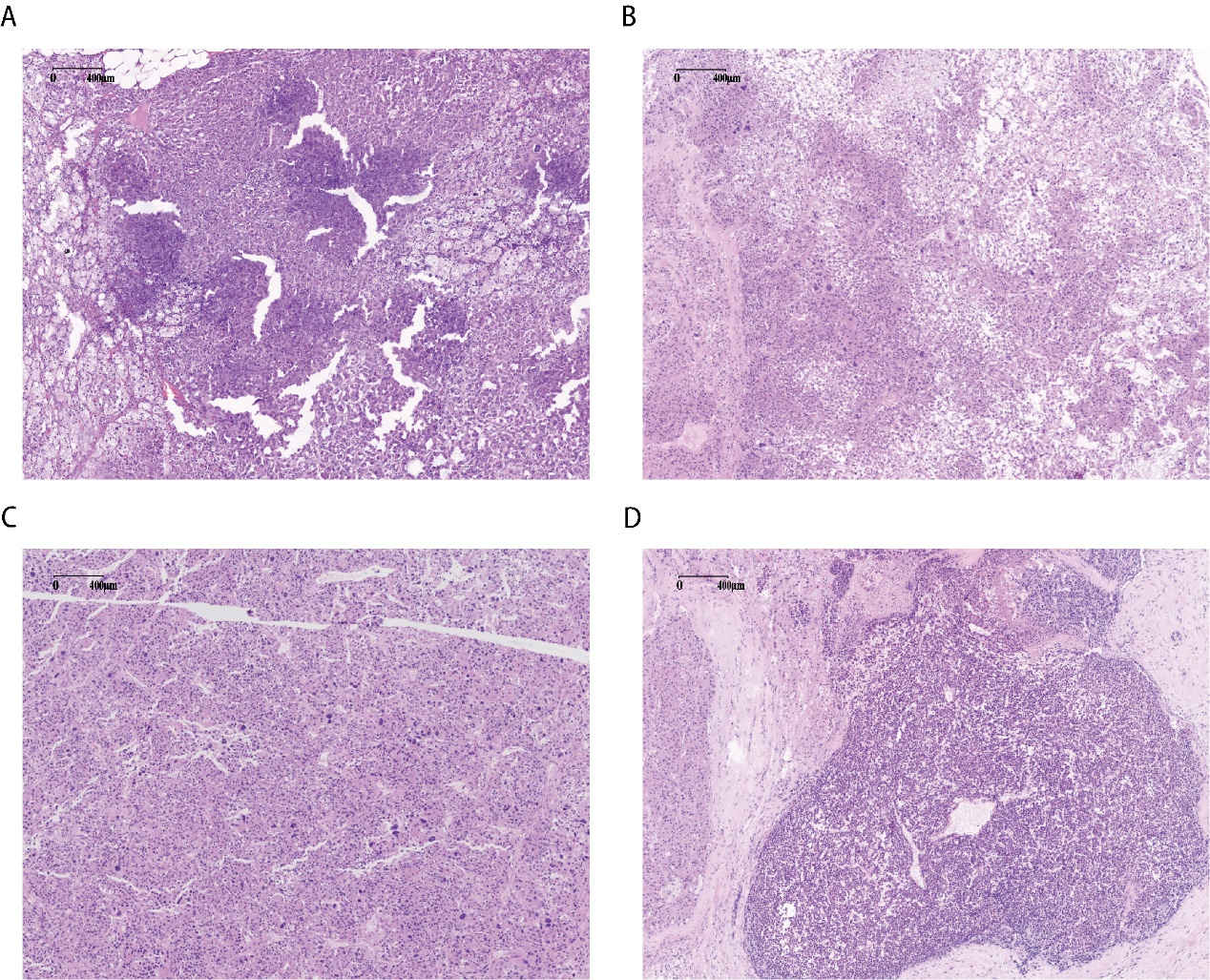


**Additional file 5: Figure S5 Haematoxylin-eosin staining of tissue samples A-D** HE staining in four ACC samples.

**Additional file 6: Supplementary Tables** **Table S1** The TCGA identifier numbers of ACC samples.

| TCGA identifier number(77) | SLC7A11 | Excluded(2) | SLC7A11 |
| --- | --- | --- | --- |
| TCGA.OR.A5JP.01A | 0.2843875 | TCGA.PK.A5HB.01A | 1.054258 |
| TCGA.OR.A5JG.01A | 0.5988327 | TCGA.OR.A5JC.01A | 0.04466227 |
| TCGA.OR.A5LG.01A | 0.01393302 |  |  |
| TCGA.OR.A5JR.01A | 0.2401363 |  |  |
| TCGA.OR.A5KU.01A | 0.02362519 |  |  |
| TCGA.OR.A5LS.01A | 0.07602381 |  |  |
| TCGA.OR.A5L9.01A | 0.009601001 |  |  |
| TCGA.OR.A5JQ.01A | 0.2780425 |  |  |
| TCGA.OR.A5K4.01A | 0.1608422 |  |  |
| TCGA.OR.A5JL.01A | 1.295435 |  |  |
| TCGA.OR.A5LC.01A | 0.05859546 |  |  |
| TCGA.OR.A5K2.01A | 0.2959028 |  |  |
| TCGA.P6.A5OG.01A | 0.3892423 |  |  |
| TCGA.OR.A5JW.01A | 0.03025609 |  |  |
| TCGA.OR.A5KO.01A | 0.3960044 |  |  |
| TCGA.OR.A5J8.01A | 2.024548 |  |  |
| TCGA.PA.A5YG.01A | 0.2771578 |  |  |
| TCGA.OR.A5KV.01A | 0.2713174 |  |  |
| TCGA.OR.A5L4.01A | 0.08093932 |  |  |
| TCGA.OR.A5KX.01A | 0.01200389 |  |  |
| TCGA.OR.A5L8.01A | 0.09377086 |  |  |
| TCGA.OR.A5JO.01A | 0.3155992 |  |  |
| TCGA.OR.A5JA.01A | 0.7209625 |  |  |
| TCGA.OR.A5LO.01A | 0.08842652 |  |  |
| TCGA.OR.A5JM.01A | 0.6466833 |  |  |
| TCGA.OR.A5JV.01A | 0.01511116 |  |  |
| TCGA.OR.A5JB.01A | 1.635583 |  |  |
| TCGA.OR.A5LA.01A | 0.01020291 |  |  |
| TCGA.OR.A5JE.01A | 0.7148107 |  |  |
| TCGA.OR.A5JZ.01A | 0.8037705 |  |  |
| TCGA.OR.A5KT.01A | 0.2045088 |  |  |
| TCGA.OR.A5KZ.01A | 0.7886125 |  |  |
| TCGA.OR.A5LJ.01A | 1.30369 |  |  |
| TCGA.OR.A5LB.01A | 1.058882 |  |  |
| TCGA.OR.A5LT.01A | 0.1236001 |  |  |
| TCGA.OR.A5LD.01A | 0.02293844 |  |  |
| TCGA.OR.A5J2.01A | 1.275045 |  |  |
| TCGA.OR.A5JS.01A | 0.8198039 |  |  |
| TCGA.OR.A5K6.01A | 0.7696782 |  |  |
| TCGA.OR.A5JY.01A | 1.173928 |  |  |
| TCGA.OR.A5JT.01A | 0.5855782 |  |  |
| TCGA.PK.A5H8.01A | 0.1230394 |  |  |
| TCGA.OR.A5JX.01A | 0.1074558 |  |  |
| TCGA.OR.A5LK.01A | 0.1899821 |  |  |
| TCGA.P6.A5OF.01A | 0.4160019 |  |  |
| TCGA.OR.A5JI.01A | 0.08259509 |  |  |
| TCGA.OR.A5K5.01A | 0.8973204 |  |  |
| TCGA.OR.A5KW.01A | 0.4565373 |  |  |
| TCGA.OR.A5JJ.01A | 0.1711093 |  |  |
| TCGA.OR.A5JD.01A | 0.9260435 |  |  |
| TCGA.OR.A5LM.01A | 0.2018428 |  |  |
| TCGA.PK.A5HA.01A | 0.1791842 |  |  |
| TCGA.OR.A5JF.01A | 0.2393984 |  |  |
| TCGA.OR.A5LH.01A | 1.410859 |  |  |
| TCGA.OR.A5K1.01A | 0.06010357 |  |  |
| TCGA.OR.A5LN.01A | 0.1027583 |  |  |
| TCGA.OR.A5LE.01A | 1.856618 |  |  |
| TCGA.OR.A5L3.01A | 0.03557447 |  |  |
| TCGA.OU.A5PI.01A | 0.5091 |  |  |
| TCGA.OR.A5K8.01A | 0.3252325 |  |  |
| TCGA.OR.A5K0.01A | 0.030959 |  |  |
| TCGA.OR.A5KY.01A | 0.6636168 |  |  |
| TCGA.OR.A5J9.01A | 0.2518856 |  |  |
| TCGA.OR.A5J1.01A | 0.1345527 |  |  |
| TCGA.OR.A5J5.01A | 0.5021333 |  |  |
| TCGA.OR.A5L6.01A | 0.251173 |  |  |
| TCGA.OR.A5LP.01A | 0.4717578 |  |  |
| TCGA.OR.A5K9.01A | 1.517337 |  |  |
| TCGA.OR.A5J7.01A | 1.357187 |  |  |
| TCGA.PK.A5H9.01A | 0.00721548 |  |  |
| TCGA.OR.A5LR.01A | 0.1122612 |  |  |
| TCGA.OR.A5JK.01A | 0.1450066 |  |  |
| TCGA.OR.A5J6.01A | 0.06504503 |  |  |
| TCGA.OR.A5LL.01A | 1.756189 |  |  |
| TCGA.OR.A5K3.01A | 0.2451395 |  |  |
| TCGA.OR.A5J3.01A | 0.4117623 |  |  |
| TCGA.OR.A5L5.01A | 0.1397074 |  |  |

**Table S2** The clinical information for the 77 ACC patients;

|  | fustat | futime | SLC7A11 | age | gender | Clinical stage | T stage | N stage | M stage |
| --- | --- | --- | --- | --- | --- | --- | --- | --- | --- |
| TCGA-OR-A5JP-01A | 0 | 1.2712329 | 0.284388 | 40 | male | 2 | 2 | N0 | M0 |
| TCGA-OR-A5JG-01A | 1 | 1.4821918 | 0.598833 | 61 | male | 3 | 4 | N1 | M1 |
| TCGA-OR-A5LG-01A | 0 | 4.3534247 | 0.013933 | 46 | male | 3 | 3 | N0 | M0 |
| TCGA-OR-A5JR-01A | 0 | 10.10411 | 0.240136 | 45 | male | 1 | 1 | N0 | M0 |
| TCGA-OR-A5KU-01A | 0 | 12.80274 | 0.023625 | 37 | female | 2 | 2 | N0 | M0 |
| TCGA-OR-A5LS-01A | 0 | 3.0027397 | 0.076024 | 34 | female | 2 | 2 | N0 | M0 |
| TCGA-OR-A5L9-01A | 0 | 2.3863014 | 0.009601 | 53 | female | 2 | 2 | N0 | M0 |
| TCGA-OR-A5JQ-01A | 0 | 3.0219178 | 0.278043 | 26 | female | 2 | 2 | N0 | M0 |
| TCGA-OR-A5K4-01A | 0 | 2.9643836 | 0.160842 | 64 | female | 3 | 4 | N0 | M0 |
| TCGA-OR-A5JL-01A | 0 | 5.6328767 | 1.295435 | 36 | female | 1 | 1 | N0 | M0 |
| TCGA-OR-A5LC-01A | 1 | 0.4356164 | 0.058595 | 71 | female | 3 | 4 | N0 | M1 |
| TCGA-OR-A5K2-01A | 1 | 2.7232877 | 0.295903 | 32 | female | 3 | 4 | N0 | M0 |
| TCGA-P6-A5OG-01A | 1 | 1.0493151 | 0.389242 | 45 | female | 3 | 4 | N0 | M1 |
| TCGA-OR-A5JW-01A | 0 | 6.0328767 | 0.030256 | 47 | male | 2 | 2 | N0 | M0 |
| TCGA-OR-A5KO-01A | 0 | 3.8739726 | 0.396004 | 39 | female | 3 | 4 | N0 | M1 |
| TCGA-OR-A5J8-01A | 1 | 1.5863014 | 2.024548 | 66 | male | 3 | 3 | N0 | M0 |
| TCGA-PA-A5YG-01A | 0 | 2.0712329 | 0.277158 | 51 | male | 2 | 2 | N0 | M0 |
| TCGA-OR-A5KV-01A | 0 | 10.624658 | 0.271317 | 17 | female | 3 | 2 | N1 | M0 |
| TCGA-OR-A5L4-01A | 0 | 2.6493151 | 0.080939 | 48 | female | 3 | 4 | N0 | M0 |
| TCGA-OR-A5KX-01A | 0 | 3.7369863 | 0.012004 | 25 | female | 3 | 2 | N1 | M0 |
| TCGA-OR-A5L8-01A | 0 | 2.4246575 | 0.093771 | 36 | female | 2 | 2 | N0 | M0 |
| TCGA-OR-A5JO-01A | 0 | 3.2712329 | 0.315599 | 26 | female | 1 | 1 | N0 | M0 |
| TCGA-OR-A5JA-01A | 1 | 2.5260274 | 0.720963 | 53 | female | 3 | 4 | N0 | M1 |
| TCGA-OR-A5LO-01A | 1 | 6.5890411 | 0.088427 | 61 | female | 2 | 2 | N0 | M0 |
| TCGA-OR-A5JM-01A | 1 | 1.539726 | 0.646683 | 25 | female | 3 | 4 | N0 | M1 |
| TCGA-OR-A5JV-01A | 0 | 5.5424658 | 0.015111 | 55 | male | 2 | 2 | N0 | M0 |
| TCGA-OR-A5JB-01A | 1 | 1.509589 | 1.635583 | 52 | male | 3 | 4 | N0 | M1 |
| TCGA-OR-A5LA-01A | 0 | 1.969863 | 0.010203 | 52 | female | 2 | 2 | N0 | M0 |
| TCGA-OR-A5JE-01A | 1 | 5.7671233 | 0.714811 | 17 | female | 1 | 1 | N0 | M0 |
| TCGA-OR-A5JZ-01A | 0 | 2.2520548 | 0.803771 | 60 | male | 2 | 2 | N0 | M0 |
| TCGA-OR-A5KT-01A | 0 | 7.9315068 | 0.204509 | 44 | female | 1 | 1 | N0 | M0 |
| TCGA-OR-A5KZ-01A | 1 | 0.3424658 | 0.788613 | 42 | male | 2 | 2 | N0 | M0 |
| TCGA-OR-A5LJ-01A | 1 | 3.0273973 | 1.30369 | 54 | female | 3 | 2 | N1 | M1 |
| TCGA-OR-A5LB-01A | 1 | 3.2986301 | 1.058882 | 59 | male | 3 | 4 | N0 | M1 |
| TCGA-OR-A5LT-01A | 0 | 1.5041096 | 0.1236 | 57 | male | 3 | 3 | N0 | M0 |
| TCGA-OR-A5LD-01A | 1 | 3.2794521 | 0.022938 | 52 | male | 3 | 4 | N0 | M0 |
| TCGA-OR-A5J2-01A | 1 | 4.5945205 | 1.275045 | 44 | female | 3 | 3 | N0 | M1 |
| TCGA-OR-A5JS-01A | 0 | 1.0493151 | 0.819804 | 65 | female | 2 | 2 | N0 | M0 |
| TCGA-OR-A5K6-01A | 0 | 4.090411 | 0.769678 | 56 | female | 2 | 2 | N0 | M0 |
| TCGA-OR-A5JY-01A | 1 | 1.5123288 | 1.173928 | 68 | female | 3 | 4 | N1 | M1 |
| TCGA-OR-A5JT-01A | 0 | 2.4849315 | 0.585578 | 65 | female | 2 | 2 | N0 | M0 |
| TCGA-PK-A5H8-01A | 0 | 9.9260274 | 0.123039 | 42 | male | 2 | 2 | N0 | M0 |
| TCGA-OR-A5JX-01A | 0 | 2.6027397 | 0.107456 | 50 | male | 3 | 3 | N0 | M0 |
| TCGA-OR-A5LK-01A | 0 | 7.5068493 | 0.189982 | 62 | male | 2 | 2 | N0 | M0 |
| TCGA-P6-A5OF-01A | 1 | 0.5671233 | 0.416002 | 55 | female | 3 | 4 | N0 | M0 |
| TCGA-OR-A5JI-01A | 0 | 6.3863014 | 0.082595 | 22 | male | 1 | 1 | N0 | M0 |
| TCGA-OR-A5K5-01A | 1 | 1.3643836 | 0.89732 | 59 | female | 3 | 3 | N0 | M0 |
| TCGA-OR-A5KW-01A | 0 | 5.6876712 | 0.456537 | 55 | female | 3 | 2 | N1 | M0 |
| TCGA-OR-A5JJ-01A | 1 | 1.3424658 | 0.171109 | 65 | male | 3 | 4 | N1 | M1 |
| TCGA-OR-A5JD-01A | 0 | 8.3232877 | 0.926044 | 57 | female | 2 | 2 | N0 | M0 |
| TCGA-OR-A5LM-01A | 0 | 5.090411 | 0.201843 | 23 | male | 2 | 2 | N0 | M0 |
| TCGA-PK-A5HA-01A | 0 | 3.290411 | 0.179184 | 63 | male | 1 | 1 | N0 | M0 |
| TCGA-OR-A5JF-01A | 0 | 5.5205479 | 0.239398 | 69 | female | 2 | 2 | N0 | M0 |
| TCGA-OR-A5LH-01A | 1 | 6.5342466 | 1.410859 | 36 | female | 2 | 2 | N0 | M0 |
| TCGA-OR-A5K1-01A | 0 | 9.0109589 | 0.060104 | 48 | male | 2 | 2 | N0 | M0 |
| TCGA-OR-A5LN-01A | 0 | 6.4164384 | 0.102758 | 31 | female | 2 | 2 | N0 | M0 |
| TCGA-OR-A5LE-01A | 1 | 1.8136986 | 1.856618 | 14 | male | 2 | 2 | N0 | M0 |
| TCGA-OR-A5L3-01A | 0 | 12.679452 | 0.035574 | 67 | female | 1 | 1 | N0 | M0 |
| TCGA-OU-A5PI-01A | 0 | 3.2082192 | 0.5091 | 53 | female | 3 | 2 | N1 | M1 |
| TCGA-OR-A5K8-01A | 0 | 2.0520548 | 0.325233 | 39 | male | 2 | 2 | N0 | M0 |
| TCGA-OR-A5K0-01A | 0 | 2.8191781 | 0.030959 | 69 | female | 2 | 2 | N0 | M0 |
| TCGA-OR-A5KY-01A | 1 | 1.0712329 | 0.663617 | 23 | female | 3 | 4 | N1 | M1 |
| TCGA-OR-A5J9-01A | 0 | 3.7041096 | 0.251886 | 22 | female | 2 | 2 | N0 | M0 |
| TCGA-OR-A5J1-01A | 1 | 3.7123288 | 0.134553 | 58 | male | 2 | 2 | N0 | M0 |
| TCGA-OR-A5J5-01A | 1 | 1 | 0.502133 | 30 | male | 3 | 4 | N0 | M0 |
| TCGA-OR-A5L6-01A | 0 | 2.3589041 | 0.251173 | 60 | male | 2 | 2 | N0 | M0 |
| TCGA-OR-A5LP-01A | 0 | 5.0876712 | 0.471758 | 37 | female | 2 | 2 | N0 | M0 |
| TCGA-OR-A5K9-01A | 1 | 0.9424658 | 1.517337 | 61 | female | 2 | 2 | N0 | M0 |
| TCGA-OR-A5J7-01A | 1 | 1.3424658 | 1.357187 | 30 | female | 3 | 3 | N0 | M0 |
| TCGA-PK-A5H9-01A | 0 | 1.6876712 | 0.007215 | 27 | female | 2 | 2 | N0 | M0 |
| TCGA-OR-A5LR-01A | 0 | 2.3369863 | 0.112261 | 30 | female | 2 | 2 | N0 | M0 |
| TCGA-OR-A5JK-01A | 0 | 4.1013699 | 0.145007 | 49 | male | 3 | 4 | N0 | M1 |
| TCGA-OR-A5J6-01A | 0 | 7.4054795 | 0.065045 | 29 | female | 2 | 2 | N0 | M0 |
| TCGA-OR-A5LL-01A | 1 | 4.4191781 | 1.756189 | 75 | female | 2 | 2 | N0 | M0 |
| TCGA-OR-A5K3-01A | 0 | 9.4931507 | 0.24514 | 53 | male | 2 | 2 | N0 | M0 |
| TCGA-OR-A5J3-01A | 0 | 5.7287671 | 0.411762 | 23 | female | 3 | 3 | N0 | M0 |
| TCGA-OR-A5L5-01A | 0 | 3.6082192 | 0.139707 | 77 | female | 1 | 1 | N0 | M0 |

**Table S3** Two sets of genes associated with disulfidptosis;

| genelist set of disulfidptosis(1) | genelist set of disulfidptosis(2) |
| --- | --- |
| FLNA | SLC7A11 |
| FLNB | NADPH |
| MYH9 | INF2 |
| TLN1 | CD2AP |
| ACTB | PDLIM1 |
| MYL6 | ACTN4 |
| MYH10 | MYH9 |
| CAPZB | MYH10 |
| DSTN | IQGAP1 |
| IQGAP1 | FLNA |
| ACTN4 | FLNB |
| PDLIM1 | TLN1 |
| CD2AP | MYL6 |
| INF2 | ACTB |
| SLC7A11 | DSTN |
|  | CAPZB |

**Table S4** The expression profiles of SLC7A11 in urogenital system-related tumors;

| （SLC7A11) | ACC | BLCA | KIRC | KICH | KIRP | PRAD | PCPG | TGCT |
| --- | --- | --- | --- | --- | --- | --- | --- | --- |
| Tumor | 0.284566723 | 0.586939695 | 1.538386959 | 0.295731818 | 0.625008249 | 1.911295894 | 0.071492607 | 0.389938144 |
| Tumor | 0.116801691 | 0.655065378 | 0.207660685 | 0.912853428 | 0.196383522 | 0.622618175 | 0.264031929 | 0.287600487 |
| Tumor | 0.548588038 | 0.336035692 | 0.30354527 | 0.054742059 | 0.06703838 | 1.68169508 | 0.345675779 | 1.009652056 |
| Tumor | 0.176567627 | 0.61038913 | 0.171837959 | 0.20684745 | 0.174945211 | 0.380589216 | 0.002824335 | 0.237092899 |
| Tumor | 0.413298365 | 0.210324253 | 0.722819487 | 0.92188873 | 0.332098595 | 1.636393062 | 0.003631678 | 1.014626362 |
| Tumor | 1.853340599 | 0.217191936 | 0.38841292 | 0.607598422 | 2.135942671 | 1.414620795 | 0.042120426 | 0.379455877 |
| Tumor | 0.831715294 | 0.205772264 | 0.356582398 | 0.391114074 | 0 | 0.941777502 | 0.027638782 | 0.322128984 |
| Tumor | 1.326492479 | 0.938412942 | 0.320799168 | 1.874210897 | 0.034564682 | 0.782260576 | 0.163794072 | 0.281549735 |
| Tumor | 1.510363963 | 0.448532411 | 0.327680755 | 0.175915816 | 1.824012733 | 3.308528062 | 0.035970932 | 0.180771248 |
| Tumor | 0.086048552 | 0.164735204 | 0.824279199 | 1.826726916 | 0.798816864 | 1.115730112 | 0.00535684 | 1.19688251 |
| Tumor | 1.341876474 | 0.124428121 | 0.098579822 | 0.193723064 | 0.10212331 | 1.551422526 | 0.035677924 | 0.218713658 |
| Tumor | 0.102882684 | 0.102369524 | 0.999121111 | 0.092358557 | 0.030530247 | 1.033204506 | 0.676357778 | 0.404254749 |
| Tumor | 0.052504916 | 0.610785263 | 0.151656692 | 0.813165006 | 1.071490459 | 1.616124795 | 0.029129781 | 0.904982574 |
| Tumor | 1.354511402 | 1.557579991 | 0.186553717 | 0.163354693 | 2.332896266 | 1.11599928 | 0.409549782 | 0.706035607 |
| Tumor | 0.735918539 | 0.380314965 | 0.222533277 | 0.383980608 | 0.471493476 | 0.552092579 | 0.061976691 | 0.068988243 |
| Tumor | 0.371723669 | 0.285848574 | 0.205772548 | 0.671056705 | 0.182186857 | 1.073172406 | 0.969653372 | 0.310541585 |
| Tumor | 0.027889539 | 2.028578907 | 0.479096957 | 0.348933542 | 0.199736185 | 0.797410437 | 0.099041862 | 0.154482704 |
| Tumor | 0.019933097 | 0.19775258 | 0.384786824 | 0.52013187 | 0.095368289 | 1.105464053 | 0.045393734 | 0.538046508 |
| Tumor | 0.078382032 | 0.068839738 | 0.346832958 | 1.120914186 | 0.636200829 | 0.738457678 | 0.042352449 | 0.217962505 |
| Tumor | 0.103442771 | 0.381226154 | 0.417510005 | 0.173905023 | 4.190543554 | 1.207618409 | 0.035370537 | 0.119106349 |
| Tumor | 0.032452084 | 1.714018784 | 0.188719963 | 0.375968681 | 0.374925917 | 0.462660754 | 0.046763348 | 0.759285578 |
| Tumor | 0.059058844 | 0.186626616 | 0.082731189 | 0.481893288 | 0.099984126 | 1.131316684 | 0.267275803 | 0.390217885 |
| Tumor | 0.165516321 | 2.932203527 | 0.34182006 | 1.897737612 | 0.09669799 | 0.456395539 | 0.08600323 | 0.918205844 |
| Tumor | 0.694245579 | 4.728276858 | 0.087115571 | 0.729930234 | 0.479665538 | 1.624074247 | 0.099532409 | 1.716646867 |
| Tumor | 0.640932198 | 0.590398932 | 0.145696905 | 1.843084913 | 0.312527131 | 1.453962703 | 0.007876227 | 1.238105851 |
| Tumor | 0.42060981 | 1.014501122 | 0.200067632 | 0.371679568 | 0.021767816 | 1.107663148 | 0.050116974 | 0.422979742 |
| Tumor | 0.292593185 | 1.399247595 | 0.199782968 | 0.763480124 | 0.042685316 | 2.428072251 | 0.047909712 | 1.018500694 |
| Tumor | 0.370168076 | 1.303993886 | 0.810390003 | 0.385692532 | 0.19158669 | 1.591588928 | 0.147754839 | 0.454981591 |
| Tumor | 0.060980896 | 0.645862416 | 0.679896188 | 0.462881456 | 0.135931854 | 0.571426803 | 0.039555082 | 0.429278344 |
| Tumor | 0.035987156 | 4.740682954 | 0.411817078 | 1.871288947 | 0.304818699 | 0.527507258 | 0.111546325 | 0.955572918 |
| Tumor | 0.195809187 | 0.345743123 | 0.265743834 | 1.726012696 | 0.113828988 | 1.494793368 | 0.126851055 | 0.411534317 |
| Tumor | 0.066760512 | 1.433144095 | 0.418815961 | 0.651105297 | 0.017708903 | 1.107087491 | 0.11275078 | 0.312620605 |
| Tumor | 0.127834873 | 2.580072704 | 1.585175736 | 0.048786031 | 0.239138112 | 0.75746151 | 0.943605022 | 0.639706452 |
| Tumor | 0.128573386 | 0.855641907 | 0.621220956 | 0.929707415 | 0.050811743 | 0.894103077 | 0.16790543 | 0.605628459 |
| Tumor | 0.013191895 | 0.337928416 | 0.204301913 | 1.979386209 | 0.183886532 | 1.570925146 | 0.060098895 | 0.171527625 |
| Tumor | 1.783008584 | 0.495344622 | 0.141968054 | 0.126545396 | 0.44273611 | 0.376447982 | 0.03499661 | 1.032152966 |
| Tumor | 0.459824713 | 0.246136656 | 0.361293982 | 0.154378383 | 0.409131594 | 0.599436838 | 0.018631722 | 1.368485639 |
| Tumor | 1.611862159 | 0.589237658 | 0.060372522 | 0.572397506 | 0.016118318 | 1.878313509 | 0.021182607 | 1.699729332 |
| Tumor | 0.405404056 | 1.500420593 | 0.864742458 | 0.402875125 | 0.729707114 | 0.238236078 | 0.197399735 | 1.047751647 |
| Tumor | 0.629257383 | 0.401722069 | 0.702380239 | 0.209398123 | 0.399030231 | 1.085974228 | 0.201448127 | 0.384913217 |
| Tumor | 0.030994447 | 2.318787307 | 0.185071246 | 1.23527008 | 0.104412939 | 0.506816153 | 0.153467267 | 0.206230973 |
| Tumor | 0.257105386 | 0.442293646 | 0.051823675 | 0.341132919 | 0.061571305 | 0.5092045 | 0.015032679 | 0.593640919 |
| Tumor | 0.818440921 | 0.528579033 | 0.792540422 | 0.091461961 | 0.563161894 | 1.237479802 | 0.129754065 | 0.3311494 |
| Tumor | 1.027464077 | 0.7998515 | 0.405764586 | 1.402616389 | 0.156318929 | 1.085924002 | 0.10285129 | 0.693615971 |
| Tumor | 0.150017979 | 0.25852098 | 0.266185706 | 0.148461651 | 0.067433575 | 1.627033905 | 0.0414511 | 0.707997395 |
| Tumor | 0.018166511 | 0.136607183 | 1.443873458 | 1.299848574 | 0.194127397 | 0.875625051 | 0.100703346 | 0.999486944 |
| Tumor | 0.167802102 | 6.833516798 | 0.083502152 | 0.190357147 | 0.185789059 | 0.977049802 | 0.056672464 | 0.159337018 |
| Tumor | 0.161034386 | 0.662831229 | 0.300678557 | 1.205155996 | 0.312724502 | 1.245889218 | 0.111470746 | 1.227339184 |
| Tumor | 0.096034189 | 0.558856471 | 0.353124589 | 2.919046926 | 0.152081897 | 0.820564191 | 0.686098731 | 0.18443525 |
| Tumor | 0.683760942 | 0.181074613 | 0.296049807 | 2.657867206 | 0.175047795 | 1.779520618 | 0.16168016 | 0.925453795 |
| Tumor | 0.014710666 | 0.242888545 | 0.867390959 | 0.4361097 | 0.101254777 | 2.03914087 | 0.075869932 | 0.399644247 |
| Tumor | 1.433129315 | 0.705161373 | 0.410269472 | 0.588762156 | 0.169273853 | 1.658384243 | 0.044925603 | 0.250979787 |
| Tumor | 1.113435803 | 1.035668923 | 0.198784842 | 0.693011893 | 0.078439645 | 0.804968301 | 0.038921741 | 0.310827779 |
| Tumor | 0.234620753 | 0.159917601 | 0.079693602 | 0.452453676 | 1.095279188 | 0.061851834 | 0.086747397 | 1.010282203 |
| Tumor | 0.302599578 | 1.904789947 | 0.367996355 | 0.33437047 | 0.054420174 | 1.133428493 | 0.02286977 | 1.528274229 |
| Tumor | 0.273577833 | 1.18969626 | 0.125344823 | 0.436498975 | 0.978231895 | 1.498290416 | 0.026269489 | 0.142867376 |
| Tumor | 1.991014382 | 5.075638632 | 0.374014861 | 0.46939099 | 0.051088329 | 0.437026598 | 0.355412505 | 0.709224617 |
| Tumor | 0.261497117 | 0.931960641 | 0.579880542 | 0.510913881 | 0.385385856 | 1.335454638 | 0.059950817 | 0.154959923 |
| Tumor | 0.704704761 | 0.56612369 | 0.103387698 | 0.171594145 | 0.187909778 | 0.781669257 | 0.210553974 | 0.226667284 |
| Tumor | 1.23403588 | 0.768924408 | 0.382172107 | 0.127098757 | 0.438461171 | 1.655164926 | 0.260161356 | 0.614255486 |
| Tumor | 0.029445026 | 1.955654315 | 0.27721985 | 0.352627992 | 0.056317741 | 0.737513045 | 0.05540902 | 0.279174166 |
| Tumor | 0.619959746 | 0.292230465 | 0.164863008 | 1.208976905 | 0.071423565 | 0.216423058 | 0.718764329 | 1.157106726 |
| Tumor | 0.155210647 | 1.445434696 | 0.303540747 | 0.44643727 | 0.365261022 | 0.699715118 | 0.022990475 | 0.671138338 |
| Tumor | 1.516220943 | 1.379138839 | 0.327723505 | 0.567220217 | 0.097629325 | 0.949425781 | 0.01262136 | 0.827413344 |
| Tumor | 0.56125711 | 0.158280235 | 0.269716965 | 0.207879668 | 0.231325692 | 1.115403573 | 0.004688668 | 0.245029388 |
| Tumor | 0.777775535 | 4.112717194 | 0.133525078 |  | 0.112428162 | 0.335189227 | 0.083771664 | 0.38330799 |
| Tumor | 0.863599536 | 0.240977914 | 0.372400066 |  | 0.060204685 | 1.715369534 | 0.068512701 | 0.262133037 |
| Tumor | 0.19336666 | 1.123671391 | 0.445969007 |  | 0.88159448 | 0.312590272 | 0.026362342 | 0.247807921 |
| Tumor | 0.260066376 | 0.965824675 | 0.666417245 |  | 0.748713365 | 0.560542194 | 0.101392679 | 0.765553927 |
| Tumor | 0.431771206 | 1.127627174 | 0.507822401 |  | 0.025005834 | 1.04545231 | 0.143553493 | 0.53288626 |
| Tumor | 1.189601299 | 0.448922147 | 0.487866694 |  | 0.057777003 | 1.424362876 | 0.030370819 | 0.283438396 |
| Tumor | 0.007941502 | 0.305979922 | 0.599245507 |  | 0.528245197 | 1.065712525 | 0.103142592 | 1.158242251 |
| Tumor | 0.238158661 | 0.967375001 | 0.242764345 |  | 1.402383902 | 1.530091088 | 0.017634877 | 0.444945639 |
| Tumor | 0.483973714 | 5.69051085 | 0.242411019 |  | 0.048424433 | 0.39462702 | 0.295509705 | 0.826358026 |
| Tumor | 0.341815232 | 0.282957575 | 0.2641937 |  | 0.106547848 | 1.474427237 | 0.538285962 | 1.253718444 |
| Tumor | 0.322096015 | 0.228925114 | 0.133496167 |  | 0.146598279 | 0.777518502 | 0.024522708 | 1.230979975 |
| Tumor | 0.823649591 | 0.618762473 | 0.361749943 |  | 0.199599402 | 0.928314991 | 0.067312153 | 0.331056198 |
| Tumor |  | 0.284926337 | 0.599422317 |  | 0.048180432 | 1.290037983 | 0.129145669 | 0.683876238 |
| Tumor |  | 0.364607364 | 0.549325617 |  | 0.174258161 | 1.823093018 | 0.379350394 | 0.325899884 |
| Tumor |  | 0.979550458 | 0.498937593 |  | 0.055889197 | 1.75663722 | 0.14521052 | 2.10995733 |
| Tumor |  | 0.294894105 | 0.071817 |  | 0.230984333 | 1.670276366 | 0.045395708 | 2.079929161 |
| Tumor |  | 0.30898967 | 0.676449662 |  | 0.082571298 | 0.994678381 | 0.035066342 | 0.567142407 |
| Tumor |  | 0.568097115 | 0.084889429 |  | 0.198884721 | 0.486741572 | 0.280797561 | 1.534540548 |
| Tumor |  | 0.566948724 | 0.267624733 |  | 0.102435084 | 1.132002096 | 0.334850199 | 0.640002505 |
| Tumor |  | 0.635951877 | 0.407529865 |  | 2.813612459 | 0.640927618 | 0.191719919 | 0.434850725 |
| Tumor |  | 2.023097788 | 0.281774634 |  | 0.26241307 | 1.007157569 | 0.09902701 | 0.449701348 |
| Tumor |  | 0.608965828 | 0.353739636 |  | 0.129038508 | 1.210247758 | 0.035470109 | 0.548838622 |
| Tumor |  | 0.087484259 | 0.133185356 |  | 0.198297813 | 0.74325855 | 0.044471138 | 1.235253915 |
| Tumor |  | 0.681527872 | 0.961039207 |  | 1.375254283 | 0.384964065 | 0.027075733 | 0.188115032 |
| Tumor |  | 0.46955436 | 0.929213898 |  | 0.079438957 | 1.14594346 | 0.112824778 | 1.89882558 |
| Tumor |  | 2.065195498 | 0.733865751 |  | 0.089604513 | 2.666868794 | 0.019789351 | 1.947462792 |
| Tumor |  | 1.829827191 | 0.668360352 |  | 0.880899328 | 1.169361676 | 0.044469391 | 0.36422927 |
| Tumor |  | 2.961841895 | 1.816198339 |  | 0.641289333 | 0.212434049 | 0.042151946 | 0.93003905 |
| Tumor |  | 0.719656507 | 0.320592349 |  | 2.700824045 | 0.554794387 | 0.138114299 | 0.558251923 |
| Tumor |  | 0.681913243 | 0.152878082 |  | 0.136820746 | 0.611134144 | 0.040376257 | 1.367217458 |
| Tumor |  | 1.268822973 | 0.359050809 |  | 0.256117464 | 1.730706907 | 0.031996079 | 0.316745912 |
| Tumor |  | 0.53477048 | 0.192649431 |  | 0.649418698 | 1.815033717 | 0.072008877 | 0.433698617 |
| Tumor |  | 0.176508849 | 0.350357037 |  | 0.072344417 | 0.673252987 | 1.387689547 | 0.69406178 |
| Tumor |  | 0.285021101 | 1.30523235 |  | 0.389368913 | 1.381501177 | 0.155735878 | 1.085032437 |
| Tumor |  | 0.59136757 | 0.171046092 |  | 0.808594522 | 1.70563675 | 0.003680096 | 1.943802905 |
| Tumor |  | 0.469977523 | 1.724174041 |  | 0.062221614 | 0.749323152 | 0.044814414 | 0.837032822 |
| Tumor |  | 0.555216049 | 0.199553768 |  | 1.515409642 | 1.207952791 | 0.8769786 | 0.454083926 |
| Tumor |  | 0.13368967 | 0.115578539 |  | 0.27179225 | 2.246919752 | 0.203642525 | 0.238219443 |
| Tumor |  | 0.248599407 | 0.297240844 |  | 0.112912647 | 0.499126766 | 0.043850885 | 0.326281972 |
| Tumor |  | 0.197163738 | 0.166390675 |  | 0.118493286 | 1.758422982 | 0.132600719 | 1.927205351 |
| Tumor |  | 1.175951803 | 0.250317386 |  | 0.038444099 | 1.264505178 | 0.659248777 | 1.130152998 |
| Tumor |  | 0.619782198 | 0.309400182 |  | 0.076299077 | 1.555074572 | 0.415198354 | 1.131962609 |
| Tumor |  | 0.148625213 | 0.260197216 |  | 3.64580402 | 0.945391454 | 0.78510967 | 0.170053177 |
| Tumor |  | 1.333906311 | 0.527135856 |  | 0.30934985 | 1.170823875 | 0.004061798 | 1.21885173 |
| Tumor |  | 0.456640052 | 0.546600754 |  | 0.242107009 | 1.084501643 | 0.020938229 | 1.360147514 |
| Tumor |  | 1.56747816 | 0.58240561 |  | 1.399198211 | 1.234813735 | 0.150068638 | 1.112028514 |
| Tumor |  | 0.968006599 | 0.115629746 |  | 4.075564046 | 0.129584476 | 0.056277962 | 0.553948627 |
| Tumor |  | 4.63335653 | 0.251172348 |  | 2.936822067 | 0.784413458 | 0.051667666 | 0.66732906 |
| Tumor |  | 0.135512637 | 0.349497271 |  | 0.299097812 | 2.007481879 | 0.134214237 | 0.319305184 |
| Tumor |  | 0.544352433 | 0.146041653 |  | 0.532539879 | 0.514942269 | 0.024038834 | 0.086190862 |
| Tumor |  | 2.434257934 | 0.278765429 |  | 0.617329516 | 0.964798754 | 0.295847047 | 0.134457199 |
| Tumor |  | 0.754046226 | 0.935645494 |  | 0.238560971 | 1.960112097 | 0.020868103 | 1.232506155 |
| Tumor |  | 0.415895867 | 1.761921681 |  | 0.204657261 | 0.813579638 | 0.069460676 | 0.599987803 |
| Tumor |  | 3.096602657 | 0.113909726 |  | 0.606436176 | 0.84238273 | 0.060072045 | 0.261336695 |
| Tumor |  | 0.462647294 | 0.26037269 |  | 0.418073826 | 1.248877343 | 0.017378946 | 0.816186232 |
| Tumor |  | 0.290311569 | 0.294213318 |  | 3.195496001 | 0.907652522 | 0.020342668 | 0.853494051 |
| Tumor |  | 3.930576597 | 0.495119392 |  | 0.134985188 | 2.440646168 | 0.060707733 | 1.952636926 |
| Tumor |  | 1.122190621 | 0.261164202 |  | 0.061336838 | 1.447654688 | 0.056016885 | 0.240592843 |
| Tumor |  | 0.640451958 | 1.009096785 |  | 0.29858652 | 1.305554553 | 0.126092599 | 1.096669281 |
| Tumor |  | 0.964278868 | 0.883617092 |  | 3.702912882 | 1.136636758 | 0.020446792 | 0.135205775 |
| Tumor |  | 0.426680232 | 1.704352991 |  | 0.343746169 | 0.729466824 | 0.004351238 | 0.244053276 |
| Tumor |  | 1.039505617 | 0.240547881 |  | 3.458824699 | 0.137266173 | 0.026309865 | 1.901472296 |
| Tumor |  | 0.315346469 | 0.200717284 |  | 2.460562721 | 1.451982413 | 0.084321048 | 0.207971729 |
| Tumor |  | 1.143209559 | 0.545388436 |  | 0.02910461 | 0.85823485 | 0.12411827 | 0.921097849 |
| Tumor |  | 0.689229325 | 0.2694336 |  | 0.545169719 | 0.821907177 | 0.004512933 | 0.833169036 |
| Tumor |  | 1.430770668 | 0.272052797 |  | 0.044231248 | 0.478614764 | 0.091271013 | 0.467168274 |
| Tumor |  | 0.381021773 | 0.14511226 |  | 0.039513886 | 2.186973967 | 0.055194068 | 1.119459591 |
| Tumor |  | 0.317751259 | 0.685466374 |  | 1.64618861 | 1.081323037 | 0.007879313 | 0.635130917 |
| Tumor |  | 0.408692639 | 0.357734863 |  | 0.031828833 | 0.737391054 | 0.640969482 | 0.117315077 |
| Tumor |  | 0.803357965 | 0.570340652 |  | 0.039644985 | 0.481074323 | 0.034591601 | 1.350054364 |
| Tumor |  | 0.820621245 | 0.913302198 |  | 0.153454423 | 1.080436889 | 0.080142408 | 0.975579339 |
| Tumor |  | 0.570008132 | 0.544223631 |  | 0.177805735 | 0.47707568 | 0.229298303 | 0.139192525 |
| Tumor |  | 0.772172432 | 0.288640585 |  | 0.307327319 | 1.023142167 | 0.177438721 | 0.497402135 |
| Tumor |  | 0.535581433 | 0.198914002 |  | 0.51910363 | 0.901735904 | 0.079440463 | 0.239336549 |
| Tumor |  | 0.257968102 | 0.789635905 |  | 0.267869627 | 0.885452029 | 0.082903108 | 0.910806396 |
| Tumor |  | 0.032034107 | 0.774676666 |  | 0.377292956 | 0.798776653 | 0.081616862 | 0.234318858 |
| Tumor |  | 0.67044247 | 0.41921584 |  | 0.034057106 | 2.035904891 | 0.014280055 | 1.221278482 |
| Tumor |  | 0.32050582 | 0.251777492 |  | 0.143044323 | 0.330225959 | 0.36683204 | 0.902459657 |
| Tumor |  | 0.051768293 | 0.257362187 |  | 0.141332808 | 1.231751042 | 0.300278414 | 1.916756803 |
| Tumor |  | 0.319133592 | 1.40602565 |  | 2.556489777 | 1.712807103 | 0.233536805 | 0.240767142 |
| Tumor |  | 0.093082473 | 0.26692732 |  | 0.031040223 | 0.193835654 | 1.175277301 | 0.236141104 |
| Tumor |  | 2.546379214 | 0.284857689 |  | 0.123689787 | 0.60392377 | 0.130673551 | 0.275325641 |
| Tumor |  | 0.186396504 | 0.449032468 |  | 0.256819345 | 0.828716976 | 0.009070056 | 0.83425068 |
| Tumor |  | 0.736269675 | 0.664806906 |  | 2.708333471 | 1.036157886 | 0.077259261 | 0.114821186 |
| Tumor |  | 0.494583515 | 0.302724659 |  | 0.920535837 | 0.897721208 | 0.257512923 | 0.679378535 |
| Tumor |  | 0.495464678 | 0.57932787 |  | 0.63439272 | 1.301563237 | 0.004512739 | 1.055814344 |
| Tumor |  | 0.071939656 | 0.250617386 |  | 0.465050929 | 1.308632395 | 0.147837007 | 0.567597367 |
| Tumor |  | 1.223876987 | 0.095418231 |  | 0.06054243 | 1.493954745 | 0.093606578 | 0.29208023 |
| Tumor |  | 0.386712203 | 0.679128455 |  | 0.044203596 | 0.562383488 | 0.073270432 | 0.611141898 |
| Tumor |  | 1.270826104 | 0.419270139 |  | 0.286996601 | 0.210210046 | 0.12793795 |  |
| Tumor |  | 0.769874663 | 0.505925177 |  | 0.121328374 | 1.587540945 | 0.138996957 |  |
| Tumor |  | 0.285063709 | 0.056624933 |  | 0.045025633 | 0.665812352 | 0.054673259 |  |
| Tumor |  | 1.369072371 | 0.325964412 |  | 0.451264692 | 1.06018869 | 0.243258562 |  |
| Tumor |  | 0.577576857 | 0.563248803 |  | 0.050590534 | 0.647990705 | 0.147764297 |  |
| Tumor |  | 3.89058242 | 0.374394748 |  | 0.603223839 | 1.033293408 | 0.135286481 |  |
| Tumor |  | 0.621659079 | 0.511170249 |  | 0.064984189 | 1.223332917 | 0.074771833 |  |
| Tumor |  | 0.158228649 | 0.29403524 |  | 0.145816259 | 0.236284332 | 0.113394068 |  |
| Tumor |  | 0.412598482 | 0.389958513 |  | 0.14886922 | 0.839704369 | 0.467620138 |  |
| Tumor |  | 0.844370346 | 0.111424524 |  | 2.334229103 | 1.335300454 | 0 |  |
| Tumor |  | 1.163753115 | 1.816808514 |  | 0.292475049 | 1.10898062 | 0.195420483 |  |
| Tumor |  | 1.53794827 | 0.422296367 |  | 0.10032005 | 1.253500759 | 0.085649557 |  |
| Tumor |  | 0.221922293 | 0.560453019 |  | 0.138079683 | 0.323001617 | 0.103730755 |  |
| Tumor |  | 0.302502801 | 0.968111432 |  | 2.850210133 | 0.505400284 | 0.787851111 |  |
| Tumor |  | 0.584856111 | 0.655732531 |  | 0.325462523 | 0.719516157 | 0.350729128 |  |
| Tumor |  | 1.423582384 | 0.01088926 |  | 1.435416403 | 1.523547478 | 0.009207178 |  |
| Tumor |  | 0.750358753 | 0.356111466 |  | 0.26936489 | 1.407686795 | 0.10610953 |  |
| Tumor |  | 0.434225352 | 0.154313074 |  | 0.075152932 | 1.901058309 | 0.328486621 |  |
| Tumor |  | 0.252039941 | 0.469423961 |  | 0.16268168 | 0.33752801 | 0.023703207 |  |
| Tumor |  | 0.537866554 | 0.472754151 |  | 0.330065896 | 0.680252197 | 0.022208001 |  |
| Tumor |  | 0.712017732 | 0.061091691 |  | 0.132342353 | 1.305984979 | 0.089268019 |  |
| Tumor |  | 0.352343384 | 0.582162164 |  | 1.595727143 | 0.791226391 |  |  |
| Tumor |  | 0.817697067 | 0.219448526 |  | 0.061309108 | 0.584028417 |  |  |
| Tumor |  | 0.531409253 | 0.069703851 |  | 0.07077439 | 0.871244283 |  |  |
| Tumor |  | 0.745667293 | 0.213882809 |  | 0.81243337 | 2.246541061 |  |  |
| Tumor |  | 0.280862042 | 0.909941732 |  | 0.409161246 | 0.729976685 |  |  |
| Tumor |  | 1.048832285 | 0.110888232 |  | 0.079719579 | 1.469498005 |  |  |
| Tumor |  | 1.391443449 | 0.571513695 |  | 0.118743294 | 1.122829758 |  |  |
| Tumor |  | 1.030654994 | 0.709688554 |  | 0.295040259 | 1.285425659 |  |  |
| Tumor |  | 0.580343986 | 1.055275496 |  | 0.486826825 | 1.352840026 |  |  |
| Tumor |  | 0.098640128 | 0.469751859 |  | 0.362615512 | 1.618406117 |  |  |
| Tumor |  | 0.494562705 | 1.439415695 |  | 0.065743814 | 0.537456709 |  |  |
| Tumor |  | 1.49158044 | 0.418590598 |  | 2.580842228 | 0.621021675 |  |  |
| Tumor |  | 7.1715101 | 0.138666243 |  | 0.011483026 | 1.00206061 |  |  |
| Tumor |  | 0.454415259 | 0.335557263 |  | 0.23194084 | 0.745881484 |  |  |
| Tumor |  | 0.062606254 | 0.224913236 |  | 1.792051997 | 0.476854706 |  |  |
| Tumor |  | 0.068195034 | 0.180344224 |  | 0.011400474 | 1.236176434 |  |  |
| Tumor |  | 0.498960729 | 0.380483335 |  | 0.020656218 | 1.226254665 |  |  |
| Tumor |  | 1.026092568 | 0.159630612 |  | 0.338918559 | 0.981493688 |  |  |
| Tumor |  | 1.307057689 | 0.139544339 |  | 0.037114631 | 0.694454329 |  |  |
| Tumor |  | 0.200267331 | 0.116449759 |  | 0.100959814 | 1.593692883 |  |  |
| Tumor |  | 0.273870256 | 0.239834409 |  | 0.041280963 | 1.24201612 |  |  |
| Tumor |  | 0.752572938 | 0.314169387 |  | 0.096629535 | 0.688220941 |  |  |
| Tumor |  | 0.148109234 | 0.125240344 |  | 5.131230265 | 1.051778163 |  |  |
| Tumor |  | 0.217525793 | 0.333492427 |  | 0.429438966 | 1.931137178 |  |  |
| Tumor |  | 1.740642642 | 0.648107173 |  | 0.3531617 | 0.500103695 |  |  |
| Tumor |  | 6.242704183 | 0.526094603 |  | 0.224622941 | 1.346355388 |  |  |
| Tumor |  | 4.1360187 | 0.497139665 |  | 0.047260372 | 0.540882025 |  |  |
| Tumor |  | 1.198603981 | 0.288063138 |  | 0.112536877 | 4.145644043 |  |  |
| Tumor |  | 0.581111989 | 0.114817462 |  | 0.150290408 | 1.751040486 |  |  |
| Tumor |  | 0.285864594 | 0.384290177 |  | 0.160381703 | 0.371069668 |  |  |
| Tumor |  | 1.018550777 | 0.149829138 |  | 0.022862843 | 1.951286472 |  |  |
| Tumor |  | 2.237018914 | 0.597983849 |  | 0.109139372 | 0.744602419 |  |  |
| Tumor |  | 0.459731965 | 0.214133939 |  | 0.188212014 | 0.979119311 |  |  |
| Tumor |  | 0.458134532 | 1.459581723 |  | 0.056155372 | 0.728948976 |  |  |
| Tumor |  | 0.302138798 | 0.281784291 |  | 0.198965769 | 2.035931996 |  |  |
| Tumor |  | 5.577791239 | 0.300996433 |  | 0.223810079 | 0.781349768 |  |  |
| Tumor |  | 0.71467062 | 0.355977624 |  | 0.021595909 | 2.208115334 |  |  |
| Tumor |  | 1.258274585 | 0.304765456 |  | 0.018782011 | 0.515766187 |  |  |
| Tumor |  | 0.780876536 | 0.283520686 |  | 3.54834015 | 0.750322967 |  |  |
| Tumor |  | 0.228356714 | 0.259762066 |  | 0.043436953 | 1.786000219 |  |  |
| Tumor |  | 0.300422047 | 0.400022459 |  | 1.960000979 | 0.492789417 |  |  |
| Tumor |  | 0.829486364 | 0.544065605 |  | 0.041791713 | 0.681523124 |  |  |
| Tumor |  | 0.796488977 | 0.507865619 |  | 0.140146391 | 0.479615019 |  |  |
| Tumor |  | 1.659226182 | 0.189437428 |  | 0.078275571 | 1.285291328 |  |  |
| Tumor |  | 0.863342318 | 0.536921683 |  | 0.174758281 | 1.21660873 |  |  |
| Tumor |  | 0.538146532 | 0.230113337 |  | 0.377411543 | 2.972547433 |  |  |
| Tumor |  | 0.939839998 | 0.838250315 |  | 0.171685143 | 1.130066166 |  |  |
| Tumor |  | 0.505523485 | 0.459720268 |  | 0.40674387 | 2.169083922 |  |  |
| Tumor |  | 0.099150903 | 0.798058859 |  | 0.037187611 | 0.846038372 |  |  |
| Tumor |  | 0.737599136 | 0.18673301 |  | 0.734659136 | 1.231549854 |  |  |
| Tumor |  | 3.970333704 | 0.213199798 |  | 0.067592151 | 2.071360586 |  |  |
| Tumor |  | 2.143423637 | 0.813744063 |  | 0.759423361 | 1.317547581 |  |  |
| Tumor |  | 0.476730719 | 1.337813172 |  | 2.567172724 | 2.021439906 |  |  |
| Tumor |  | 0.285559448 | 0.679287116 |  | 0.496729719 | 1.37325912 |  |  |
| Tumor |  | 0.215916466 | 0.419063958 |  | 0.225648495 | 1.439101837 |  |  |
| Tumor |  | 1.252981797 | 0.101739163 |  | 0.153204419 | 0.791635404 |  |  |
| Tumor |  | 0.305073506 | 0.319062703 |  | 0.091998587 | 0.105975985 |  |  |
| Tumor |  | 0.81985208 | 0.507263938 |  | 0.072610737 | 1.661164355 |  |  |
| Tumor |  | 0.960656612 | 0.486108968 |  | 0.033274164 | 0.634946226 |  |  |
| Tumor |  | 0.113051379 | 0.224853956 |  | 0.94213985 | 1.004349663 |  |  |
| Tumor |  | 0.464584612 | 0.270449354 |  | 0.271378426 | 1.841684361 |  |  |
| Tumor |  | 2.04934892 | 0.08690803 |  | 0.084482295 | 0.880026343 |  |  |
| Tumor |  | 3.899602384 | 0.409994669 |  | 0.044590134 | 0.163022104 |  |  |
| Tumor |  | 0.696732664 | 0.226126999 |  | 0.070588933 | 0.957104225 |  |  |
| Tumor |  | 0.817298057 | 0.553156344 |  | 0.265368952 | 0.40308404 |  |  |
| Tumor |  | 0.476770445 | 0.500041625 |  | 0.134230233 | 1.896895839 |  |  |
| Tumor |  | 0.822878684 | 0.147373097 |  | 0.17956146 | 0.616647531 |  |  |
| Tumor |  | 0.424864419 | 0.305323132 |  | 0.100081334 | 0.59576411 |  |  |
| Tumor |  | 1.479606277 | 0.560817979 |  | 0.082125363 | 0.845789928 |  |  |
| Tumor |  | 0.392729405 | 0.394807936 |  | 0.506663712 | 1.079137722 |  |  |
| Tumor |  | 2.813352542 | 0.209153876 |  | 0.020757011 | 1.871624704 |  |  |
| Tumor |  | 1.762554088 | 0.518990004 |  | 2.666100175 | 0.501742013 |  |  |
| Tumor |  | 0.921021664 | 2.246577882 |  | 0.0094713 | 1.170081716 |  |  |
| Tumor |  | 0.441662357 | 0.31129508 |  | 0.495880403 | 1.914438618 |  |  |
| Tumor |  | 2.141244149 | 0.319658119 |  | 0.221356089 | 1.019402257 |  |  |
| Tumor |  | 0.744774613 | 0.206505929 |  | 0.024005476 | 0.809143095 |  |  |
| Tumor |  | 0.246993265 | 0.21041828 |  | 1.405352253 | 0.618603577 |  |  |
| Tumor |  | 1.236434503 | 0.187682299 |  | 0.065327441 | 1.041020272 |  |  |
| Tumor |  | 0.466534292 | 0.757380838 |  | 0.159381442 | 3.069526989 |  |  |
| Tumor |  | 0.858547632 | 0.28997366 |  | 0.044439392 | 2.208434243 |  |  |
| Tumor |  | 0.721275477 | 1.351789062 |  | 0.082909275 | 0.566588386 |  |  |
| Tumor |  | 0.53681192 | 0.167184484 |  | 0.194797155 | 0.360143932 |  |  |
| Tumor |  | 1.090753602 | 0.042481414 |  | 0.021927906 | 0.969170472 |  |  |
| Tumor |  | 0.176099853 | 0.231510427 |  | 0.132328562 | 1.268767939 |  |  |
| Tumor |  | 0.547737989 | 0.149386969 |  | 0.400917957 | 0.314986075 |  |  |
| Tumor |  | 0.113030365 | 0.149411115 |  | 0.113564768 | 1.877667377 |  |  |
| Tumor |  | 0.37926712 | 0.549398999 |  | 0.010829047 | 0.735311895 |  |  |
| Tumor |  | 0.198671095 | 1.549162211 |  | 0.543006941 | 0.498065759 |  |  |
| Tumor |  | 0.347852083 | 0.521145138 |  | 0.582007982 | 0.505401285 |  |  |
| Tumor |  | 4.323251868 | 0.243878325 |  | 0.169344826 | 1.398693376 |  |  |
| Tumor |  | 1.29190522 | 0.20967681 |  | 0.717738431 | 2.371913314 |  |  |
| Tumor |  | 0.330143645 | 0.18293669 |  | 0.111562017 | 0.70283957 |  |  |
| Tumor |  | 1.021999127 | 0.232845813 |  | 0.448621073 | 0.996331513 |  |  |
| Tumor |  | 0.864106184 | 0.244381336 |  | 0.011409002 | 1.016854802 |  |  |
| Tumor |  | 0.264654439 | 0.056931936 |  | 0.044455481 | 1.55138143 |  |  |
| Tumor |  | 0.450387894 | 0.41227139 |  | 0.821347486 | 0.851659957 |  |  |
| Tumor |  | 0.436496455 | 0.316653377 |  | 0.111980056 | 0.427743868 |  |  |
| Tumor |  | 0.592998649 | 0.175550541 |  | 0.193108008 | 0.782075771 |  |  |
| Tumor |  | 0.187010035 | 0.290742627 |  | 0.060316829 | 2.265502209 |  |  |
| Tumor |  | 0.279788175 | 1.692056617 |  | 0.054883338 | 0.963295409 |  |  |
| Tumor |  | 0.783496353 | 0.219823964 |  | 0.076271698 | 1.756517868 |  |  |
| Tumor |  | 0.916282916 | 0.212038667 |  | 0.040352037 | 1.329742873 |  |  |
| Tumor |  | 6.077091175 | 0.146065915 |  | 0.426993047 | 1.635080356 |  |  |
| Tumor |  | 0.156652456 | 0.914811995 |  | 0.060318119 | 0.820555161 |  |  |
| Tumor |  | 0.797069826 | 0.099679566 |  | 0.077113309 | 1.262840232 |  |  |
| Tumor |  | 0.292552548 | 0.236232565 |  | 0.227330576 | 1.34671175 |  |  |
| Tumor |  | 2.377252282 | 0.219320054 |  | 0.948416668 | 0.981192182 |  |  |
| Tumor |  | 1.281998264 | 0.142799596 |  | 1.846279668 | 1.382327769 |  |  |
| Tumor |  | 0.745166482 | 0.300391158 |  | 0.090933167 | 1.026314776 |  |  |
| Tumor |  | 0.54010116 | 0.078034808 |  | 0.140233959 | 0.070175084 |  |  |
| Tumor |  | 3.086712408 | 0.407598456 |  | 0.665257033 | 1.460254753 |  |  |
| Tumor |  | 2.554383571 | 0.096719425 |  | 0.024197827 | 1.098491637 |  |  |
| Tumor |  | 0.093873488 | 0.380889955 |  |  | 0.852982024 |  |  |
| Tumor |  | 1.497535215 | 0.036062328 |  |  | 0.324751519 |  |  |
| Tumor |  | 0.214628843 | 0.170450971 |  |  | 0.860373591 |  |  |
| Tumor |  | 1.627264338 | 1.125513264 |  |  | 0.97238231 |  |  |
| Tumor |  | 0.249512627 | 0.184752981 |  |  | 1.102517592 |  |  |
| Tumor |  | 0.306842327 | 0.91430564 |  |  | 1.108385625 |  |  |
| Tumor |  | 5.55622382 | 0.042072119 |  |  | 1.565389299 |  |  |
| Tumor |  | 5.269360816 | 0.929459936 |  |  | 0.392015724 |  |  |
| Tumor |  | 0.47562592 | 0.475294116 |  |  | 1.727307909 |  |  |
| Tumor |  | 2.107598186 | 0.056407473 |  |  | 0.437748591 |  |  |
| Tumor |  | 0.217497361 | 0.303501407 |  |  | 0.650031434 |  |  |
| Tumor |  | 0.303281232 | 0.715867573 |  |  | 1.338410006 |  |  |
| Tumor |  | 3.719065811 | 0.976019631 |  |  | 1.850785804 |  |  |
| Tumor |  | 0.207844269 | 0.722377268 |  |  | 1.230147619 |  |  |
| Tumor |  | 0.536971805 | 0.152774751 |  |  | 1.113636536 |  |  |
| Tumor |  | 0.283979214 | 0.539026248 |  |  | 0.840569343 |  |  |
| Tumor |  | 0.180882161 | 0.301275516 |  |  | 1.079775775 |  |  |
| Tumor |  | 0.817631261 | 0.097920711 |  |  | 1.067321699 |  |  |
| Tumor |  | 0.522215921 | 0.30662115 |  |  | 0.680189585 |  |  |
| Tumor |  | 0.420485028 | 0.299382718 |  |  | 0.711814473 |  |  |
| Tumor |  | 0.643637092 | 0.33568169 |  |  | 1.291277844 |  |  |
| Tumor |  | 0.450469937 | 0.236300426 |  |  | 1.917448556 |  |  |
| Tumor |  | 5.556203972 | 0.359711227 |  |  | 0.664603654 |  |  |
| Tumor |  | 5.629293738 | 0.072797353 |  |  | 1.305916434 |  |  |
| Tumor |  | 1.34312348 | 1.009522678 |  |  | 2.36608735 |  |  |
| Tumor |  | 0.443575583 | 0.296224925 |  |  | 0.823518702 |  |  |
| Tumor |  | 2.581176597 | 0.192070672 |  |  | 1.884906638 |  |  |
| Tumor |  | 0.969421241 | 0.736157646 |  |  | 0.897160326 |  |  |
| Tumor |  | 1.489700821 | 0.131038727 |  |  | 1.0928591 |  |  |
| Tumor |  | 0.450911542 | 0.141035749 |  |  | 1.021016553 |  |  |
| Tumor |  | 0.263292514 | 0.232775498 |  |  | 0.362247465 |  |  |
| Tumor |  | 1.480176673 | 0.313534552 |  |  | 0.612777513 |  |  |
| Tumor |  | 1.309341518 | 0.553152134 |  |  | 0.773049889 |  |  |
| Tumor |  | 0.521820922 | 0.322168216 |  |  | 0.99517629 |  |  |
| Tumor |  | 0.578769072 | 0.218443079 |  |  | 1.343105645 |  |  |
| Tumor |  | 0.270033617 | 0.106920362 |  |  | 0.736525405 |  |  |
| Tumor |  | 0.195920217 | 0.186432699 |  |  | 0.979840143 |  |  |
| Tumor |  | 1.165551782 | 1.031289103 |  |  | 0.2955148 |  |  |
| Tumor |  | 0.653419254 | 0.395023355 |  |  | 1.316622274 |  |  |
| Tumor |  | 1.119538418 | 0.34847222 |  |  | 0.25600664 |  |  |
| Tumor |  | 1.019624462 | 0.204350947 |  |  | 0.900184129 |  |  |
| Tumor |  | 0.549989584 | 0.302673234 |  |  | 0.662059047 |  |  |
| Tumor |  | 2.064831138 | 1.346346518 |  |  | 0.439012049 |  |  |
| Tumor |  | 0.472943385 | 0.852717545 |  |  | 1.225223276 |  |  |
| Tumor |  | 0.08600295 | 0.109250902 |  |  | 2.326056595 |  |  |
| Tumor |  | 0.703996739 | 0.255455604 |  |  | 0.870537489 |  |  |
| Tumor |  | 0.860062704 | 0.182359467 |  |  | 0.944737877 |  |  |
| Tumor |  | 0.967209547 | 0.475480068 |  |  | 0.936667735 |  |  |
| Tumor |  | 0.334316503 | 0.114860869 |  |  | 1.244440987 |  |  |
| Tumor |  | 0.489541761 | 0.163340406 |  |  | 0.606208441 |  |  |
| Tumor |  | 4.375521623 | 0.295707797 |  |  | 1.774031929 |  |  |
| Tumor |  | 0.515605294 | 0.196059453 |  |  | 0.647359875 |  |  |
| Tumor |  | 1.56158007 | 0.149048479 |  |  | 1.025452803 |  |  |
| Tumor |  | 0.812957647 | 0.17704557 |  |  | 0.620766905 |  |  |
| Tumor |  | 1.333288186 | 0.35279307 |  |  | 0.560181011 |  |  |
| Tumor |  | 0.50828203 | 0.045411464 |  |  | 1.540388136 |  |  |
| Tumor |  | 1.316673244 | 0.656396121 |  |  | 2.400970038 |  |  |
| Tumor |  | 1.310901918 | 0.257827687 |  |  | 0.987662047 |  |  |
| Tumor |  | 0.103168298 | 0.374927133 |  |  | 0.929438962 |  |  |
| Tumor |  | 0.278893174 | 0.402953536 |  |  | 0.517071606 |  |  |
| Tumor |  | 0.727344707 | 0.373194572 |  |  | 1.289068312 |  |  |
| Tumor |  | 1.019941828 | 0.899647175 |  |  | 1.708667215 |  |  |
| Tumor |  | 0.305653552 | 0.808818435 |  |  | 0.551034865 |  |  |
| Tumor |  | 0.280499545 | 0.573808817 |  |  | 3.131193654 |  |  |
| Tumor |  | 0.24297871 | 0.38689952 |  |  | 0.181679624 |  |  |
| Tumor |  | 0.280797856 | 0.665196557 |  |  | 0.901065624 |  |  |
| Tumor |  | 0.078360964 | 0.237407564 |  |  | 0.278063955 |  |  |
| Tumor |  | 0.438304102 | 0.325093427 |  |  | 0.48711492 |  |  |
| Tumor |  | 0.602351078 | 0.555223795 |  |  | 0.973461395 |  |  |
| Tumor |  | 0.943360216 | 0.126075476 |  |  | 0.156887921 |  |  |
| Tumor |  | 0.4054401 | 0.305962102 |  |  | 1.036006155 |  |  |
| Tumor |  | 0.528919699 | 0.444586295 |  |  | 1.375925856 |  |  |
| Tumor |  | 0.670478307 | 1.021395132 |  |  | 1.14516099 |  |  |
| Tumor |  | 1.742442726 | 0.456625542 |  |  | 0.550223925 |  |  |
| Tumor |  | 6.325696424 | 0.104170093 |  |  | 2.614254313 |  |  |
| Tumor |  | 0.644246525 | 0.808132791 |  |  | 0.381131472 |  |  |
| Tumor |  | 0.193322272 | 1.594266707 |  |  | 0.617659721 |  |  |
| Tumor |  | 0.623210327 | 0.118436706 |  |  | 1.519132579 |  |  |
| Tumor |  | 0.815466815 | 0.185962243 |  |  | 1.471284382 |  |  |
| Tumor |  | 0.59326028 | 0.276581665 |  |  | 0.709705127 |  |  |
| Tumor |  | 0.147068906 | 0.148649483 |  |  | 0.81905565 |  |  |
| Tumor |  | 1.58047134 | 0.481232467 |  |  | 1.167641067 |  |  |
| Tumor |  | 2.892634637 | 1.00131118 |  |  | 1.418443176 |  |  |
| Tumor |  | 0.038892839 | 0.356798161 |  |  | 0.844267062 |  |  |
| Tumor |  | 0.799287401 | 0.239609123 |  |  | 0.794502497 |  |  |
| Tumor |  | 0.621463588 | 0.453005794 |  |  | 2.452187415 |  |  |
| Tumor |  | 0.988873005 | 0.767781664 |  |  | 0.541199312 |  |  |
| Tumor |  | 2.009877148 | 0.554555531 |  |  | 1.761743579 |  |  |
| Tumor |  | 0.618674839 | 0.55553571 |  |  | 1.043269243 |  |  |
| Tumor |  | 0.979063925 | 0.184098169 |  |  | 0.832765378 |  |  |
| Tumor |  | 1.182145993 | 0.412202328 |  |  | 0.811189971 |  |  |
| Tumor |  | 2.598946761 | 0.489382963 |  |  | 2.987256842 |  |  |
| Tumor |  | 2.316805053 | 0.086141462 |  |  | 0.998443816 |  |  |
| Tumor |  | 1.62101197 | 0.169387343 |  |  | 0.522598035 |  |  |
| Tumor |  | 0.860025431 | 0.311637145 |  |  | 1.259124456 |  |  |
| Tumor |  | 0.531974112 | 0.386117968 |  |  | 0.521543992 |  |  |
| Tumor |  | 0.757534863 | 0.192650688 |  |  | 0.959125398 |  |  |
| Tumor |  | 1.979216583 | 0.250106196 |  |  | 2.37134933 |  |  |
| Tumor |  | 0.410344383 | 0.607458399 |  |  | 0.535188895 |  |  |
| Tumor |  | 0.687228945 | 0.761326364 |  |  | 1.572581689 |  |  |
| Tumor |  | 1.437010542 | 0.135801298 |  |  | 1.285416284 |  |  |
| Tumor |  | 0.549984865 | 1.161613505 |  |  | 0.777608815 |  |  |
| Tumor |  | 0.2476789 | 0.401147342 |  |  | 1.30937409 |  |  |
| Tumor |  | 0.540621225 | 0.745454452 |  |  | 0.525712753 |  |  |
| Tumor |  | 0.043154386 | 0.128674225 |  |  | 1.611324784 |  |  |
| Tumor |  | 0.127456712 | 0.585906255 |  |  | 1.293722922 |  |  |
| Tumor |  | 0.231049299 | 0.186882957 |  |  | 1.137648918 |  |  |
| Tumor |  | 0.888487435 | 0.187037312 |  |  | 1.739666606 |  |  |
| Tumor |  | 0.250274312 | 0.557441976 |  |  | 1.120426139 |  |  |
| Tumor |  | 1.821964515 | 0.835938756 |  |  | 0.930306218 |  |  |
| Tumor |  | 1.824706572 | 1.902548526 |  |  | 0.880271464 |  |  |
| Tumor |  | 0.720213156 | 0.366385061 |  |  | 2.010628868 |  |  |
| Tumor |  | 0.583893637 | 0.489388706 |  |  | 0.753801931 |  |  |
| Tumor |  | 3.445115457 | 0.208240861 |  |  | 1.454005371 |  |  |
| Tumor |  | 0.553693788 | 0.16608 |  |  | 0.410966145 |  |  |
| Tumor |  | 1.131785203 | 0.619844512 |  |  | 1.116276511 |  |  |
| Tumor |  | 0.971932453 | 0.113187399 |  |  | 1.165547872 |  |  |
| Tumor |  | 1.268773469 | 0.160896026 |  |  | 1.042117136 |  |  |
| Tumor |  |  | 0.11715319 |  |  | 1.219136402 |  |  |
| Tumor |  |  | 0.336246271 |  |  | 2.028849 |  |  |
| Tumor |  |  | 0.281017422 |  |  | 0.872944844 |  |  |
| Tumor |  |  | 0.204267283 |  |  | 1.450655367 |  |  |
| Tumor |  |  | 0.085942889 |  |  | 0.828555109 |  |  |
| Tumor |  |  | 0.492520407 |  |  | 0.938920185 |  |  |
| Tumor |  |  | 0.37381422 |  |  | 0.539613295 |  |  |
| Tumor |  |  | 0.222354916 |  |  | 1.172919351 |  |  |
| Tumor |  |  | 0.517714374 |  |  | 1.631965561 |  |  |
| Tumor |  |  | 0.213117588 |  |  | 0.909705474 |  |  |
| Tumor |  |  | 0.321654292 |  |  | 2.024676428 |  |  |
| Tumor |  |  | 0.128033071 |  |  | 1.122999913 |  |  |
| Tumor |  |  | 0.553306676 |  |  | 1.582984403 |  |  |
| Tumor |  |  | 0.47690694 |  |  | 1.965046363 |  |  |
| Tumor |  |  | 0.520870323 |  |  | 1.371199904 |  |  |
| Tumor |  |  | 0.156055652 |  |  | 0.653392331 |  |  |
| Tumor |  |  | 0.563516815 |  |  | 1.056693849 |  |  |
| Tumor |  |  | 0.251083633 |  |  | 0.340887802 |  |  |
| Tumor |  |  | 0.181070879 |  |  | 0.609396023 |  |  |
| Tumor |  |  | 0.456324992 |  |  | 1.395652659 |  |  |
| Tumor |  |  | 0.350034354 |  |  | 0.792405302 |  |  |
| Tumor |  |  | 0.09752594 |  |  | 0.675306973 |  |  |
| Tumor |  |  | 0.681906812 |  |  | 0.686311253 |  |  |
| Tumor |  |  | 0.190425303 |  |  | 1.019422193 |  |  |
| Tumor |  |  | 0.286256187 |  |  | 1.050417116 |  |  |
| Tumor |  |  | 0.556862597 |  |  | 1.915540432 |  |  |
| Tumor |  |  | 0.477845316 |  |  | 1.493439125 |  |  |
| Tumor |  |  | 1.132560688 |  |  | 0.833475978 |  |  |
| Tumor |  |  | 0.194390052 |  |  | 1.887876964 |  |  |
| Tumor |  |  | 0.270537998 |  |  | 1.910246536 |  |  |
| Tumor |  |  | 0.188294622 |  |  | 1.858729942 |  |  |
| Tumor |  |  | 0.119679837 |  |  | 0.974176945 |  |  |
| Tumor |  |  | 0.287044045 |  |  | 1.06296874 |  |  |
| Tumor |  |  | 0.062144108 |  |  | 1.283621579 |  |  |
| Tumor |  |  | 0.111172776 |  |  | 1.458281033 |  |  |
| Tumor |  |  | 0.341855111 |  |  | 0.775918659 |  |  |
| Tumor |  |  | 0.445686608 |  |  | 1.983512535 |  |  |
| Tumor |  |  | 0.225356971 |  |  | 0.591013696 |  |  |
| Tumor |  |  | 0.477349544 |  |  | 1.348964834 |  |  |
| Tumor |  |  | 0.611681218 |  |  | 1.803057173 |  |  |
| Tumor |  |  | 0.472303368 |  |  | 0.588092003 |  |  |
| Tumor |  |  | 0.191029786 |  |  | 0.752693606 |  |  |
| Tumor |  |  | 0.110189408 |  |  | 0.426462961 |  |  |
| Tumor |  |  | 0.601823362 |  |  | 0.331917515 |  |  |
| Tumor |  |  | 0.1516568 |  |  | 1.995114899 |  |  |
| Tumor |  |  | 0.133709214 |  |  | 0.685150169 |  |  |
| Tumor |  |  | 0.252689314 |  |  | 1.785136963 |  |  |
| Tumor |  |  | 0.082311886 |  |  | 1.433968488 |  |  |
| Tumor |  |  | 0.20669376 |  |  | 1.759631228 |  |  |
| Tumor |  |  | 0.194223629 |  |  | 1.295622267 |  |  |
| Tumor |  |  | 0.407528546 |  |  | 0.424349516 |  |  |
| Tumor |  |  | 0.499371445 |  |  | 1.218749413 |  |  |
| Tumor |  |  | 0.017703973 |  |  | 0.679669554 |  |  |
| Tumor |  |  | 0.28503147 |  |  | 1.751952711 |  |  |
| Tumor |  |  | 0.628792413 |  |  | 0.763684263 |  |  |
| Tumor |  |  | 0.226316126 |  |  | 1.59991858 |  |  |
| Tumor |  |  | 0.168685494 |  |  | 0.610703938 |  |  |
| Tumor |  |  | 0.447307074 |  |  | 0.705968064 |  |  |
| Tumor |  |  | 0.461076834 |  |  | 1.604642259 |  |  |
| Tumor |  |  | 0.163085419 |  |  | 0.381065092 |  |  |
| Tumor |  |  | 0.126325722 |  |  | 1.051918801 |  |  |
| Tumor |  |  | 1.839932117 |  |  | 0.596804709 |  |  |
| Tumor |  |  | 0.403699846 |  |  | 1.427535721 |  |  |
| Tumor |  |  | 0.528084931 |  |  | 1.36534271 |  |  |
| Tumor |  |  | 0.367945318 |  |  | 0.56206939 |  |  |
| Tumor |  |  | 0.33925672 |  |  | 0.976326104 |  |  |
| Tumor |  |  | 0.426810318 |  |  | 0.472942862 |  |  |
| Tumor |  |  | 0.205135643 |  |  | 1.629715693 |  |  |
| Tumor |  |  | 0.298093914 |  |  | 1.181773625 |  |  |
| Tumor |  |  | 1.312061796 |  |  | 0.361012827 |  |  |
| Tumor |  |  | 0.315231253 |  |  | 0.302854577 |  |  |
| Tumor |  |  | 0.324296956 |  |  | 0.918679987 |  |  |
| Tumor |  |  | 0.763086172 |  |  | 1.036636884 |  |  |
| Tumor |  |  | 0.231402436 |  |  | 2.014971782 |  |  |
| Tumor |  |  | 0.331589611 |  |  | 0.411460154 |  |  |
| Tumor |  |  | 1.26529862 |  |  | 0.341256462 |  |  |
| Tumor |  |  | 0.308379063 |  |  |  |  |  |
| Tumor |  |  | 0.280416215 |  |  |  |  |  |
| Tumor |  |  | 0.403352361 |  |  |  |  |  |
| Tumor |  |  | 0.935168294 |  |  |  |  |  |
| Tumor |  |  | 0.85561656 |  |  |  |  |  |
| Tumor |  |  | 0.066197018 |  |  |  |  |  |
| Tumor |  |  | 0.26508122 |  |  |  |  |  |
| Tumor |  |  | 0.204380892 |  |  |  |  |  |
| Tumor |  |  | 0.373140208 |  |  |  |  |  |
| Tumor |  |  | 0.100153561 |  |  |  |  |  |
| Tumor |  |  | 0.212260304 |  |  |  |  |  |
| Tumor |  |  | 0.573073173 |  |  |  |  |  |
| Tumor |  |  | 0.211712689 |  |  |  |  |  |
| Tumor |  |  | 0.119779295 |  |  |  |  |  |
| Tumor |  |  | 0.124166291 |  |  |  |  |  |
| Tumor |  |  | 0.110248671 |  |  |  |  |  |
| Tumor |  |  | 0.565166561 |  |  |  |  |  |
| Tumor |  |  | 0.178964391 |  |  |  |  |  |
| Tumor |  |  | 0.150241683 |  |  |  |  |  |
| Tumor |  |  | 0.180071598 |  |  |  |  |  |
| Tumor |  |  | 0.390086008 |  |  |  |  |  |
| Tumor |  |  | 0.235883142 |  |  |  |  |  |
| Tumor |  |  | 0.256756376 |  |  |  |  |  |
| Tumor |  |  | 0.07035309 |  |  |  |  |  |
| Tumor |  |  | 0.2121549 |  |  |  |  |  |
| Tumor |  |  | 0.223217838 |  |  |  |  |  |
| Tumor |  |  | 0.244655572 |  |  |  |  |  |
| Tumor |  |  | 0.363326374 |  |  |  |  |  |
| Tumor |  |  | 0.381974585 |  |  |  |  |  |
| Tumor |  |  | 0.437808327 |  |  |  |  |  |
| Tumor |  |  | 1.164255897 |  |  |  |  |  |
| Tumor |  |  | 0.131569703 |  |  |  |  |  |
| Tumor |  |  | 0.312406008 |  |  |  |  |  |
| Tumor |  |  | 0.45985661 |  |  |  |  |  |
| Tumor |  |  | 0.116720428 |  |  |  |  |  |
| Tumor |  |  | 0.392816947 |  |  |  |  |  |
| Tumor |  |  | 0.362763633 |  |  |  |  |  |
| Tumor |  |  | 0.369714608 |  |  |  |  |  |
| Tumor |  |  | 0.358861736 |  |  |  |  |  |
| Tumor |  |  | 0.319874084 |  |  |  |  |  |
| Tumor |  |  | 0.965089 |  |  |  |  |  |
| Tumor |  |  | 0.183704936 |  |  |  |  |  |
| Tumor |  |  | 0.47148986 |  |  |  |  |  |
| Tumor |  |  | 0.149254677 |  |  |  |  |  |
| Tumor |  |  | 0.574374237 |  |  |  |  |  |
|  |  |  |  |  |  |  |  |  |
| Normal | 0.014009596 | 0.246332133 | 0.131291539 | 0.148885124 | 0.132018977 | 0.833164811 | 0.009742015 | 0.616819361 |
| Normal | 0.155521025 | 1.948092883 | 0.074221636 | 0.126888836 | 0.042900445 | 0.446583391 | 0.033772495 | 0.924933262 |
| Normal | 0.06392096 | 0.257534294 | 0.094542072 | 0.113317272 | 0.05574507 | 1.071164565 | 0.028989316 | 0.791611113 |
| Normal | 0.034781806 | 1.197770694 | 0.092929712 | 0.113625153 | 0.046991259 | 0.695072301 |  | 0.73615179 |
| Normal | 0.028209636 | 3.82258844 | 0.268100417 | 0.048356571 | 0.060477487 | 0.300019824 |  | 0.57300704 |
| Normal | 0.288368485 | 3.813419422 | 0.138718889 | 0.063987406 | 0.048171805 | 0.368524411 |  | 0.483126898 |
| Normal | 0.044555538 | 0.347483655 | 0.124899764 | 0.097911207 | 0.084515531 | 0.31471569 |  | 0.826363922 |
| Normal | 0.056079413 | 0.141562212 | 0.07411257 | 0.032993535 | 0.170307668 | 0.663249211 |  | 0.643135824 |
| Normal | 0 | 0.795772695 | 0.103967791 | 0.102347063 | 0.05900221 | 0.477178083 |  | 0.671841894 |
| Normal | 0.340411868 | 1.518410826 | 0.044219836 | 0.030448985 | 0.091824115 | 0.141321999 |  | 0.801930449 |
| Normal | 0.087677688 | 2.218237837 | 0.108448685 | 0.136866552 | 0.20646002 | 0.398177864 |  | 0.799792702 |
| Normal | 0.151742332 | 0.850010411 | 0.481552972 | 0.100693022 | 0.032676089 | 0.285105109 |  | 0.62756756 |
| Normal | 1.266519111 | 0.322821195 | 0.1092133 | 0.175949644 | 0.022438293 | 0.436182865 |  | 0.5938072 |
| Normal | 0.686755002 | 0.339799101 | 0.045713522 | 0.052674952 | 0.128736757 | 0.086455841 |  | 0.717114487 |
| Normal | 0.446163153 | 0.4535907 | 0.08421665 | 0.150647486 | 0.117359162 | 1.485857683 |  | 0.689510374 |
| Normal | 0.101275586 | 0.379832374 | 0.120341535 | 0.035774543 | 0.037805914 | 0.503920524 |  | 0.925187683 |
| Normal | 0.074404088 | 0.390345619 | 0.107487746 | 0.073285216 | 0.028702247 | 0.309381395 |  | 0.586912505 |
| Normal | 0.341871351 | 1.142983034 | 0.209556289 | 0.163910575 | 0.03164283 | 0.437201696 |  | 0.548509234 |
| Normal | 0.101130559 | 0.757665131 | 0.166022673 | 0.063995278 | 0.046972752 | 0.358935847 |  | 0.609342596 |
| Normal | 0.172661857 |  | 0.055308774 | 0.061489908 | 0.186149509 | 0.068992039 |  | 0.524547985 |
| Normal | 0.110064209 |  | 0.130954382 | 0.099895892 | 0.098905607 | 0.315022753 |  | 0.830272378 |
| Normal | 0.085702236 |  | 0.889941925 | 0.054913597 | 0.063173285 | 0.214597589 |  | 1.01223159 |
| Normal | 0.205643117 |  | 0.162773515 | 0.233471399 | 0.073038594 | 0.268438302 |  | 0.827948909 |
| Normal | 0.128335704 |  | 0.109262405 | 0.107787616 | 0.045172254 | 0.090890296 |  | 0.550113809 |
| Normal | 0.079994663 |  | 0.045744972 |  | 0.058904221 | 0.452459575 |  | 0.737382677 |
| Normal | 0.223630135 |  | 1.500518849 |  | 0.026173477 | 0.383356822 |  | 0.677930928 |
| Normal | 0.216075229 |  | 0.055798576 |  | 0.034289774 | 0.628688528 |  | 0.930654765 |
| Normal | 0.029451713 |  | 0.102670753 |  | 0.010100781 | 0.124832769 |  | 0.877557524 |
| Normal | 0.217350222 |  | 0.160950173 |  | 0.050843439 | 0.119180796 |  | 0.864435561 |
| Normal | 0.03608737 |  | 0.025407269 |  | 0.058064143 | 0.255066725 |  | 0.789534489 |
| Normal | 0.013803214 |  | 0.163928833 |  | 0.018259186 | 0.609181471 |  | 1.073790529 |
| Normal | 0.107335675 |  | 0.055557837 |  | 0.032369617 | 0.45820045 |  | 1.03095559 |
| Normal | 0.035761811 |  | 0.063783161 |  |  | 1.030068527 |  | 0.823282645 |
| Normal | 0.053626568 |  | 0.040107173 |  |  | 1.458119736 |  | 0.503985236 |
| Normal | 0.068539418 |  | 0.042272649 |  |  | 0.328633775 |  | 0.806651048 |
| Normal | 0.043564413 |  | 0.095736428 |  |  | 1.049024076 |  | 0.778374592 |
| Normal | 0.16185454 |  | 0.16206049 |  |  | 0.22682815 |  | 0.806236419 |
| Normal | 0.023449246 |  | 0.032627993 |  |  | 0.149620082 |  | 0.559271328 |
| Normal | 0.290949129 |  | 0.128982364 |  |  | 0.368581005 |  | 0.750369171 |
| Normal | 0.007756302 |  | 0.115794628 |  |  | 0.435390579 |  | 0.82145634 |
| Normal | 0.105155838 |  | 0.233517418 |  |  | 0.212576398 |  | 0.776771633 |
| Normal | 0.769677051 |  | 0.215731352 |  |  | 0.806651652 |  | 0.857295727 |
| Normal | 0.036533816 |  | 0.10685134 |  |  | 0.660141015 |  | 0.886177332 |
| Normal | 0.054613293 |  | 0.249698513 |  |  | 0.509160472 |  | 0.518124967 |
| Normal | 0.369267243 |  | 0.086373811 |  |  | 0.230378359 |  | 0.573006239 |
| Normal | 0.081879405 |  | 0.077918548 |  |  | 0.301621837 |  | 0.707323266 |
| Normal | 0.038540046 |  | 0.044422581 |  |  | 1.078560275 |  | 0.886840695 |
| Normal | 0.046749248 |  | 0.070604343 |  |  | 0.225800177 |  | 0.709371838 |
| Normal | 0.237920493 |  | 0.064015793 |  |  | 0.757782205 |  | 0.781260228 |
| Normal | 0.290488693 |  | 0.079318961 |  |  | 0.1300545 |  | 0.922158725 |
| Normal | 0.05632672 |  | 0.058890656 |  |  | 1.050866255 |  | 0.819793399 |
| Normal | 0.065784985 |  | 0.049699448 |  |  |  |  | 0.747622185 |
| Normal | 0.127906092 |  | 0.058788504 |  |  |  |  | 0.711166572 |
| Normal | 0.332759712 |  | 0.085655665 |  |  |  |  | 0.673232094 |
| Normal | 0.02206763 |  | 0.245547849 |  |  |  |  | 0.713075403 |
| Normal | 0.049138202 |  | 0.078954655 |  |  |  |  | 0.569543088 |
| Normal | 0.042916841 |  | 0.20197122 |  |  |  |  | 0.568478606 |
| Normal | 0.176195861 |  | 0.150901496 |  |  |  |  | 0.785365224 |
| Normal | 0.021181734 |  | 0.070261753 |  |  |  |  | 0.750774884 |
| Normal | 0.041462146 |  | 0.04451192 |  |  |  |  | 0.639713421 |
| Normal | 0.069612798 |  | 0.019939183 |  |  |  |  | 0.774633036 |
| Normal | 0.114138895 |  | 0.145511922 |  |  |  |  | 0.62803716 |
| Normal | 0.048405755 |  | 0.04206643 |  |  |  |  | 0.783399543 |
| Normal | 1.013800963 |  | 0.049357807 |  |  |  |  | 0.804963814 |
| Normal | 0.104504794 |  | 0.08359337 |  |  |  |  | 0.438604922 |
| Normal | 0.251835655 |  | 0.048585756 |  |  |  |  | 0.611069214 |
| Normal | 0.073053489 |  | 0.099214639 |  |  |  |  | 0.591141203 |
| Normal | 0.050802541 |  | 0.062702642 |  |  |  |  | 0.732871459 |
| Normal | 0.007090495 |  | 0.132472703 |  |  |  |  | 0.584844033 |
| Normal | 0.04557345 |  | 0.064946848 |  |  |  |  | 0.728263526 |
| Normal | 0.862415921 |  | 0.077242003 |  |  |  |  | 1.030613016 |
| Normal | 0.047961364 |  | 0.172224649 |  |  |  |  | 0.702454149 |
| Normal | 0.014526692 |  |  |  |  |  |  | 0.722770545 |
| Normal | 0.030447435 |  |  |  |  |  |  | 0.965968198 |
| Normal | 0.139963843 |  |  |  |  |  |  | 0.735797564 |
| Normal | 0 |  |  |  |  |  |  | 0.857594068 |
| Normal | 0.09641961 |  |  |  |  |  |  | 0.780383638 |
| Normal | 0.070349715 |  |  |  |  |  |  | 1.089580525 |
| Normal | 0.97753594 |  |  |  |  |  |  | 0.643935174 |
| Normal | 0.047645006 |  |  |  |  |  |  | 0.775112815 |
| Normal | 0.056935198 |  |  |  |  |  |  | 0.743594439 |
| Normal | 0.082483062 |  |  |  |  |  |  | 0.703612771 |
| Normal | 0.135798563 |  |  |  |  |  |  | 0.425958509 |
| Normal | 0.039457226 |  |  |  |  |  |  | 1.202749295 |
| Normal | 0.041790051 |  |  |  |  |  |  | 0.744779456 |
| Normal | 0.134042153 |  |  |  |  |  |  | 0.654919671 |
| Normal | 0.08730838 |  |  |  |  |  |  | 0.738270147 |
| Normal | 0.183859963 |  |  |  |  |  |  | 0.67565082 |
| Normal | 0.056107638 |  |  |  |  |  |  | 0.430511831 |
| Normal | 0.217492549 |  |  |  |  |  |  | 0.826785812 |
| Normal | 0.066895414 |  |  |  |  |  |  | 0.729849412 |
| Normal | 0.039190513 |  |  |  |  |  |  | 0.702628304 |
| Normal | 0.12411186 |  |  |  |  |  |  | 0.87253613 |
| Normal | 0.159323695 |  |  |  |  |  |  | 0.740862391 |
| Normal | 0.029697436 |  |  |  |  |  |  | 0.640473119 |
| Normal | 0.036057962 |  |  |  |  |  |  | 0.580440081 |
| Normal | 0.392051063 |  |  |  |  |  |  | 0.602850133 |
| Normal | 0.062586878 |  |  |  |  |  |  | 0.659502614 |
| Normal | 0.021939455 |  |  |  |  |  |  | 0.63519971 |
| Normal | 0.077793757 |  |  |  |  |  |  | 0.693390652 |
| Normal | 0.019993014 |  |  |  |  |  |  | 0.757121292 |
| Normal | 0.058541171 |  |  |  |  |  |  | 0.821752012 |
| Normal | 0.10042648 |  |  |  |  |  |  | 0.465127432 |
| Normal | 1.590670997 |  |  |  |  |  |  | 0.959463693 |
| Normal | 0.026952598 |  |  |  |  |  |  | 0.79655866 |
| Normal | 0.109001055 |  |  |  |  |  |  | 0.585058315 |
| Normal | 0.077515267 |  |  |  |  |  |  | 1.023888439 |
| Normal | 0.108791246 |  |  |  |  |  |  | 0.649656052 |
| Normal | 0.063422619 |  |  |  |  |  |  | 0.671157057 |
| Normal | 0.188457011 |  |  |  |  |  |  | 0.575688186 |
| Normal | 0.065406857 |  |  |  |  |  |  | 0.653721816 |
| Normal | 0.220279676 |  |  |  |  |  |  | 0.68367646 |
| Normal | 0.118756861 |  |  |  |  |  |  | 0.861790241 |
| Normal | 0.043588307 |  |  |  |  |  |  | 0.781668389 |
| Normal | 0.033455987 |  |  |  |  |  |  | 0.82348643 |
| Normal | 0.07107016 |  |  |  |  |  |  | 0.559089686 |
| Normal | 0.128347214 |  |  |  |  |  |  | 1.087578485 |
| Normal | 0.045137708 |  |  |  |  |  |  | 0.68489517 |
| Normal | 0.033917861 |  |  |  |  |  |  | 0.655691229 |
| Normal | 0.074417612 |  |  |  |  |  |  | 0.705810705 |
| Normal | 0.058219269 |  |  |  |  |  |  | 0.652070675 |
| Normal | 0.055627398 |  |  |  |  |  |  | 0.636817715 |
| Normal | 0.161576994 |  |  |  |  |  |  | 0.936477148 |
| Normal | 0.023906771 |  |  |  |  |  |  | 0.901957556 |
| Normal | 1.0674921 |  |  |  |  |  |  | 0.700091005 |
| Normal | 0.354481725 |  |  |  |  |  |  | 0.952904726 |
| Normal |  |  |  |  |  |  |  | 1.09467697 |
| Normal |  |  |  |  |  |  |  | 0.668452914 |
| Normal |  |  |  |  |  |  |  | 0.699113208 |
| Normal |  |  |  |  |  |  |  | 0.673841641 |
| Normal |  |  |  |  |  |  |  | 0.696583183 |
| Normal |  |  |  |  |  |  |  | 0.677881285 |
| Normal |  |  |  |  |  |  |  | 0.831946669 |
| Normal |  |  |  |  |  |  |  | 0.74743049 |
| Normal |  |  |  |  |  |  |  | 0.685361866 |
| Normal |  |  |  |  |  |  |  | 0.789941481 |
| Normal |  |  |  |  |  |  |  | 0.749665419 |
| Normal |  |  |  |  |  |  |  | 0.657697813 |
| Normal |  |  |  |  |  |  |  | 0.529432646 |
| Normal |  |  |  |  |  |  |  | 0.642008695 |
| Normal |  |  |  |  |  |  |  | 0.911491234 |
| Normal |  |  |  |  |  |  |  | 0.604622086 |
| Normal |  |  |  |  |  |  |  | 0.966932938 |
| Normal |  |  |  |  |  |  |  | 0.655222068 |
| Normal |  |  |  |  |  |  |  | 0.847235321 |
| Normal |  |  |  |  |  |  |  | 0.471161346 |
| Normal |  |  |  |  |  |  |  | 0.611117293 |
| Normal |  |  |  |  |  |  |  | 0.675308462 |
| Normal |  |  |  |  |  |  |  | 0.759417818 |
| Normal |  |  |  |  |  |  |  | 0.735233499 |
| Normal |  |  |  |  |  |  |  | 0.678675181 |
| Normal |  |  |  |  |  |  |  | 0.877043619 |
| Normal |  |  |  |  |  |  |  | 0.739271124 |
| Normal |  |  |  |  |  |  |  | 1.048473774 |
| Normal |  |  |  |  |  |  |  | 0.89077323 |
| Normal |  |  |  |  |  |  |  | 0.659721585 |
| Normal |  |  |  |  |  |  |  | 0.651865178 |
| Normal |  |  |  |  |  |  |  | 0.922137975 |
| Normal |  |  |  |  |  |  |  | 0.731419683 |
| Normal |  |  |  |  |  |  |  | 0.906060029 |
| Normal |  |  |  |  |  |  |  | 0.672023449 |
| Normal |  |  |  |  |  |  |  | 0.478431612 |
| Normal |  |  |  |  |  |  |  | 0.944405479 |
| Normal |  |  |  |  |  |  |  | 1.265604427 |
| Normal |  |  |  |  |  |  |  | 0.867606596 |
| Normal |  |  |  |  |  |  |  |  |
| Normal |  |  |  |  |  |  |  |  |
| Normal |  |  |  |  |  |  |  |  |
| Normal |  |  |  |  |  |  |  |  |
| Normal |  |  |  |  |  |  |  |  |
| Normal |  |  |  |  |  |  |  |  |
| Normal |  |  |  |  |  |  |  |  |
| Normal |  |  |  |  |  |  |  |  |
| Normal |  |  |  |  |  |  |  |  |
| Normal |  |  |  |  |  |  |  |  |
| Normal |  |  |  |  |  |  |  |  |
| Normal |  |  |  |  |  |  |  |  |
| Normal |  |  |  |  |  |  |  |  |
| Normal |  |  |  |  |  |  |  |  |
| Normal |  |  |  |  |  |  |  |  |
| Normal |  |  |  |  |  |  |  |  |
| Normal |  |  |  |  |  |  |  |  |
| Normal |  |  |  |  |  |  |  |  |
| Normal |  |  |  |  |  |  |  |  |
| Normal |  |  |  |  |  |  |  |  |
| Normal |  |  |  |  |  |  |  |  |
| Normal |  |  |  |  |  |  |  |  |
| Normal |  |  |  |  |  |  |  |  |
| Normal |  |  |  |  |  |  |  |  |
| Normal |  |  |  |  |  |  |  |  |
| Normal |  |  |  |  |  |  |  |  |
| Normal |  |  |  |  |  |  |  |  |
| Normal |  |  |  |  |  |  |  |  |
| Normal |  |  |  |  |  |  |  |  |
| Normal |  |  |  |  |  |  |  |  |
| Normal |  |  |  |  |  |  |  |  |
| Normal |  |  |  |  |  |  |  |  |
| Normal |  |  |  |  |  |  |  |  |
| Normal |  |  |  |  |  |  |  |  |
| Normal |  |  |  |  |  |  |  |  |
| Normal |  |  |  |  |  |  |  |  |
| Normal |  |  |  |  |  |  |  |  |
| Normal |  |  |  |  |  |  |  |  |
| Normal |  |  |  |  |  |  |  |  |
| Normal |  |  |  |  |  |  |  |  |
| Normal |  |  |  |  |  |  |  |  |
| Normal |  |  |  |  |  |  |  |  |
| Normal |  |  |  |  |  |  |  |  |
| Normal |  |  |  |  |  |  |  |  |
| Normal |  |  |  |  |  |  |  |  |
| Normal |  |  |  |  |  |  |  |  |
| Normal |  |  |  |  |  |  |  |  |
| Normal |  |  |  |  |  |  |  |  |
| Normal |  |  |  |  |  |  |  |  |
| Normal |  |  |  |  |  |  |  |  |
| Normal |  |  |  |  |  |  |  |  |
| Normal |  |  |  |  |  |  |  |  |
| Normal |  |  |  |  |  |  |  |  |
| Normal |  |  |  |  |  |  |  |  |
| Normal |  |  |  |  |  |  |  |  |
| Normal |  |  |  |  |  |  |  |  |
| Normal |  |  |  |  |  |  |  |  |
| Normal |  |  |  |  |  |  |  |  |
| Normal |  |  |  |  |  |  |  |  |
| Normal |  |  |  |  |  |  |  |  |
| Normal |  |  |  |  |  |  |  |  |
| Normal |  |  |  |  |  |  |  |  |
| Normal |  |  |  |  |  |  |  |  |
| Normal |  |  |  |  |  |  |  |  |
| Normal |  |  |  |  |  |  |  |  |
| Normal |  |  |  |  |  |  |  |  |
| Normal |  |  |  |  |  |  |  |  |
| Normal |  |  |  |  |  |  |  |  |
| Normal |  |  |  |  |  |  |  |  |
| Normal |  |  |  |  |  |  |  |  |
| Normal |  |  |  |  |  |  |  |  |
| Normal |  |  |  |  |  |  |  |  |
| Normal |  |  |  |  |  |  |  |  |
| Normal |  |  |  |  |  |  |  |  |
| Normal |  |  |  |  |  |  |  |  |
| Normal |  |  |  |  |  |  |  |  |
| Normal |  |  |  |  |  |  |  |  |
| Normal |  |  |  |  |  |  |  |  |
| Normal |  |  |  |  |  |  |  |  |
| Normal |  |  |  |  |  |  |  |  |
| Normal |  |  |  |  |  |  |  |  |
| Normal |  |  |  |  |  |  |  |  |
| Normal |  |  |  |  |  |  |  |  |
| Normal |  |  |  |  |  |  |  |  |
| Normal |  |  |  |  |  |  |  |  |
| Normal |  |  |  |  |  |  |  |  |
| Normal |  |  |  |  |  |  |  |  |
| Normal |  |  |  |  |  |  |  |  |
| Normal |  |  |  |  |  |  |  |  |
| Normal |  |  |  |  |  |  |  |  |
| Normal |  |  |  |  |  |  |  |  |
| Normal |  |  |  |  |  |  |  |  |
| Normal |  |  |  |  |  |  |  |  |
| Normal |  |  |  |  |  |  |  |  |
| Normal |  |  |  |  |  |  |  |  |
| Normal |  |  |  |  |  |  |  |  |
| Normal |  |  |  |  |  |  |  |  |
| Normal |  |  |  |  |  |  |  |  |
| Normal |  |  |  |  |  |  |  |  |
| Normal |  |  |  |  |  |  |  |  |
| Normal |  |  |  |  |  |  |  |  |
| Normal |  |  |  |  |  |  |  |  |
| Normal |  |  |  |  |  |  |  |  |
| Normal |  |  |  |  |  |  |  |  |
| Normal |  |  |  |  |  |  |  |  |
| Normal |  |  |  |  |  |  |  |  |
| Normal |  |  |  |  |  |  |  |  |
| Normal |  |  |  |  |  |  |  |  |
| Normal |  |  |  |  |  |  |  |  |
| Normal |  |  |  |  |  |  |  |  |
| Normal |  |  |  |  |  |  |  |  |
| Normal |  |  |  |  |  |  |  |  |
| Normal |  |  |  |  |  |  |  |  |
| Normal |  |  |  |  |  |  |  |  |
| Normal |  |  |  |  |  |  |  |  |
| Normal |  |  |  |  |  |  |  |  |
| Normal |  |  |  |  |  |  |  |  |
| Normal |  |  |  |  |  |  |  |  |
| Normal |  |  |  |  |  |  |  |  |
| Normal |  |  |  |  |  |  |  |  |
| Normal |  |  |  |  |  |  |  |  |
| Normal |  |  |  |  |  |  |  |  |
| Normal |  |  |  |  |  |  |  |  |
| Normal |  |  |  |  |  |  |  |  |
| Normal |  |  |  |  |  |  |  |  |
| Normal |  |  |  |  |  |  |  |  |
| Normal |  |  |  |  |  |  |  |  |
| Normal |  |  |  |  |  |  |  |  |
| Normal |  |  |  |  |  |  |  |  |
| Normal |  |  |  |  |  |  |  |  |
| Normal |  |  |  |  |  |  |  |  |
| Normal |  |  |  |  |  |  |  |  |
| Normal |  |  |  |  |  |  |  |  |
| Normal |  |  |  |  |  |  |  |  |
| Normal |  |  |  |  |  |  |  |  |
| Normal |  |  |  |  |  |  |  |  |
| Normal |  |  |  |  |  |  |  |  |
| Normal |  |  |  |  |  |  |  |  |
| Normal |  |  |  |  |  |  |  |  |
| Normal |  |  |  |  |  |  |  |  |
| Normal |  |  |  |  |  |  |  |  |
| Normal |  |  |  |  |  |  |  |  |
| Normal |  |  |  |  |  |  |  |  |
| Normal |  |  |  |  |  |  |  |  |
| Normal |  |  |  |  |  |  |  |  |
| Normal |  |  |  |  |  |  |  |  |
| Normal |  |  |  |  |  |  |  |  |
| Normal |  |  |  |  |  |  |  |  |
| Normal |  |  |  |  |  |  |  |  |
| Normal |  |  |  |  |  |  |  |  |
| Normal |  |  |  |  |  |  |  |  |
| Normal |  |  |  |  |  |  |  |  |
| Normal |  |  |  |  |  |  |  |  |
| Normal |  |  |  |  |  |  |  |  |
| Normal |  |  |  |  |  |  |  |  |
| Normal |  |  |  |  |  |  |  |  |
| Normal |  |  |  |  |  |  |  |  |
| Normal |  |  |  |  |  |  |  |  |
| Normal |  |  |  |  |  |  |  |  |
| Normal |  |  |  |  |  |  |  |  |
| Normal |  |  |  |  |  |  |  |  |
| Normal |  |  |  |  |  |  |  |  |
| Normal |  |  |  |  |  |  |  |  |
| Normal |  |  |  |  |  |  |  |  |
| Normal |  |  |  |  |  |  |  |  |
| Normal |  |  |  |  |  |  |  |  |
| Normal |  |  |  |  |  |  |  |  |
| Normal |  |  |  |  |  |  |  |  |
| Normal |  |  |  |  |  |  |  |  |
| Normal |  |  |  |  |  |  |  |  |
| Normal |  |  |  |  |  |  |  |  |
| Normal |  |  |  |  |  |  |  |  |
| Normal |  |  |  |  |  |  |  |  |
| Normal |  |  |  |  |  |  |  |  |
| Normal |  |  |  |  |  |  |  |  |
| Normal |  |  |  |  |  |  |  |  |
| Normal |  |  |  |  |  |  |  |  |
| Normal |  |  |  |  |  |  |  |  |
| Normal |  |  |  |  |  |  |  |  |
| Normal |  |  |  |  |  |  |  |  |
| Normal |  |  |  |  |  |  |  |  |
| Normal |  |  |  |  |  |  |  |  |
| Normal |  |  |  |  |  |  |  |  |
| Normal |  |  |  |  |  |  |  |  |
| Normal |  |  |  |  |  |  |  |  |
| Normal |  |  |  |  |  |  |  |  |
| Normal |  |  |  |  |  |  |  |  |
| Normal |  |  |  |  |  |  |  |  |
| Normal |  |  |  |  |  |  |  |  |
| Normal |  |  |  |  |  |  |  |  |
| Normal |  |  |  |  |  |  |  |  |
| Normal |  |  |  |  |  |  |  |  |
| Normal |  |  |  |  |  |  |  |  |
| Normal |  |  |  |  |  |  |  |  |
| Normal |  |  |  |  |  |  |  |  |
| Normal |  |  |  |  |  |  |  |  |
| Normal |  |  |  |  |  |  |  |  |
| Normal |  |  |  |  |  |  |  |  |
| Normal |  |  |  |  |  |  |  |  |
| Normal |  |  |  |  |  |  |  |  |
| Normal |  |  |  |  |  |  |  |  |
| Normal |  |  |  |  |  |  |  |  |
| Normal |  |  |  |  |  |  |  |  |
| Normal |  |  |  |  |  |  |  |  |
| Normal |  |  |  |  |  |  |  |  |
| Normal |  |  |  |  |  |  |  |  |
| Normal |  |  |  |  |  |  |  |  |
| Normal |  |  |  |  |  |  |  |  |
| Normal |  |  |  |  |  |  |  |  |
| Normal |  |  |  |  |  |  |  |  |
| Normal |  |  |  |  |  |  |  |  |
| Normal |  |  |  |  |  |  |  |  |
| Normal |  |  |  |  |  |  |  |  |
| Normal |  |  |  |  |  |  |  |  |
| Normal |  |  |  |  |  |  |  |  |
| Normal |  |  |  |  |  |  |  |  |
| Normal |  |  |  |  |  |  |  |  |
| Normal |  |  |  |  |  |  |  |  |
| Normal |  |  |  |  |  |  |  |  |
| Normal |  |  |  |  |  |  |  |  |
| Normal |  |  |  |  |  |  |  |  |
| Normal |  |  |  |  |  |  |  |  |
| Normal |  |  |  |  |  |  |  |  |
| Normal |  |  |  |  |  |  |  |  |
| Normal |  |  |  |  |  |  |  |  |
| Normal |  |  |  |  |  |  |  |  |
| Normal |  |  |  |  |  |  |  |  |
| Normal |  |  |  |  |  |  |  |  |
| Normal |  |  |  |  |  |  |  |  |
| Normal |  |  |  |  |  |  |  |  |
| Normal |  |  |  |  |  |  |  |  |
| Normal |  |  |  |  |  |  |  |  |
| Normal |  |  |  |  |  |  |  |  |
| Normal |  |  |  |  |  |  |  |  |
| Normal |  |  |  |  |  |  |  |  |
| Normal |  |  |  |  |  |  |  |  |
| Normal |  |  |  |  |  |  |  |  |
| Normal |  |  |  |  |  |  |  |  |
| Normal |  |  |  |  |  |  |  |  |
| Normal |  |  |  |  |  |  |  |  |
| Normal |  |  |  |  |  |  |  |  |
| Normal |  |  |  |  |  |  |  |  |
| Normal |  |  |  |  |  |  |  |  |
| Normal |  |  |  |  |  |  |  |  |
| Normal |  |  |  |  |  |  |  |  |
| Normal |  |  |  |  |  |  |  |  |
| Normal |  |  |  |  |  |  |  |  |
| Normal |  |  |  |  |  |  |  |  |
| Normal |  |  |  |  |  |  |  |  |
| Normal |  |  |  |  |  |  |  |  |
| Normal |  |  |  |  |  |  |  |  |
| Normal |  |  |  |  |  |  |  |  |
| Normal |  |  |  |  |  |  |  |  |
| Normal |  |  |  |  |  |  |  |  |
| Normal |  |  |  |  |  |  |  |  |
| Normal |  |  |  |  |  |  |  |  |
| Normal |  |  |  |  |  |  |  |  |
| Normal |  |  |  |  |  |  |  |  |
| Normal |  |  |  |  |  |  |  |  |
| Normal |  |  |  |  |  |  |  |  |
| Normal |  |  |  |  |  |  |  |  |
| Normal |  |  |  |  |  |  |  |  |
| Normal |  |  |  |  |  |  |  |  |
| Normal |  |  |  |  |  |  |  |  |
| Normal |  |  |  |  |  |  |  |  |
| Normal |  |  |  |  |  |  |  |  |
| Normal |  |  |  |  |  |  |  |  |
| Normal |  |  |  |  |  |  |  |  |
| Normal |  |  |  |  |  |  |  |  |
| Normal |  |  |  |  |  |  |  |  |
| Normal |  |  |  |  |  |  |  |  |
| Normal |  |  |  |  |  |  |  |  |
| Normal |  |  |  |  |  |  |  |  |
| Normal |  |  |  |  |  |  |  |  |
| Normal |  |  |  |  |  |  |  |  |
| Normal |  |  |  |  |  |  |  |  |
| Normal |  |  |  |  |  |  |  |  |
| Normal |  |  |  |  |  |  |  |  |
| Normal |  |  |  |  |  |  |  |  |
| Normal |  |  |  |  |  |  |  |  |
| Normal |  |  |  |  |  |  |  |  |
| Normal |  |  |  |  |  |  |  |  |
| Normal |  |  |  |  |  |  |  |  |
| Normal |  |  |  |  |  |  |  |  |
| Normal |  |  |  |  |  |  |  |  |
| Normal |  |  |  |  |  |  |  |  |
| Normal |  |  |  |  |  |  |  |  |
| Normal |  |  |  |  |  |  |  |  |
| Normal |  |  |  |  |  |  |  |  |
| Normal |  |  |  |  |  |  |  |  |
| Normal |  |  |  |  |  |  |  |  |
| Normal |  |  |  |  |  |  |  |  |
| Normal |  |  |  |  |  |  |  |  |
| Normal |  |  |  |  |  |  |  |  |
| Normal |  |  |  |  |  |  |  |  |
| Normal |  |  |  |  |  |  |  |  |
| Normal |  |  |  |  |  |  |  |  |
| Normal |  |  |  |  |  |  |  |  |
| Normal |  |  |  |  |  |  |  |  |
| Normal |  |  |  |  |  |  |  |  |

**Table S5** The 47 immune checkpoint-related genes;

| immune checkpoint-related genes |
| --- |
| IDO1 |
| LAG3 |
| CTLA4 |
| TNFRSF9 |
| ICOS |
| CD80 |
| PDCD1LG2 |
| TIGIT |
| CD70 |
| TNFSF9 |
| ICOSLG |
| KIR3DL1 |
| CD86 |
| PDCD1 |
| LAIR1 |
| TNFRSF8 |
| TNFSF15 |
| TNFRSF14 |
| IDO2 |
| CD276 |
| CD40 |
| TNFRSF4 |
| TNFSF14 |
| HHLA2 |
| CD244 |
| CD274 |
| HAVCR2 |
| CD27 |
| BTLA |
| LGALS9 |
| TMIGD2 |
| CD28 |
| CD48 |
| TNFRSF25 |
| CD40LG |
| ADORA2A |
| VTCN1 |
| CD160 |
| CD44 |
| TNFSF18 |
| TNFRSF18 |
| BTNL2 |
| C10orf54 |
| CD200R1 |
| TNFSF4 |
| CD200 |
| NRP1 |

**Table S6** The 15 overlapping genes of the two sets of genes associated with disulfidptosis;

| SLC7A11 |
| --- |
| INF2 |
| CD2AP |
| PDLIM1 |
| ACTN4 |
| MYH9 |
| MYH10 |
| IQGAP1 |
| FLNA |
| FLNB |
| TLN1 |
| MYL6 |
| ACTB |
| DSTN |
| CAPZB. |

**Table S7** The results of the Gene Set Enrichment Analysis (GSEA)

| ID | Description | setSize | enrichmentScore | NES | pvalue | p.adjust | qvalue | rank | leading_edge | core_enrichment |  |
| --- | --- | --- | --- | --- | --- | --- | --- | --- | --- | --- | --- |
| hsa00190 | hsa00190 | Oxidative phosphorylation | 11 | -0.660287081 | -1.893685524 | 0.0051731 | 0.0051731 | 0.181052911 | 225 | tags=100%, list=35%, signal=66% | 4717/4714/10063/4695/1329/1349/4729/1345/4696/4694 |
| hsa04932 | hsa04932 | Non-alcoholic fatty liver disease | 13 | -0.602125515 | -1.807624665 | 0.010928962 | 0.010928962 | 0.181052911 | 225 | tags=92%, list=35%, signal=61% | 4716/4717/4714/4695/1329/1349/4729/1345/4696/4694/5296/1571 |
| hsa05208 | hsa05208 | Chemical carcinogenesis - reactive oxygen species | 18 | -0.537675945 | -1.771694782 | 0.011727291 | 0.011727291 | 0.181052911 | 225 | tags=83%, list=35%, signal=56% | 4716/4717/4258/4714/4695/1329/1349/4729/1345/4696/27035/4694/2946/5296/1571 |
| hsa05206 | hsa05206 | MicroRNAs in cancer | 11 | 0.582424156 | 1.657021968 | 0.027130225 | 0.027130225 | 0.303710095 | 160 | tags=73%, list=25%, signal=55% | 3371/6541/4609/10253/1021/6464/4170/5594 |
| hsa05415 | hsa05415 | Diabetic cardiomyopathy | 13 | -0.542512881 | -1.628663194 | 0.032786885 | 0.032786885 | 0.303710095 | 225 | tags=85%, list=35%, signal=56% | 4716/4717/4714/4695/1329/1349/4729/1345/4696/4694/5296 |
| hsa04141 | hsa04141 | Protein processing in endoplasmic reticulum | 23 | 0.416252971 | 1.478091274 | 0.068671272 | 0.068671272 | 0.443169204 | 222 | tags=65%, list=35%, signal=44% | 10130/81567/10525/9871/56886/3312/824/6747/3703/9601/1965/10802/9695/56893/10484 |
| hsa05130 | hsa05130 | Pathogenic Escherichia coli infection | 13 | 0.4944 | 1.49156417 | 0.076056895 | 0.076056895 | 0.443169204 | 329 | tags=100%, list=52%, signal=49% | 1364/3654/10376/10109/9871/3688/5594/10802/4627/1147/2768/10097/23191 |
| hsa04151 | hsa04151 | PI3K-Akt signaling pathway | 21 | 0.413919742 | 1.437200335 | 0.088633152 | 0.088633152 | 0.443169204 | 118 | tags=38%, list=18%, signal=32% | 26291/3371/3673/4609/1021/3688/3915/3912 |
| hsa05222 | hsa05222 | Small cell lung cancer | 10 | 0.527572604 | 1.451610089 | 0.092503023 | 0.092503023 | 0.443169204 | 145 | tags=70%, list=23%, signal=55% | 3673/4609/1021/3688/3915/3912/1647 |

**Table S8** The q-pcr raw data.

| Well | Well Position | Sample Name | Sample Color | Biogroup Name | Biogroup Color | Target Name | Target Color | Task | Reporter | Quencher | Quantity | Comments |
| --- | --- | --- | --- | --- | --- | --- | --- | --- | --- | --- | --- | --- |
| 1 | A1 | PATIENT1 T | SLC7A11 | 28.358 |  |  |  |  |  |  |  |  |
| 2 | A2 | PATIENT1 T | SLC7A11 | 29.242 |  |  |  |  |  |  |  |  |
| 3 | A3 | PATIENT1 T | SLC7A11 | 29.269 |  |  |  |  |  |  |  |  |
| 4 | A4 | PATIENT1 T | GAPDH | 25.674 |  |  |  |  |  |  |  |  |
| 5 | A5 | PATIENT1 T | GAPDH | 25.898 |  |  |  |  |  |  |  |  |
| 6 | A6 | PATIENT1 T | GAPDH | 25.800 |  |  |  |  |  |  |  |  |
| 7 | A7 | PATIENT2 T | SLC7A11 | 28.899 |  |  |  |  |  |  |  |  |
| 8 | A8 | PATIENT2 T | SLC7A11 | 28.812 |  |  |  |  |  |  |  |  |
| 9 | A9 | PATIENT2 T | SLC7A11 | 28.214 |  |  |  |  |  |  |  |  |
| 10 | A10 |  |  |  |  |  |  |  |  |  |  |  |
| 11 | A11 |  |  |  |  |  |  |  |  |  |  |  |
| 12 | A12 |  |  |  |  |  |  |  |  |  |  |  |
| 13 | B1 | PATIENT2 T | GAPDH | 25.610 |  |  |  |  |  |  |  |  |
| 14 | B2 | PATIENT2 T | GAPDH | 25.640 |  |  |  |  |  |  |  |  |
| 15 | B3 | PATIENT2 T | GAPDH | 25.569 |  |  |  |  |  |  |  |  |
| 16 | B4 | PATIENT3 T | SLC7A11 | 32.254 |  |  |  |  |  |  |  |  |
| 17 | B5 | PATIENT3 T | SLC7A11 | 31.315 |  |  |  |  |  |  |  |  |
| 18 | B6 | PATIENT3 T | SLC7A11 | 31.347 |  |  |  |  |  |  |  |  |
| 19 | B7 | PATIENT3 T | GAPDH | 29.664 |  |  |  |  |  |  |  |  |
| 20 | B8 | PATIENT3 T | GAPDH | 29.799 |  |  |  |  |  |  |  |  |
| 21 | B9 | PATIENT3 T | GAPDH | 29.596 |  |  |  |  |  |  |  |  |
| 22 | B10 |  |  |  |  |  |  |  |  |  |  |  |
| 23 | B11 |  |  |  |  |  |  |  |  |  |  |  |
| 24 | B12 |  |  |  |  |  |  |  |  |  |  |  |
| 25 | D1 | PATIENT4 T | SLC7A11 | 32.578 |  |  |  |  |  |  |  |  |
| 26 | D2 | PATIENT4 T | SLC7A11 | 31.468 |  |  |  |  |  |  |  |  |
| 27 | D3 | PATIENT4 T | SLC7A11 | 32.796 |  |  |  |  |  |  |  |  |
| 28 | D4 | PATIENT4 T | GAPDH | 28.167 |  |  |  |  |  |  |  |  |
| 29 | D5 | PATIENT4 T | GAPDH | 28.023 |  |  |  |  |  |  |  |  |
| 30 | D6 | PATIENT4 T | GAPDH | 27.438 |  |  |  |  |  |  |  |  |
| 31 | D7 | PATIENT5 T | SLC7A11 | 29.975 |  |  |  |  |  |  |  |  |
| 32 | D8 | PATIENT5 T | SLC7A11 | 29.401 |  |  |  |  |  |  |  |  |
| 33 | D9 | PATIENT5 T | SLC7A11 | 29.749 |  |  |  |  |  |  |  |  |
| 34 | D10 |  |  |  |  |  |  |  |  |  |  |  |
| 35 | D11 |  |  |  |  |  |  |  |  |  |  |  |
| 36 | D12 |  |  |  |  |  |  |  |  |  |  |  |
| 37 | E1 | PATIENT5 T | GAPDH | 25.380 |  |  |  |  |  |  |  |  |
| 38 | E2 | PATIENT5 T | GAPDH | 25.483 |  |  |  |  |  |  |  |  |
| 39 | E3 | PATIENT5 T | GAPDH | 25.453 |  |  |  |  |  |  |  |  |
| 40 | E4 | PATIENT1 N | SLC7A11 | 33.178 |  |  |  |  |  |  |  |  |
| 41 | E5 | PATIENT1 N | SLC7A11 | 36.591 |  |  |  |  |  |  |  |  |
| 42 | E6 | PATIENT1 N | SLC7A11 | 33.776 |  |  |  |  |  |  |  |  |
| 43 | E7 | PATIENT1 N | GAPDH | 28.939 |  |  |  |  |  |  |  |  |
| 44 | E8 | PATIENT1 N | GAPDH | 29.119 |  |  |  |  |  |  |  |  |
| 45 | E9 | PATIENT1 N | GAPDH | 29.294 |  |  |  |  |  |  |  |  |
| 46 | E10 |  |  |  |  |  |  |  |  |  |  |  |
| 47 | E11 |  |  |  |  |  |  |  |  |  |  |  |
| 48 | E12 |  |  |  |  |  |  |  |  |  |  |  |
| 49 | F1 | PATIENT2 N | SLC7A11 | 36.162 |  |  |  |  |  |  |  |  |
| 50 | F2 | PATIENT2 N | SLC7A11 | 34.783 |  |  |  |  |  |  |  |  |
| 51 | F3 | PATIENT2 N | SLC7A11 | 30.935 |  |  |  |  |  |  |  |  |
| 52 | F4 | PATIENT2 N | GAPDH | 29.088 |  |  |  |  |  |  |  |  |
| 53 | F5 | PATIENT2 N | GAPDH | 29.138 |  |  |  |  |  |  |  |  |
| 54 | F6 | PATIENT2 N | GAPDH | 29.127 |  |  |  |  |  |  |  |  |
| 55 | F7 | PATIENT3 N | SLC7A11 | 33.208 |  |  |  |  |  |  |  |  |
| 56 | F8 | PATIENT3 N | SLC7A11 | 29.931 |  |  |  |  |  |  |  |  |
| 57 | F9 | PATIENT3 N | SLC7A11 | 28.890 |  |  |  |  |  |  |  |  |
| 58 | F10 |  |  |  |  |  |  |  |  |  |  |  |
| 59 | F11 |  |  |  |  |  |  |  |  |  |  |  |
| 60 | F12 |  |  |  |  |  |  |  |  |  |  |  |
| 61 | G1 | PATIENT3 N | GAPDH | 25.596 |  |  |  |  |  |  |  |  |
| 62 | G2 | PATIENT3 N | GAPDH | 25.748 |  |  |  |  |  |  |  |  |
| 63 | G3 | PATIENT3 N | GAPDH | 25.722 |  |  |  |  |  |  |  |  |
| 64 | G4 | PATIENT4 N | SLC7A11 | 31.478 |  |  |  |  |  |  |  |  |
| 65 | G5 | PATIENT4 N | SLC7A11 | 31.873 |  |  |  |  |  |  |  |  |
| 66 | G6 | PATIENT4 N | SLC7A11 | 31.602 |  |  |  |  |  |  |  |  |
| 67 | G7 | PATIENT4 N | GAPDH | 24.781 |  |  |  |  |  |  |  |  |
| 68 | G8 | PATIENT4 N | GAPDH | 24.782 |  |  |  |  |  |  |  |  |
| 69 | G9 | PATIENT4 N | GAPDH | 24.844 |  |  |  |  |  |  |  |  |
| 70 | G10 |  |  |  |  |  |  |  |  |  |  |  |
| 71 | G11 |  |  |  |  |  |  |  |  |  |  |  |
| 72 | G12 |  |  |  |  |  |  |  |  |  |  |  |
| 73 | H1 | PATIENT5 N | SLC7A11 | 33.663 |  |  |  |  |  |  |  |  |
| 74 | H2 | PATIENT5 N | SLC7A11 | 31.514 |  |  |  |  |  |  |  |  |
| 75 | H3 | PATIENT5 N | SLC7A11 | 32.528 |  |  |  |  |  |  |  |  |
| 76 | H4 | PATIENT5 N | GAPDH | 25.765 |  |  |  |  |  |  |  |  |
| 77 | H5 | PATIENT5 N | GAPDH | 25.857 |  |  |  |  |  |  |  |  |
| 78 | H6 | PATIENT5 N | GAPDH | 25.868 |  |  |  |  |  |  |  |  |
